# Impaired glucose metabolism and the risk of vascular events and mortality after ischemic stroke: A systematic review and meta-analysis

**DOI:** 10.1186/s12933-024-02413-w

**Published:** 2024-08-31

**Authors:** Nurcennet Kaynak, Valentin Kennel, Torsten Rackoll, Daniel Schulze, Matthias Endres, Alexander H. Nave

**Affiliations:** 1 Center for Stroke Research Berlin (CSB), Charité– Universitätsmedizin Berlin, corporate member of Freie Universität Berlin and Humboldt Universität zu Berlin, Berlin, Germany; 2 Department of Neurology with Experimental Neurology, Charité– Universitätsmedizin Berlin, corporate member of Freie Universität Berlin and Humboldt Universität zu Berlin, Charitéplatz 1, 10117 Berlin, Germany; 3 Berlin Institute of Health at Charité, Charité– Universitätsmedizin Berlin, corporate member of Freie Universität Berlin and Humboldt Universität zu Berlin, Berlin, Germany; 4 German Center for Neurodegenerative Diseases (DZNE), partner site Berlin, Berlin, Germany; 5 German Center for Cardiovascular Research (DZHK), partner site Berlin, Berlin, Germany; 6 Berlin Institute of Health (BIH) QUEST Center for Responsible Research, Charité– Universitätsmedizin Berlin, corporate member of Freie Universität Berlin and Humboldt Universität zu Berlin, Berlin, Germany; 7 Department of Biometry and Clinical Epidemiology, Charité-Universitätsmedizin Berlin, Corporate Member of Freie Universität Berlin und Humboldt-Universität zu Berlin, Berlin, Germany; 8 German Center for Mental Health (DZPG), partner site Berlin, Berlin, Germany

**Keywords:** Ischemic stroke, Diabetes, Prediabetes, Insulin resistance, Vascular events, Mortality

## Abstract

**Background:**

Diabetes mellitus (DM), prediabetes, and insulin resistance are highly prevalent in patients with ischemic stroke (IS). DM is associated with higher risk for poor outcomes after IS.

**Objective:**

Investigate the risk of recurrent vascular events and mortality associated with impaired glucose metabolism compared to normoglycemia in patients with IS and transient ischemic attack (TIA).

**Methods:**

Systematic literature search was performed in PubMed, Embase, Cochrane Library on 21st March 2024 and via citation searching. Studies that comprised IS or TIA patients and exposures of impaired glucose metabolism were eligible. Study Quality Assessment Tool was used for risk of bias assessment. Covariate adjusted outcomes were pooled using random-effects meta-analysis.

**Main outcomes:**

Recurrent stroke, cardiac events, cardiovascular and all-cause mortality and composite of vascular outcomes.

**Results:**

Of 10,974 identified studies 159 were eligible. 67% had low risk of bias. DM was associated with an increased risk for composite events (pooled HR (pHR) including 445,808 patients: 1.58, 95% CI 1.34–1.85, I^2^ = 88%), recurrent stroke (pHR including 1.161.527 patients: 1.42 (1.29–1.56, I^2^ = 92%), cardiac events (pHR including 443,863 patients: 1.55, 1.50–1.61, I^2^ = 0%), and all-cause mortality (pHR including 1.031.472 patients: 1.56, 1.34–1.82, I^2^ = 99%). Prediabetes was associated with an increased risk for composite events (pHR including 8,262 patients: 1.50, 1.15–1.96, I^2^ = 0%) and recurrent stroke (pHR including 10,429 patients: 1.50, 1.18–1.91, I^2^ = 0), however, not with mortality (pHR including 9,378 patients, 1.82, 0.73–4.57, I^2^ = 78%). Insulin resistance was associated with recurrent stroke (pHR including 21,363 patients: 1.56, 1.19–2.05, I^2^ = 55%), but not with mortality (pHR including 21,363 patients: 1.31, 0.66–2.59, I^2^ = 85%).

**Discussion:**

DM is associated with a 56% increased relative risk of death after IS and TIA. Risk estimates regarding recurrent events are similarly high between prediabetes and DM, indicating high cardiovascular risk burden already in precursor stages of DM. There was a high heterogeneity across most outcomes.

**Supplementary Information:**

The online version contains supplementary material available at 10.1186/s12933-024-02413-w.

## Introduction

Ischemic stroke (IS) is associated with high mortality and high risk of recurrent vascular events worldwide [1–3]. Despite adequate secondary prevention, about 11% of patients suffer a recurrent stroke within the first year [4]. Diabetes mellitus (DM) is a highly prevalent cardiovascular risk factor and is present in about one-third of IS patients [5, 6]. Stroke prevention guidelines recommend screening for unrecognized DM after IS [7]. Besides DM, other forms of impaired glucose metabolism (IGM), such as prediabetes and insulin resistance (IR) have been gaining importance over the last decades in terms of their association with increased cardiovascular risk [8]. Prediabetes, comprising impaired fasting glucose and impaired glucose tolerance, represents a hyperglycemic condition of patients not yet within the diabetic range [9]. In comparison, IR constitutes a pathophysiological mechanism, which usually precedes and coexists with both DM and prediabetes [10]. Observational studies report that 70% of the patients with IS have either DM (46%) or prediabetes (24%), and 50% of those who have no DM at baseline have IR [11, 12].

Considering that the majority of patients with stroke have some form of IGM, it represents an important aspect of secondary stroke prevention. Numerous studies, including systematic reviews, have shown the association between DM and prediabetes and stroke recurrence [13–15]. However, only few studies have looked at composite vascular events as an outcome. Furthermore, mortality risk associated with DM after stroke has not been addressed in previous meta-analyses. A comprehensive systematic approach is needed to identify and compare risks associated with composite vascular events and mortality after IS and TIA between different forms of IGM.

Stroke prevention guidelines recommend the use of new generation antidiabetics based on the finding that these agents demonstrated cardiovascular protective effects in patients with previous cardiovascular disease including stroke [7]. However, only the minority of patients had a history of stroke and subgroup analyses of patients with a previous IS or TIA remained mostly inconclusive [16, 17]. In contrast, in the IRIS Trial only patients with IR and a recent IS or TIA were included [18]. Despite the lower risk of cardiovascular events associated with pioglitazone, the high risk of adverse events restricted the clinical implication of the drug. Currently, it remains unclear which pharmacological treatments are beneficial in terms of secondary stroke prevention in patients with acute or subacute IS or TIA and different forms of IGM.

Identifying increased cardiovascular risk not only in DM but also other forms of IGM would capture a greater population at risk and eventually prompt implementation of secondary preventive measures. We conducted a systematic literature review and meta-analysis to extend our knowledge on the burden of IGM in patients with IS and TIA in the context of cardiovascular events and mortality.

## Methods

This manuscript adheres to the PRISMA guideline [19]. Study protocol was pre-registered in open science framework in 2021 [20].

### Information sources

We conducted a systematic literature search on Medline via Pubmed, Ovid via Embase, and Cochrane Library that was last updated on March 21, 2024. Search terms included “diabetes”, “prediabetes”, “insulin resistance”, “stroke” and “transient ischemic attack”, restricted to English language. See full search strategy in supplementary material methods. Reference lists of previous systematic reviews and of studies included in our review were searched manually.

### Study selection and data extraction

Screening was performed by two reviewers independently (NK and VK) and consensus was reached with two additional reviewers (TR and AHN) in case of disagreement. Eligible studies were observational studies that included patients within 3 months after an IS or TIA and reported at least one of the following outcomes: composite vascular events, recurrent stroke, cardiovascular and all-cause mortality, cardiac events including but not limited to myocardial infarction, all regardless of follow-up duration (see supplementary Table 1 for the eligibility criteria). Composite events comprised at least stroke, cardiac events, and cardiovascular death. Studies were required to report hazard ratios (HR), odds ratios (OR), or risk ratios using a multivariable model. Exposures of interest were DM, prediabetes and IR, which were included independently of the definition used in the respective study. Additionally, we screened for studies that compared the use of an antidiabetic therapy to placebo or another antidiabetic therapy within the same population and outcomes mentioned above, regardless of study design.

Data extraction and assessment of risk of bias were performed by one reviewer (NK) and the internal validity was checked with a second reviewer (VK) for a random sample of 10% of studies. Interrater reliability was calculated. Authors were contacted via email if substantial outcome data were lacking, unclear or discrepant. Risk of bias assessment was made using the Study Quality Assessment Tool of National Heart, Lung, and Blood Institute [21]. A detailed methodological description can be found in the methods section of the supplementary material.

### Data synthesis

We performed random effects meta-analyses with the restricted maximum likelihood estimator method after grouping studies into outcome measures HR for each study outcome. OR were pooled using meta-regression with follow-up duration as moderator and with random effects meta-analysis if moderator showed no significant effect (p < 0.05). Studies used different sets of covariates that included sociodemographic and clinical characteristics. We included the effect size from the models with the most adjusting factors available. We calculated the 95% confidence interval (CI) and prediction intervals. Prediction intervals describe the expected range of future study results, while confidence intervals relate to the precision of the aggregated effect. Multi-level meta-analysis was performed if multiple subgroups from a single study were included in the analysis. Furthermore, we performed meta-analyses of absolute risks derived from event numbers for each outcome and exposure group, whenever such data were reported. Heterogeneity was assessed using Cochran’s Q and I^2^ and was assumed present when p < 0.05 or I^2^ > 50% [22]. Results of meta-analyses were visualized using forest plots. Subgroup analyses were conducted based on history of previous stroke (first-ever event, yes/no) and type of ischemic event (IS/TIA/both). Subgroup analyses based on sex were not conducted because the studies included both sexes in their analyses, and individual patient data were not available. As a sensitivity analysis, we conducted meta-analyses using unadjusted odds ratios. Publication bias was assessed by funnel plots and Egger´s regression. Statistical calculations were performed using the Software R Version 4.0.2 with the package “Metafor” [23]. Studies investigating the association between antidiabetic therapies and recurrent cardiovascular events after IS or TIA were summarized narratively.

## Results

### Systematic literature search

**Fig. 1:**
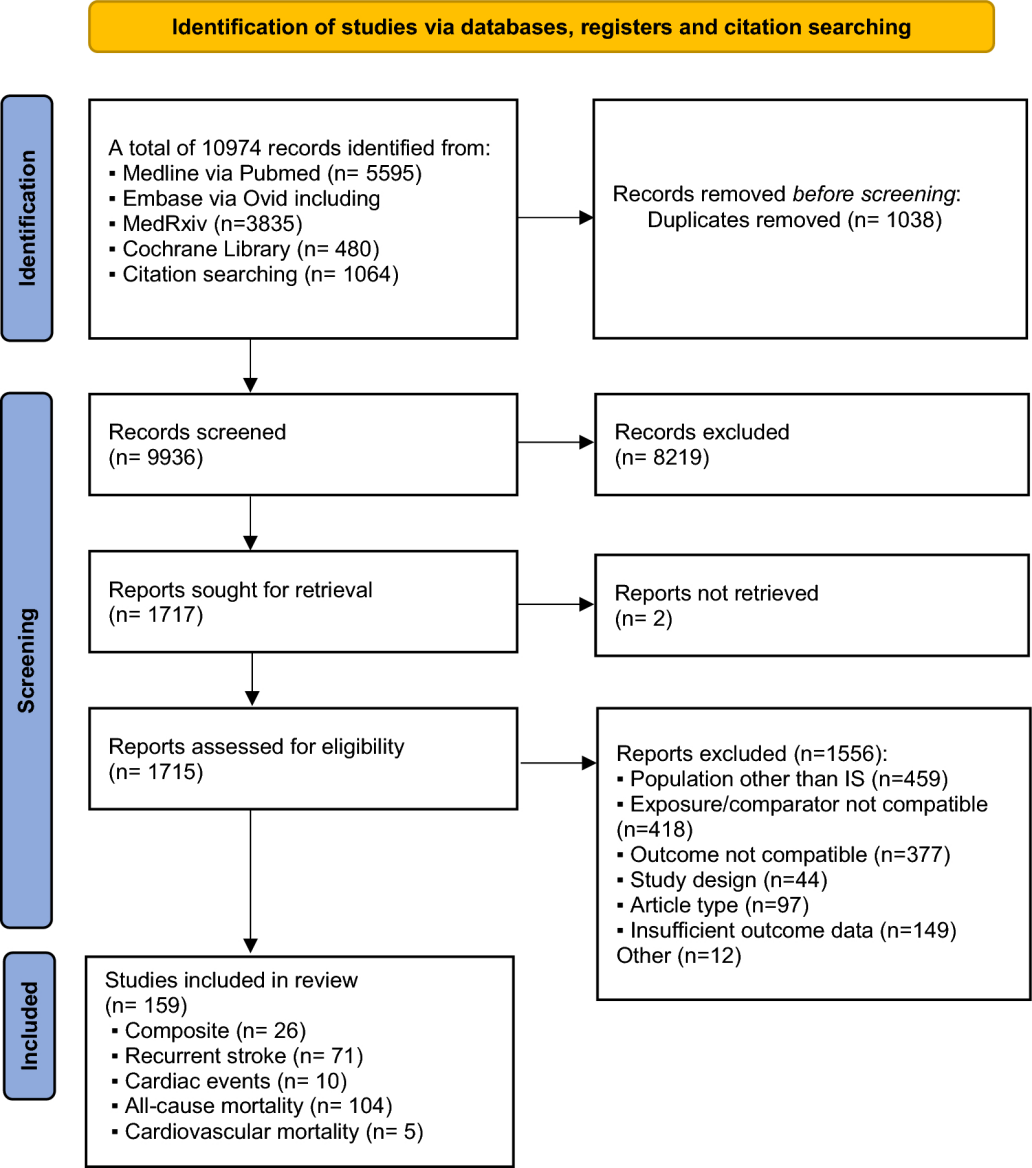
Flowchart of the screening and selection process of the systematic review

The systematic literature search yielded 10,974 records. After screening titles and abstracts, 8,219 records were excluded, and 1,717 records were further screened based on full texts (Fig. 1). Finally, 159 studies met the eligibility criteria (supplementary references). Of those, 26 reported data for composite outcome, 71 for recurrent stroke, 10 for cardiac events, 104 for all-cause mortality, and five for cardiovascular mortality (Table 1). During data extraction an inter-rater reliability of 90% was reached. Authors of twenty-six studies were contacted for missing information, and seven of them provided the requested data. Most studies were observational studies (n = 146), and others were post-hoc analyses of randomized trials (n = 13). Follow-up duration ranged from end-of-hospital-stay to longer than 20 years. The diagnostic criteria used for DM varied highly including based on medical records or medication history only (n = 61), laboratory biomarkers only (n = 14) and both (n = 50). Twenty-one studies did not report the definition used. Prediabetes was defined either according to American Diabetes Association [24] or World Health Organization criteria [25], whereas one study defined prediabetes as a non-fasting glucose level of 140–198 mg/dL. IR was quantified using: HOMA-IR, Triglyceride-Glucose Index, Matsuda Insulin Sensitivity Index, Glucose/Insulin Ratio, QUICKI Index, and estimated glucose disposal rate. Overall, 67% (n = 107) of the included studies were rated as having good quality of evidence, 27% (n = 43) as fair and 6% (n = 9) as poor (supplementary Fig. 1). Study characteristics are presented in supplementary Table 2.

**Table 1:** Study characteristics of the systematic review

Outcomes per exposure	n	Age (Mean)	Sex % (Male)	Number of studies that included a population with			Number of studies with follow-up duration		Number of studies with quality of evidence		
				IS	TIA	Both	≤ 1 y	> 1 y	G	F	P
**Composite outcome**											
Diabetes	24	61.2	58.2	10	2	12	7	17	19	5	0
Prediabetes	3	56.0	58.5	0	0	3	1	2	2	1	0
Insulin Resistance	2	62.0	68.2	2	0	0	2	0	1	1	0
**Recurrent stroke**											
Diabetes	63	63.7	58.5	41	8	14	39	24	45	16	2
Prediabetes	4	57.6	59.8	1	3	0	2	2	3	1	0
Insulin Resistance	7	65.0	64.1	7	0	0	7	0	5	2	0
**Cardiac events**											
Diabetes	10	63.6	55.5	7	0	3	5	5	8	2	0
Prediabetes	1	65.1	65.0	0	0	1	1	0	1	0	0
Insulin Resistance	0	–	–	–	–	–	–	–	–	–	–
**Cardiovascular mortality**											
Diabetes	5	75.5*	51.4	4	1	0	2	3	4	0	1
Prediabetes	0	–	–	–	–	–	–	–	–	–	–
Insulin Resistance	1	61.5	55.0	1	0	0	0	1	1	0	0
**All-cause mortality**											
Diabetes	94	68.4	64.4	82	4	8	65	28	64	24	6
Prediabetes	6	67.5	57.0	5	0	1	6	0	4	2	0
Insulin Resistance	10	63.5	62.5	10	0	0	9	1	7	3	0

*n* number of studies; *NA* not applicable; *g* good, *f* fair; *p* poor

*Median age

### Association of IGM with cardiovascular events

#### Composite vascular events

Twenty-four studies were eligible for the exposure DM, three studies for prediabetes and two studies for IR. Five studies reporting data from the same cohort were excluded, resulting in 19 eligible studies for the exposure DM (16 reported HR, three reported OR; see supplementary Table 3). Except for one study reporting a 3-month follow-up period, all studies reported at least 1-year follow-up. One study that assessed incident DM during follow-up opposed to pre-existing DM as an exposure was not included in the analysis [26].

**Fig. 2:**
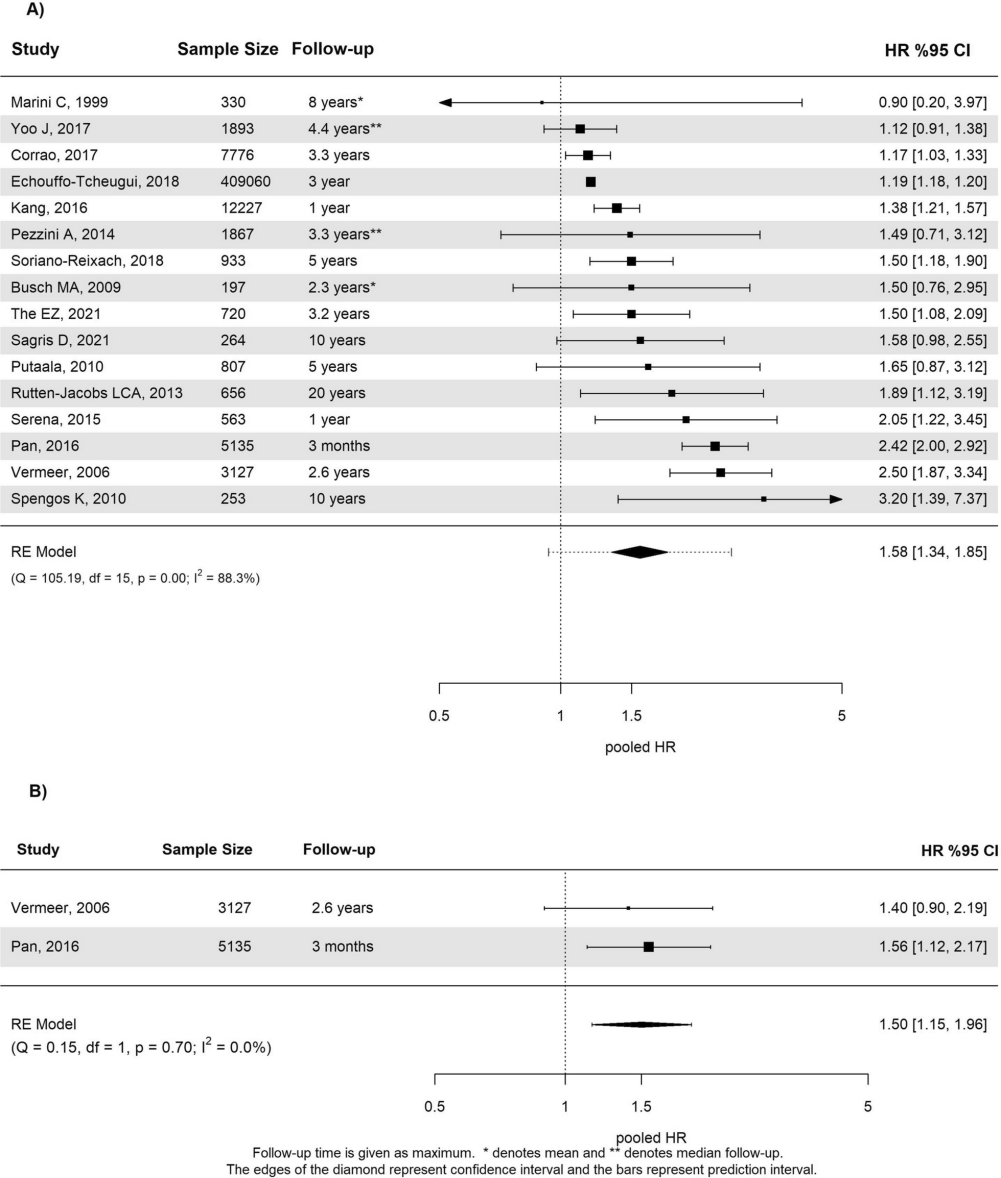
**a** Forest plot for the meta-analysis of studies that reported the association of diabetes with composite outcome. **b** Forest plot for the meta-analysis of studies that reported the association of prediabetes with composite outcome

Presence of DM was statistically significantly associated with an increased risk of composite vascular events with a pooled HR (pHR) of 1.58 (95% confidence interval (CI) 1.34 to 1.85, I^2^ = 88%) including 445,808 patients (Fig. 2A) and a pooled OR (pOR) of 1.87 (95% CI 0.76 to 4.60, I^2^ = 64%) including 1,609 patients. No publication bias was observed (supplementary Fig. 2). The meta-analysis of absolute risks reported in seven studies revealed that during a mean follow-up of three years, 43% (95% CI 23% to 64%) of stroke patients with DM reached a composite endpoint of a recurrent cardiovascular event or death. This rate was 17% (95% CI 3% to 31%) in patients without DM (supplementary Table 4).

Meta-analysis of two studies showed an increased risk of composite events associated with prediabetes with a pHR of 1.50 (95% CI 1.15 to 1.96, I^2^ = 0%; Fig. 2B) in 8,262 patients. An absolute risk of 31% (95% CI 12% to 50%) and 7% (95% CI 5% to 10%) was observed in the group of patients with and without prediabetes, respectively. IR was reported in two studies, which were derived from the same cohort. One of the studies demonstrated no association between high IR and composite vascular events [27]. In the other study, which only encompassed patients without DM, increased IR based on HOMA-IR was statistically significantly associated with an increased risk for vascular events [28].

#### Recurrent stroke

**Fig. 3:**
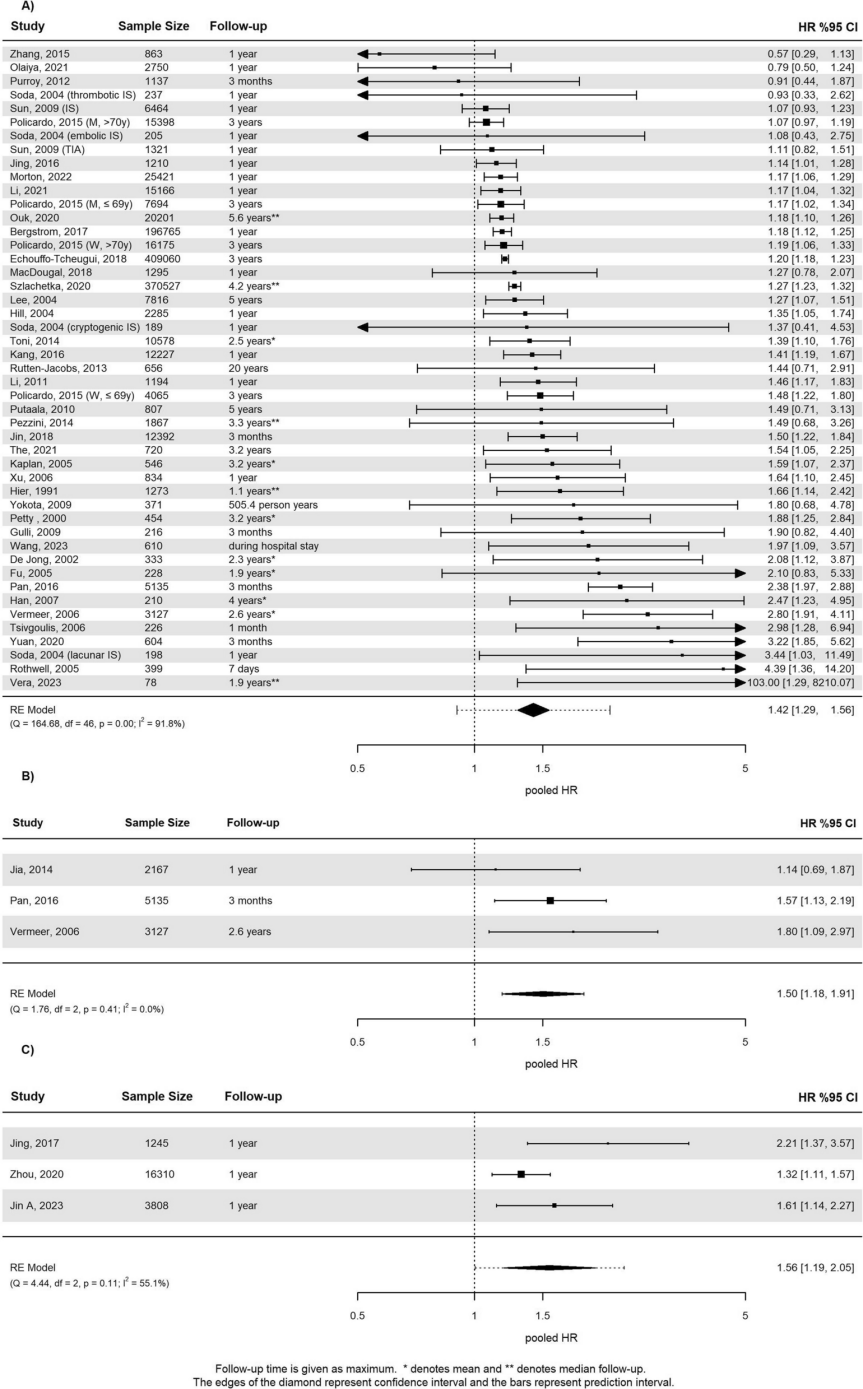
**a** Forest plot for the meta-analysis of studies that reported the association of diabetes with recurrent stroke. **b** Forest plot for the meta-analysis of studies that reported the association of prediabetes with recurrent stroke. **c** Forest plot for the meta-analysis of studies that reported the association of insulin resistance with recurrent stroke

Sixty-three studies reported recurrent stroke outcome data in patients with DM, see supplementary Table 5. Follow-up duration ranged from discharge from hospital to a mean follow-up time of 12.3 years. Studies encompassing the same population were excluded from the analysis. Finally, 40 studies reporting HR and 12 studies reporting OR were eligible for analysis, respectively. The pHR was 1.42 (95% CI 1.29 to 1.56, I^2^ = 92%; Fig. 3A) involving 1.161.527 patients. There was evidence for possible publication bias (supplementary Fig. 3). Studies that reported OR involving 47,629 patients showed a similar increase of risk (pOR 1.33, 95% CI 1.13 to 1.56, I^2^ = 48%; supplementary Fig. 4). Follow-up duration was not a statistically significant moderator for the outcome (p = 0.40). Neither the type of baseline event (IS or TIA), nor previous stroke was a statistically significant moderator (p = 0.08 and p = 0.90, respectively, see supplementary Fig. 5) in subgroup analyses. Baujat plots revealed that the studies contributing most to heterogeneity had a design of post-hoc analysis of randomized trials. Meta-analysis of absolute risks extracted from 23 studies resulted in 13% (95% CI 10% to 16%) for patients with diabetes vs. 9% (95% CI 6% to 11%) without, within a follow-up period of more than a year.

Patients with prediabetes had an increased risk for recurrent stroke compared to patients with normoglycemia (pHR in 10,429 patients 1.50, 95% CI 1.18 to 1.91, I^2^ = 0%, see Fig. 3B). This was also the case in terms of absolute risk 10% (95% CI 8% to 12%) and 7% (95% CI 7% to 8%), respectively. Of five studies eligible for IR, only three could be included in the meta-analysis, because multiple studies were conducted in the same cohort. The pHR for recurrent stroke associated with IR in 21,363 patients was 1.56, 95% CI 1.19 to 2.05, I^2^ = 55% (Fig. 3C). Absolute risks associated with IR during 10.4 months follow-up was 10% (95% CI 5% to 15%) vs. 7% (95% CI 6% to 7%) in patients without increased IR.

#### Cardiac events

All studies eligible for cardiac events comprised DM as the exposure, see supplementary Table 6. The shortest follow-up time was three months, all other studies followed patients for at least one year. One study that investigated new DM during follow-up was not included in the meta-analysis [26]. Presence of DM was associated with an increased risk of cardiac events with a pHR of 1.55 (95% CI 1.50 to 1.61, I^2^  = 0%) involving 443,863 patients. The pOR of two studies with 839,029 patients was 1.47 (95% CI 0.48 to 4.44), I^2^ = 89% (supplementary Fig. 6). Meta-analysis of three studies reporting data revealed an absolute risk of 5% (95% CI − 1% to 11%) in patients with DM and 3% (95% CI 0% to 6%) without DM. One study that investigated prediabetes reported a HR of 2.0 (95% CI 1.30 to 3.20) for cardiac events. No study reported IR as an exposure.

### Association between IGM and mortality

#### Cardiovascular mortality

Five studies reported data of cardiovascular mortality in patients with DM (supplementary Table 7). Meta-analysis involving 127,445 patients showed a statistically significant association between DM and cardiovascular mortality (pHR 1.65, 95% CI 1.41 to 1.93, I^2^ = 50%, see supplementary Fig. 7). Pooling available data of absolute risks from three studies, resulted in a pooled risk of 18% (95% CI −10% to 47%) in patients with DM vs. 16% (95% CI −9% to 41%) in patients without DM, during 1 year of follow-up.

#### All-cause mortality

**Fig. 4:**
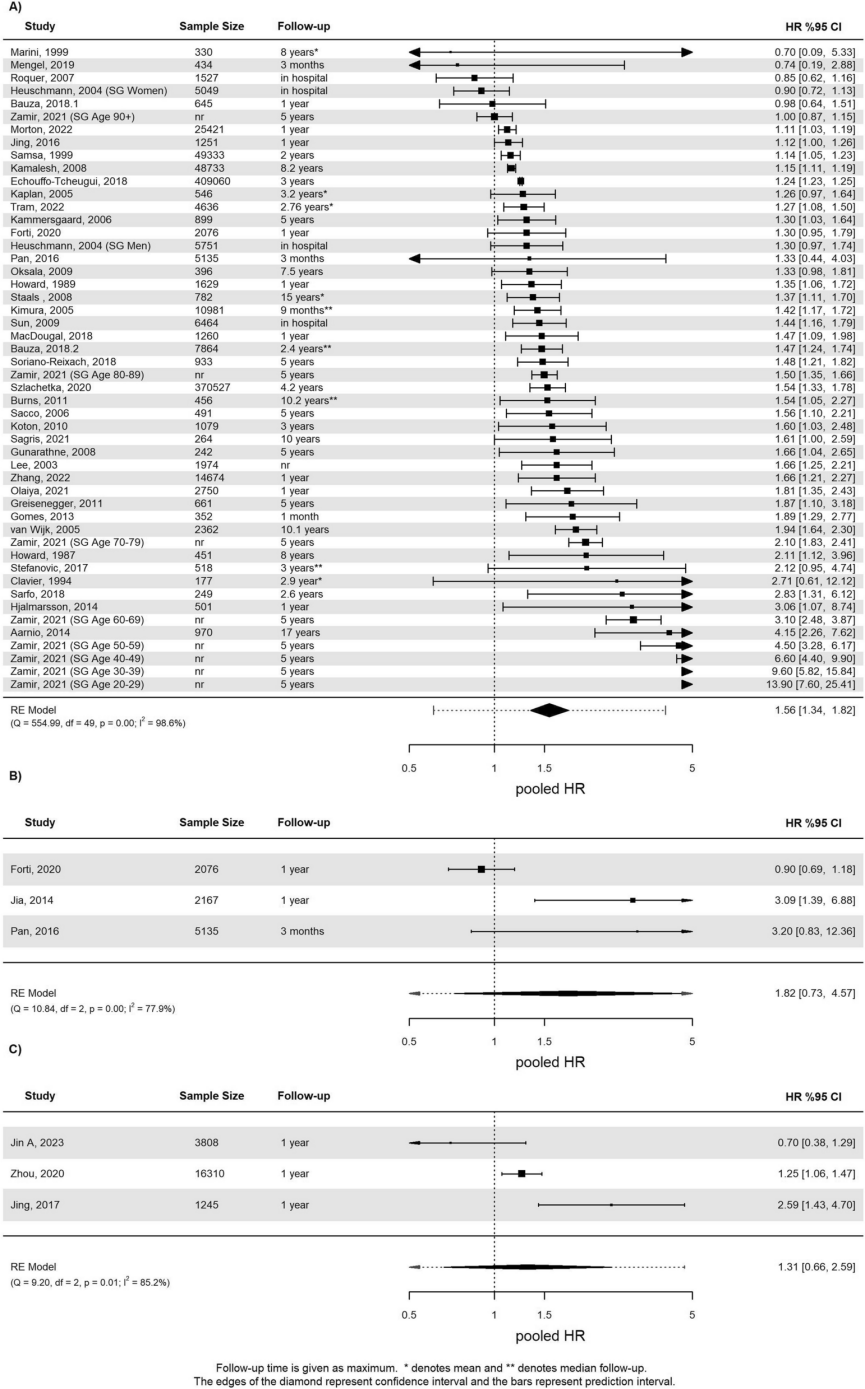
**a** Forest plot for the meta-analysis of studies that reported the association of diabetes with all-cause mortality. **b** Forest plot for the meta-analysis of studies that reported the association of prediabetes with all-cause mortality. **c** Forest plot for the meta-analysis of studies that reported the association of insulin resistance with all-cause mortality

Ninety-four studies investigated associations between all-cause mortality and DM, see supplementary Table 8. Studies that included patients from the same population were excluded from the analysis (n = 10). Presence of DM was associated with an increased risk for all-cause mortality (pHR 1.56, 95% CI 1.34 to 1.82, I^2^ = 99%, see Fig. 4A) summarizing 42 studies including 1.031.472 patients. Subgroup analyses based on follow-up duration resulted in a pHR of 1.10 (95% CI 0.72 to1.68) during hospitalization (n = 3 studies), pHR of 1.35 (95% CI 1.18 to 1.56) up to one year (n = 12 studies), and pHR of 1.74 (95% CI 1.40 to 2.17) longer than one year (n = 27 studies). However, follow-up duration was not revealed as a statistically significant moderator (p = 0.15, see supplementary Fig. 8). The Galbraith plot revealed the most influential studies to be the subgroups of the study from Zamir *et al.* (supplementary Fig. 9). The meta-analysis of forty-two studies involving 3.290.353 patients reporting OR showed a risk estimate of 1.30 (95% CI 1.21 to 1.41, see supplementary Fig. 10). Subgroup analyses based on first-ever vs. recurrent event at baseline and the type of ischemic event revealed no statistically significant difference between groups. Funnel plots suggested existence of publication bias (supplementary Fig. 11). During a mean follow-up of 1.8 months, the absolute risk of all-cause mortality was 23% (95% CI 14% to 31%) for patients with DM vs. 17% (95% CI 11% to 23%) without DM.

Six studies were eligible for prediabetes and all-cause mortality (3 HR, 3 OR). Prediabetes was not statistically significantly associated with an increased risk for mortality after IS (pHR 1.82, 95% CI 0.73 to 4.57, I^2^ = 78% in 9,378 patients, and pOR 1.37, 95% CI 0.54 to 3.43, I^2^ = 71% in 1,969 patients, see Fig. 4B & supplementary Fig. 12). Meta-analysis of absolute risks during a mean follow-up of seven months was 8% (95% CI 2% to 15%) for patients with prediabetes vs. 9% (95% CI 0% to 18%) with normoglycemia.

Nine studies reported IR as an exposure. The meta-analyses could not demonstrate an association between increased IR and mortality (pHR 1.31, 95% CI 0.66 to 2.59, I^2^ = 85%, including 21,363 patients across three studies and pOR 1.05, 95% CI 0.76 to 1.45, I^2^ = 16%, including 6,434 patients across 2 studies). Absolute risks were 6% (95% CI -1% to 12%) for patients with increased IR and 4% (95% CI 2% to 6%) without.

### Sensitivity analyses with crude odds ratios

Sensitivity analyses using unadjusted odds ratios, to accommodate the variation in adjustment factors used across studies, revealed similar risk estimates, though often slightly higher than the respective adjusted pooled outcomes (supplementary Fig. 13 and 14).

### Antidiabetic therapy and recurrent vascular events

Nine observational studies investigated the association between antidiabetic therapies and cardiovascular events after an IS or TIA in the preceding three months, see Table 2. The drug classes investigated were metformin, sulfonylurea, thiazolidinedione, and incretin-mimetics. We did not identify and studies with SGLT-2 Inhibitors or alfa glucosidase inhibitors. Due to the differences in the exposure and comparator groups, we did not perform a meta-analysis. Studies showed a risk reduction for recurrent stroke, mortality and composite vascular events associated with the use of pioglitazone and lobeglitazone as well as a lower risk of mortality associated with metformin use [29–32]. There were no clear benefits in terms of decreased risk of cardiovascular events associated with sulfonylurea or incretin-mimetics [33–37].

**Table 2:** Eligible studies for the exposure antidiabetic therapy

Study	Design	Population	Intervention/Exposure versus (vs.) Comparator	Outcome OR/HR (95% Confidence Interval)
**Metformin**				
Tu, 2022 [32]	Prospective Cohort Study	First-ever IS with DM	Metformin vs. No-Metformin use	1-year mortality OR 0.65 (0.48–0.84)
**Sulfonylurea**				
Tsivgoulis, 2017 [37]	Retrospective cohort study	Acute IS treated with Thrombolysis and DM	Pretreatment with sulfonylurea vs. no pretreatment with sulfonylurea	In hospital mortality OR 3.81 (0.90–16.17)
Favilla, 2011 [36]	Prospective cohort	IS with DM	Pre-stroke Sulphonylurea use vs non-use	90-day mortality OR: 1.30 (0.92–1.86)
Horsdal, 2012 [38]	Cohort Study	First-ever IS with DM	Use of metformin, insulin, or no therapy vs. Sulphonylurea (Reference)	1-year mortality • Metformin HR: 0.91 (0.66–1.26) • Insulin HR: 0.88 (0.66–1.19) • No therapy HR: 0.81 (0.66–1.01)
**Thiazolidinedione**				
Yoo, 2023 [29]	Nested case–control	DM and admitted for IS	Use of Lobeglitazone vs. no thiazolidinedione	• Stroke OR: 0.85 (0.68–1.08) • MI OR: 0.70 (0.33–1.47) • All-cause death OR: 0.58 (0.39–0.86) • Composite events: OR 0.74 (0.61–0.90)
			Use of Pioglitazone vs. no thiazolidinedione	• Stroke OR: 0.78 (0.69–0.89) • MI OR: 0.71 (0.47–1.08) • All-cause death OR: 0.60 (0.49–0.72) • Composite events: OR 0.71 (0.64–0.78)
Woo, 2019 [30]	Nested case–control	DM and admitted for IS	Pioglitazone use vs. treatment with insulin, and oral antidiabetic medications	• Stroke OR: 0.70 (0.31–1.61) • MI OR: not applicable (0 events with intervention) • All-cause death OR: 0.27 (0.09–0.79)
Morgan, 2018 [31]	Case–control	DM and admitted for IS	Pioglitazone initiation (case) vs. remaining in the same previous therapy regime (control)	Recurrent stroke HR: 0.76 (0.62–0.93)
**Incretin-Mimetics**				
Chen, 2015 [33]	Cohort study	DM and admitted for IS	Receivement of Sitagliptin vs No-Sitagliptin	Follow-up: max 2.8 years • IS HR: 0.95 (0.78–1.16) • MI HR: 0.90 (0.41–1.97) • CVD HR: 1.25 (0.86.-1.83) • Combined: HR 1.02 (0.85–1.21)
Chen, 2020 [34]	Cohort study	DM and admitted for IS	Receivement of Vildagliptin vs. No-Vildagliptin	Follow-up: max 2.8 y Combined CVD, Stroke, MI: HR 0.91 (0.71–1.16)
Li, 2018 [35]	Retrospective cohort study	DM and admitted for IS	Linagliptin vs non-exposure to incretin-based therapy	• Non-fatal ischemic stroke HR: 0.49 (0.21–1.12) • Non-fatal MI HR: not applicable (no events in either group) • CVD HR: 0.77 (0.24–2.46) • All-cause Death: 0.88 (0.40–1.92)

*CVD* Cardiovascular Death; *IS* Ischemic Stroke; *DM* Diabetes Mellitus; *HR* Hazard Ratio; *MI* Myocardial Infarction; *OR* Odds Ratio

## Discussion

In this systematic review and meta-analysis, we provide a comprehensive and up-to-date summary of previous studies investigating the association between IGM and residual cardiovascular risk following IS and TIA. To our knowledge, this is the first meta-analysis to investigate the risk of composite vascular events associated with IGM as well as the risk of mortality associated with DM in this population. The results of the presented meta-analysis indicate that (1) patients with DM have an approximately 1.6-fold (60%) increased risk of both death and recurrent vascular events after IS and TIA, (2) the risk of recurrent vascular events after stroke is already increased in the prediabetic stage and appears just as high as in patients with DM, and (3) presence of IR is associated with recurrent stroke risk. In contrast, this meta-analysis was unable to demonstrate an increased mortality risk after stroke associated with prediabetes or IR. Overall, there were significantly fewer eligible studies on prediabetes and IR compared to DM (Table 1).

DM is a well-known risk factor for cardiovascular disease. The results of our study confirm a robust association between DM and risk of composite recurrent vascular events after IS and TIA. We could confirm the risk of recurrent stroke associated with DM that was previously reported in a meta-analysis by Zhang *et al*. [14] The risk of mortality in patients with DM is observed to be 56% higher compared to patients without DM. Although mortality risk estimates were greater for diabetic patients with increasing mean follow-up durations of studies, we could not observe a statistically significant interaction between mortality risk and follow-up duration. This could be due to the fact that there were only a few studies with short-term follow-up in studies that reported HR (supplementary Fig. 8) and only a few studies with long-term follow-up in studies that reported OR (supplementary Fig. 10). Still, inferring from this finding, DM likely remains a relevant risk factor over time and an important target for secondary prevention strategies, given the high prevalence of DM in this population [6].

Our analyses demonstrated a positive relationship between prediabetes and recurrent vascular events as well as between IR and stroke recurrence. However, there was no association detected between the two conditions and mortality. This difference could have several reasons: First, patients with prediabetes or IR are less likely to have been exposed to deleterious effects of a dysregulated glucose metabolism for a longer time, compared to patients with DM. Second, the shorter follow-up duration of studies investigating prediabetes and IR generally limits the probability to detect difference in mortality risk. The risk associated with prediabetes and recurrent stroke is in line with a previous meta-analysis conducted by Pan *et al*. in 2019 [15]. Despite substantial methodological differences such as avoiding pooling ORs and HRs together and excluding studies with hemorrhagic stroke in our study, also having identified two more studies, similar to Pan *et al*., we also could not demonstrate a relationship between prediabetes and mortality.

Contrary to DM, prediabetes has rather recently been regarded as a cardiovascular risk factor [39]. The meta-analysis conducted by Cai *et al.* showed a risk increase in all-cause mortality and vascular events associated with prediabetes in population-based cohorts as well as in patients with previous atherosclerotic disease [40]. Further, a recent analysis of the UK Biobank cohort including more than 400 thousand individuals confirmed the excess risk for any cardiovascular disease in patients with IGM compared to normoglycemia [41]. The risk was higher for DM than for prediabetes. Still, after accounting for obesity and use of antihypertensive and statins both risks were attenuated, lending support to the modifiability of the excess risk. Together with these previous findings, our results strongly support considering prediabetes as a continuous entity with DM on the spectrum of IGM, with a relevant increase in cardiovascular and mortality risk. 

There was a statistically significant association between increased IR and stroke recurrence. However, it should be noted that, there were only three studies eligible for the analysis and the parameters used to define an increased IR as well as the timing of measurement after stroke (7 days and 14 days) was heterogeneous between studies. IR can be increased during the acute phase of the stroke due to the stress reaction and show changes during this time [42]. The increased relative risk for recurrent stroke observed in patients with IR compared to patients without IR was higher than the relative risk in diabetics compared to non-diabetics. This might be explained by the differences in the patient groups. Patients with DM are more likely to receive antidiabetic treatment and have a higher risk of dying before suffering a recurrent stroke. Another difference could be in the comparator groups, namely that the patients without IR could be generally healthier than patients without DM.

Despite the association between increased IR and stroke recurrence, we could not identify many studies with other cardiovascular outcomes. Furthermore, we encountered different parameters and criteria to define IR across studies. Thus, prognostic value of increased IR in terms of composite cardiovascular risk as well as the best biomarker to predict the said risk remains speculative in patients with IS or TIA. Further research is needed to investigate this conundrum.

We observed a significant research gap in the number of large studies with congruent definitions of prediabetes and IR. Uncertainty remains about the different diagnostic criteria for both prediabetes and IR [24, 25, 43, 44], leading to the lack of adequate implementation of preventive strategies [45]. As the prevalence of prediabetes expected to rise, the whole spectrum of IGM rather than DM alone is assumed to gain more significance in terms of primary and secondary stroke prevention [46]. Consistent diagnostic criteria would facilitate a reliable data synthesis and the development of prevention strategies.

Until the advent of the GLP1 and SGLT2 therapies, no antidiabetic therapy has improved cardiovascular risk or death despite improvements in glucose control [47]. Both classes of drugs revolutionized the field after randomized controlled trials showed cardiovascular risk reduction in patients with DM [48–51]. However, until now, it is unclear if these drugs are equally effective at reducing cardiovascular risk in patients with IS [33, 34, 52]. As our systematic review indicates, to date, only few studies exist that investigated the effectiveness of antidiabetic therapy in preventing recurrent vascular events after an acute or subacute IS. Even though the promising results related to pioglitazone use in patients with IR from the IRIS trial unfortunately faced a limitation due to side effects [18], recent cohort studies shown beneficial effects associated with thiazolidinediones [29, 30]. Clinical trial investigating secondary stroke prevention in patients with prediabetes are yet to been undertaken.

## Strengths and limitations

The most important strength of our study lies in the comprehensiveness, encompassing over 10.000 records and having included more than seven million patients over all exposures and outcomes. This enabled us to investigate all three entities of IGM together. Another strength constitutes the methodology. We included studies with both outcome measures HR and OR, which led us to identify more studies. We also used multi-level meta-analysis to account for multiple subgroups of the same cohorts and used meta-regression to account for moderators.

There are limitations to this study. Firstly, as in every meta-analysis, the quality of synthesized evidence depends on the quality of evidence of the individual studies. We assessed the risk of bias of the included studies and could not identify an influence of studies with high risk of bias on the effect estimates. Secondly, we encountered high heterogeneity between studies. As this systematic review included observational studies, the high variability across study populations and diagnostic criteria used was expected. Further, the fact that studies used different adjustment factors in their multivariable analyses most likely contributed substantially to the high heterogeneity. To alleviate the difference in the adjustment factors, we have conducted sensitivity analyses. Both crude odds ratios and absolute risks indicated a similar change of risk estimates to the per protocol analyses, strengthening our primary results. Another factor contributing to heterogeneity could be methodological differences between studies, such as how competing events were treated. This could not be taken into consideration when determining eligibility, since the information was mostly not available. Finally, severity and duration of DM could not be taken into consideration.

## Conclusion

Different types of IGM are associated with increased cardiovascular risk and mortality after IS and TIA. The entities of IGM should be considered as a continuous spectrum with increased cardiovascular risk that represent an important target for early cardiovascular prevention programs.

### Supplementary Information

Additional file1 (DOCX 3212 kb)

## Data Availability

The extracted data from the involved studies in this systematic review have been made available in supplementary material.

## Abbreviations

IS: Ischemic stroke
DM: Diabetes mellitus
IGM: Impaired glucose metabolism
IR: Insulin resistance
HR: Hazard ratios
OR: Odds ratios
CI: Confidence interval
pHR: Pooled HR
pOR: Pooled OR
