# Supplementary material for: Impaired glucose metabolism and the risk of vascular events and mortality after ischemic stroke: A systematic review and meta-analysis
Source: Cardiovasc Diabetol. 2024 Aug 31;23:323. doi: 10.1186/s12933-024-02413-w (PMC11366144; doi:10.1186/s12933-024-02413-w)
Supplement: Supplementary file 1 — Additional file1 (DOCX 3212 kb) [file 12933_2024_2413_MOESM1_ESM.docx]

# Online supplementary material to “Impaired glucose metabolism and the risk of vascular events and mortality after ischemic stroke: a systematic review and meta-analysis”

# Contents

Supplementary Methods

Supplementary table 1: Inclusion and exclusion criteria

Supplementary table 2: The definitions of population, exposure, and outcomes of the studies included in the review

Supplementary table 3: Eligible studies for the outcome composite vascular events

Supplementary table 4: Meta-analysis of absolute risk estimates for all outcomes Supplementary table 5: Eligible studies for the outcome recurrent stroke

Supplementary table 6: Eligible studies for the outcome cardiac events

Supplementary table 7: Eligible studies for the outcome cardiovascular mortality

Supplementary table 8: Eligible studies for the outcome all-cause mortality

Supplementary figure 1: Risk of bias assessment of the studies included in the review

Supplementary figure 2: Funnel plot for the outcome composite vascular events and diabetes mellitus

Supplementary figure 3: Funnel plot for the outcome recurrent stroke and diabetes mellitus

Supplementary figure 4: Forest plot for the outcome recurrent stroke and diabetes mellitus in odds ratios

Supplementary figure 5: Forest plot for subgroup analysis of the outcome recurrent stroke and diabetes mellitus

Supplementary figure 6: Forest plot for the outcome cardiac events and diabetes mellitus

Supplementary figure 7: Forest plot for the outcome cardiovascular mortality and diabetes mellitus

Supplementary figure 8: Forest plot for subgroup analysis of the outcome all-cause mortality and diabetes mellitus

Supplementary figure 9: Galbraith plot for the outcome all-cause mortality and exposure diabetes mellitus

Supplementary figure 10: Forest plot for the outcome all-cause mortality and diabetes mellitus in odds ratios

Supplementary figure 11: Funnel plots for the outcome all-cause mortality diabetes mellitus

Supplementary figure 12: Forest plot for the outcome all-cause mortality and prediabetes

Supplementary Figure 13: Adjustment factors of the studies included in the review

Supplementary figure 14: Crude odds ratios for all outcomes

Supplementary references

# Methods

## Search strategies of the systematic review

| **Medline accessed via Pubmed** |
| --- |
| (("stroke"[MeSH Terms]) OR ("ischemic attack, transient"[MeSH Terms])) AND ("diabetes mellitus"[MeSH Terms] OR ("insulin resistance"[MeSH Terms]))  Filters applied: Human and English language |
| **Cochrane Library** |
| (MeSH descriptor: [Stroke] explode all trees OR MeSH descriptor: [Ischemic Attack, Transient] explode all trees) AND (MeSH descriptor: [Diabetes Mellitus] explode all trees OR MeSH descriptor: [Insulin Resistance] explode all trees) |
| **Embase accessed via Ovid** |
| #1: exp *diabetes mellitus/ or exp diabetic complication/ or exp impaired glucose tolerance/ or exp insulin dependent diabetes mellitus/ or exp non insulin dependent diabetes mellitus/  #2: exp insulin resistance/  #3: 1 or 2  #4: exp brain ischemia/ or exp transient ischemic attack/  #5: 3 and 4  #6: limit 5 to (human and English language)  #7: (review or comment or editorial).pt.  #8: 6 not 7 |

## Data extraction

Data were extracted from text and tables of reports using a standardized form with the online tool “Numbat” (Carlisle BG. Numbat {S}ystematic {R}eview {M}anager. Published online 2014) and “WebPlotDigitizer” (Rohatgi A. WebPlotDigitizer Version 4.5. https://automeris.io/WebPlotDigitizer) was used to extract data from graphics when necessary. The following data were extracted: Author, title, journal, publication year, definition of the population and exposure, characteristics of the index event (first-ever or recurring; IS, TIA or both), key inclusion and exclusion criteria, follow-up duration, sample size, demographics, outcome measures with the corresponding 95% confidence intervals (95% CI), confounding factors used for adjustment, and the unadjusted outcome measure.

## Study selection and data synthesis

We included risk estimates from the models with the most adjusting factors. If multiple studies reported data from the same cohort of patients, we included the study with the largest sample size and longest follow-up. To avoid multiple representation of a patient in the analysis, only one subgroup was chosen, in case same patients were included in the comparison group for different subgroups of exposures.

Risk ratios were interpreted as HR or OR if they were calculated using cox regression and logistic regression, respectively. Unadjusted odds ratios were calculated for studies that reported necessary data (event rates or number of events for each exposure and outcome).

## Assessment of risk of bias

The Study Quality Assessment Tool of National Heart, Lung, and Blood Institute consists of 14 criteria. Quality of evidence was rated “good” if there was no indication of selection bias (Items 3 and 4); the populations, exposures and outcomes were defined clearly and applied consistently throughout study participants (Items 2, 9, and 11); and there was no substantial data loss due to loss to follow up (Item 13). If one or two of these criteria were not fulfilled, the study was rated as “fair” and “poor”, respectively.

# Supplementary table 1: Inclusion and exclusion criteria

| **Category** | **Selection Criteria** |
| --- | --- |
| **Population** | **Inclusion Criteria**   - Patients with IS or TIA defined according to WHO definitions. - Subacute and chronic stroke will be distinguished based on the cut-off of 3 months from stroke onset.   **Exclusion Criteria**   - Studies that did not differ between ischemic and hemorrhagic stroke or only reported data for all stroke without further differentiation of type of stroke - If it cannot be excluded that stroke types other than IS have been included in the study cohort - Studies that only enrolled subjects based on presence of specific conditions (e.g., chronic kidney disease, heart failure) - Studies with animals |
| **Exposure and comparator** | **Inclusion Criteria**   - Diabetes mellitus regardless of type; prediabetes (defined either as impaired glucose tolerance, impaired fasting glucose, or both); and insulin resistance with the comparison groups consisting of patients without the respective condition. - (only for the research question regarding antidiabetic therapy) antidiabetic agents as intervention compared to placebo or another antidiabetic agent |
| **Outcome** | **Inclusion Criteria**   - Cardiovascular events including stroke, myocardial infarction, and mortality, regardless of cause (e.g., cardiovascular, all-cause). - Composite outcomes defined as combinations of these outcomes. - Outcomes reported as HR, risk ratios or OR that are adjusted for covariates using the model with the most adjustment factors.   **Exclusion Criteria**   - Studies that report the risk of outcome without adjustment for at least one confounder (crude risk). |
| **Study design** | **Inclusion Criteria**   - Prospective or retrospective observational studies or case-control studies regardless of length of follow-up - Case-control studies when matching was made at least based on age or sex |
| **Other** | **Inclusion Criteria**   - Publications in English language - Review articles, meta-analyses, commentaries, case reports, trial protocols, conference abstracts, guidelines, editorials, letters, book chapters, mendelian randomization studies |

# Supplementary table 2: The definitions of population, exposure, and outcomes of the studies included in the review

| **Study**  **(First author, year)** | **Population** | **Exposures** | | | | **Outcomes** | | | | | |
| --- | --- | --- | --- | --- | --- | --- | --- | --- | --- | --- | --- |
|  | **Source of the Population** | **Diabetes** | **Prediabetes** | **Insulin Resistance** | **Definitions of the exposures (in the same order)** | **Recurrent Stroke** | **Cardiac Events** | **All-cause Mortality** | **Cardiovascular Mortality** | **Composite Outcome** | **Definitions of the outcomes (in the same order)** |
| Lu, 2023^1^ | Third China National Stroke Registry, China |  |  | x | Estimated glucose disposal rate | x |  |  |  | x | • Recurrent IS or HS  • IS, HS, MI, cardiovascular mortality |
| Jin, 2023^2^ | Third China National Stroke Registry, China |  |  | x | HOMA-IR Index | X |  | X |  | x | • Recurrent IS or HS  • All-cause Mortality  • IS, HS, MI, cardiovascular mortality |
| Tram, 2023^3^ | Shiga Stroke and Heart Attack Registry, Japan | X |  |  | Poor control of Diabetes: History of Diabetes and HbA1c >6.5% |  |  | x |  |  | All-cause Mortality |
| Vera, 2023^4^ | Single-center, Spain | x |  |  | FPG ≥126 mg/dL, 2-hour OGTT≥ 200 mg/dL, or HbA1c >6.5% | x |  |  |  |  | • Recurrent stroke |
| Wang, 2023^5^ | Multi-center, China | X |  |  | History of DM | x |  |  |  |  | • Recurrent IS, HS or TIA |
| Cao, 2022^6^ | Third China National Stroke Registry, China | x |  |  | History of DM | X |  | x |  |  | • Recurrent IS or HS, SAH  • All-cause Mortality |
| Genceviciute, 2022^7^ | Bernese Stroke Centre registry, Switzerland | x |  |  | HbA1c >6.5% or treatment for DM | x |  | x |  |  | • Recurrent IS or HS, SAH  • All-cause Mortality |
| Guo, 2022^8^ | CHANCE Trial, China | x |  |  | History of DM | x |  |  |  | x | • Recurrent IS or HS  • IS, HS, MI, cardiovascular mortality |
| Hu, 2022^9^ | Chinese Stroke Center Alliace, China | x |  |  | History of DM, HbA1c >6.5% or treatment for DM |  | x | x |  |  | • MI  • All-cause Mortality |
| Liu, 2022^10^ | Second China National Stroke Registry, China |  |  | x | Triglyceride Glucose Index | x |  | x |  |  | • Recurrent IS  • All-cause Mortality |
| Morton, 2022^11^ | Victorian Admitted Episodes Dataset, Australia | x |  |  | Diagnosis of T2DM and use of treatment for DM acquired from database | x |  | x |  |  | • Recurrent IS  • All-cause Mortality |
| Yang, 2022^12^ | ACROSS-China Study, China |  |  | x | Triglyceride Glucose Index and HOMA-IR Index |  |  | x |  |  | All-cause Mortality |
| Zhang, 2022^13^ | Third ChinaNational Stroke Registry, China | x |  |  | History of DM or treatment for DM | x |  | x |  |  | • Recurrent IS or HS, SAH  • All-cause Mortality |
| Kim, 2021^14^ | Clinical Research Collaboration for Stroke in Korea registry, Korea | x | x |  | • HbA1c >6.4% or history of DM  • HbA1c 5.7-6.4% |  |  | x |  |  | All-cause Mortality |
| Li, 2021^15^ | First, Second and Third ChinaNational Stroke Registry, China | x |  |  | History of DM or treatment for DM | x |  |  |  |  | Recurrent IS or HS, SAH |
| Lopez-de-Anders, 2021^16^ | Spanish National Hospital Discharge Database, Spain | x |  |  | DM Diagnosis acquired from database |  |  | x |  |  | All-cause Mortality |
| MacIntosh, 2021^17^ | Ontario Stroke Registry, Canada | x |  |  | DM Diagnosis acquired from database | x |  | x |  |  | • Readmission for stroke or TIA  • All-cause Mortality |
| Olaiya, 2021^18^ | The Australian Stroke Clinical Registry, Australia | x |  |  | DM Diagnosis acquired from database | x |  | x | x |  | • Readmission for IS, HS  • All-cause Mortality  • Cardiovascular Mortality |
| Sagris, 2021^19^ | Athens Stroke, Registry, Greece | x |  |  | nr |  |  | x |  | x | • All-cause Mortality  • MI, cardiac death, aortic aneurysm rupture and recurrent stroke |
| The, 2021^20^ | Single center, Singapore | x |  |  | • FPG ≥126 mg/dL, 2-hour OGTT≥ 200 mg/dL, or HbA1c >6.5% | x | x |  |  | x | • Fatal or nonfatal stroke, TIA  • Fatal or nonfatal MI, unstable Angina pectoris  • Stroke, TIA, MI, angina pectoris |
| Zamir, 2021^21^ | Israeli National Stroke Registry, Israel | x |  |  | History of DM or treatment for DM |  |  | x |  |  | All-cause Mortality |
| Fang, 2020^22^ | Thrombolysis Implementation and Monitor of Acute Ischemic Stroke in China Study, China | x |  |  | History of DM or treatment for DM |  |  | x |  |  | All-cause Mortality |
| Forti, 2020^23^ | Single-center, Italy | x | x |  | • History of DM, treatment for DM or HbA1c >6.5% • HbA1c 5.7-6.4% |  |  | x |  |  | All-cause Mortality |
| Ouk, 2020^24^ | Ontario Stroke Registry, Canada | x |  |  | DM (with and without comorbid depression). DM Diagnosis acquired from database | x |  | x |  |  | • Readmission for stroke or TIA • All-cause Mortality |
| Szlachetka, 2020^25^ | Universal Coverage Health Security Insurance Scheme Database, Thailand | x |  |  | Diagnosis of T1DM and T2DM acquired from database | x |  | x |  | x | • Recurrent IS • All-cause Mortality • MI, cardiac arrest, and recurrent stroke |
| Yuan, 2020^26^ | Nanjing Stroke Registry Program, China | x |  |  | History of DM or FPG ≥126 mg/dL | x |  |  |  |  | Fatal or nonfatal IS or HS |
| Zhou, 2020^27^ | China National Stroke Registry, China |  |  | x | Triglyceride Glucose Index | x |  | x |  |  | • IS, ICH, SAH • All-cause Mortality |
| Akhtar, 2019^28^ | Single-center, Qatar | x |  |  | • HbA1c >6.5% | x |  |  |  |  | Recurrent stroke |
| Choi, 2019^29^ | Single-center, South Korea | x |  |  | • HbA1c ≥ 6.5% |  |  | x |  |  | All-cause Mortality |
| de Oliveira, 2019^30^ | Single-center, Brasil | x |  |  | History of DM, treatment for DM or capillary glucose >200 mg/dL |  |  | x |  |  | All-cause Mortality |
| Mengel, 2019^31^ | PREDICT Study, Germany, Spain | x |  |  | History of DM, treatment for DM or new diagnosis during admission |  |  | x |  |  | All-cause Mortality |
| Panni, 2019^32^ | Multi-center, France | x |  |  | History of DM |  |  | x |  |  | All-cause Mortality |
| Tsivgoulis, 2019^33^ | Safe Implementation of Treatments in Stroke International Stroke Thrombolysis Registry | x |  |  | History of DM, or glucose >144 mg/dL |  |  | x |  |  | All-cause Mortality |
| Ago, 2018^34^ | Fukuoka Stroke Registry, Japan |  |  | x | HOMA-IR | x |  | x |  |  | • IS or HS • All-cause Mortality |
| Altavilla, 2018^35^ | Multi-center, Italy | x |  |  | Treatment for DM, FPG >126 mg/dL, postprandial glucose >200 mg/dL, or HbA1c >8.5% |  |  |  |  | x | Fatal and non-fatal stroke, recurrent TIA, fatal and non-fatal MI, and angina |
| Bauza, 2018^36^ | Interventional Management of Stroke III clinical trial, USA | x |  |  | nr |  |  | X |  |  | All-cause Mortality |
| Bauza, 2018^37^ | PRoFESS trial, international | x |  |  | DM Diagnosis acquired from database |  |  | x | x |  | • All-cause Mortality  • Death due to stroke, MI, hemorrhage excluding ICH, and other vascular causes |
| Chen, 2018^38^ | ENCHANTED trial, international | x |  |  | History of DM |  |  | x |  |  | All-cause Mortality |
| Echouffo-Tcheugui, 2018^39^ | Get With The Guidelines (GWTG)-Stroke Registry, USA | x |  |  | History of DM or new diagnosis during hospitalization | x | x | x |  | x | • Readmission with ICD-9 codes for IS • Admissions with AMI ICD-9 codes: 410.xx • All-cause Mortality • Mortality and Readmission for Cardiovascular disease |
| Jin, 2018^40^ | Nationwide Readmissions Database, USA | x |  |  | DM Diagnosis acquired from database | x | x |  |  |  | • Recurrent IS or HS • MI, congestive heart failure exacerbation, percutaneous coronary intervention, CABG, surgical or transcatheter aortic valve repair , cardiac ablation , cardioversion , cardiac catheterization, left atrial appendage closure , ICD placement , permanent pacemaker placement |
| MacDougal, 2018^41^ | Brain Attack Surveillance in Corpus Christi project, USA | x |  |  | History of DM acquired from patient records | x |  | x |  |  | • IS or HS • All-cause Mortality |
| Osei, 2018^42^ | Erasmus Stroke Study, Netherlands | x | x |  | • Treatment for DM  • IFG (FPG > 100 mg/dL) |  |  | x |  |  | All-cause Mortality |
| Read, 2018^43^ | National hospitalization register, Scotland | x |  |  | History of DM acquired from patient records |  |  | x |  |  | All-cause Mortality |
| Sarfo, 2018^44^ | Single-center, Ghana | x |  |  | History of DM, treatment for DM, HbA1c >6.5 % or FPG >126 mg/dL |  |  | x |  |  | All-cause Mortality |
| Soriano-Reixach, 2018^45^ | BASICMAR database, Spain | x |  |  | History of DM or treatment for DM |  |  | x | x | x | • All-cause Mortality • Cardiovascular death • Stroke, ICH, CAD, and cardiovascular death, previously unknown AF |
| Zhou, 2018^46^ | ACROSS-China Study, China |  |  | x | HOMA-IR, Insulin sensitivity index |  |  | x |  |  | All-cause Mortality |
| Bergström, 2017^47^ | Swedish Stroke Register, Sweden | x |  |  | DM Diagnosis acquired from database |  |  | x |  |  | All-cause Mortality |
| Chen, 2017^48^ | CHANCE Trial, China | x |  |  | DM with and without comorbid metabolic syndrome. DM defined as history of DM, treatment for DM or FPG ≥126 mg/dL | x |  |  |  | x | • New neurological deficit • IS, HS, MI, or vascular death |
| Corrao, 2017^49^ | National Health Service Registry, Italy | x |  |  | Treatment for DM |  |  |  |  | x | IS, HS, MI, or vascular death |
| Jing, 2017^50^ | ACROSS-China Study, China |  |  | x | HOMA-IR | x |  | x |  |  | • New neurological defcit, rehospitalization with IS or HS • All-cause Mortality |
| Pan, 2017^51^ | ACROSS-China Study, China |  |  | x | Matsuda Insulin Sensitivity Index, ISI Composite | x |  | x |  |  | • Aggravated primary neurologic deficit, new deficit or rehospitalization with IS or HS • All-cause Mortality |
| Pan, 2017^52^ | CHANCE Trial, China | x |  |  | History of DM | x |  |  |  |  | Recurrent IS or HS |
| Pirinen, 2017^53^ | Helsinki Young Stroke Registry, Finland | x |  |  | T1DM (Fasting glucose >126 mg/dL, glucose after 75g glucose ingestion >200 mg/dL and regular insulin use within 1 year of diagnosis) |  |  | x | x |  | • All-cause Mortality • Underlying, intermediate or immediate cause of death being a diagnosis from 390 to 459 in ICD-9 or from category I in ICD-10 |
| Stefanovic, 2017^54^ | Single-center, Serbia | x |  |  | nr |  |  | x |  |  | All-cause Mortality |
| Yoo, 2017^55^ | Single-center, South Korea | x |  |  | HbA1c ≥ 6.5% or FPG ≥126 mg or random glucose ≥ 200 mg/dL with hyperglycemia symptoms or treatment for DM |  |  |  |  | x | Cardiovascular mortality, IS, MI, unstable angina, urgent coronary revascularization, ACM, and elective coronary revascularization |
| Jing, 2016^50^ | ACROSS-China Study, China | x |  |  | • Newly diagnosed DM. Defined as FPG ≥126 mg/dL, 2-hour OGTT≥ 200 mg/dL, or HbA1c >6.5% | x |  | x |  |  | • Rehospitalization with IS or HS • All-cause Mortality |
| Kang, 2016^56^ | Clinical Research Center for Stroke Registry, South Korea | x |  |  | History of DM acquired from patient records | x | x |  |  | x | • Fatal or nonfatal IS or HS • Symptoms of myocardial ischemia, enzyme changes indicative of MI, and ECG changes suggesting new ischemia • Stroke, MI, and vascular death |
| Pan, 2016^57^ | CHANCE trial cohort | x | x |  | • History of DM, treatment for DM or FPG ≥126 mg/dL • IFG (FPG 110-126 mg/dL) | x | x | x |  | x | • IS or HS • MI • All-cause Mortality • Stroke, MI, or death from cardiovascular causes |
| Pan, 2016^58^ | China National Stroke Registry, China | x |  |  | • History of DM or treatment for DM | x |  | x |  |  | • IS, HS or SAH • All-cause Mortality |
| Wood, 2016^59^ | Universal Coverage Health Security Insurance Scheme Database, Thailand | x |  |  | T2DM, ICD Codes acquired from patient records |  |  | x |  |  | All-cause Mortality |
| Cruz-Herranz, 2015^60^ | Single-center, Spain | x |  |  | History of DM acquired from patient records | x |  |  |  |  | New cerebral infarct |
| Li, 2015^61^ | Single-center, China | x |  |  | History of DM and treatment for DM |  |  | x |  | x | • All-cause Mortality  • All new-onset fatal or non-fatal vascular events, including stroke, MI, and venous thrombosis 30 days after stroke |
| Lin, 2015^62^ | CHANCE Trial, China | x |  |  | History of DM |  |  |  |  | x | IS, MI, or mortality |
| Masrur, 2015^63^ | Get With the Guidelines Stroke Registry, USA | x |  |  | HbA1c ≥ 6.5% |  |  | x |  |  | All-cause Mortality |
| Muñoz-Rivas, 2015^64^ | Spanish National Hospital Database, Spain | x |  |  | T2DM, ICD Codes acquired from patient records |  |  | x |  |  | All-cause Mortality |
| Policardo, 2015^65^ | Regional Health System of Tuscany, Italy | x |  |  | History of DM and treatment for DM acquired from patient records | x |  |  |  |  | IS |
| Policardo, 2015^66^ | Hospital Discharge Database, Italy | x |  |  | History of DM and treatment for DM acquired from patient records |  |  | x |  |  | All-cause Mortality |
| Serena, 2015^67^ | The ARTICO study, Spain | x |  |  | History of DM |  |  |  |  | x | Recurrent stroke, ischemic heart disease, symptomatic peripheral arterial disease, vascular surgery and death of vascular or non-vascular origin |
| Wu, 2015^68^ | China National Stroke Registry, China | x |  |  | FPG ≥126 mg/dL, non-fasting glucose ≥ 200 mg/dL, treatment for DM or history of DM | x |  | x |  |  | • IS, ICH, SAH • All-cause Mortality |
| Zhang, 2015^69^ | Chinese Intra Cranial AtheroSclerosis Study, China | x |  |  | History of DM or diagnosis at discharge | x |  |  |  |  | IS (defined as a new neurological deficit >24 hours verified by cranial CT or MRI and) and TIA |
| Aarnio, 2014^70^ | Helsinki Young Stroke Registry, Finland | x |  |  | FPG ≥126 mg/dL. T1DM defined as insulin dependency with 1 year from diagnosis. |  |  | x |  |  | All-cause Mortality |
| Arnold, 2014^71^ | Single-center, Switzerland | x |  |  | History of DM and treatment for DM |  |  | x |  |  | All-cause Mortality |
| Erdur, 2014^72^ | Single-center, Germany | x |  |  | History of DM or FPG≥ 126 mg/dL |  |  | x |  |  | All-cause Mortality |
| Hjalmarsson, 2014^73^ | Single-center, Sweden | x |  |  | History of DM and treatment for DM |  |  | x |  |  | All-cause Mortality |
| Jia, 2014^74^ | ACROSS-China Study, China |  | x |  | IFG Fasting glucose 110-126 mg/dL and IGT (2hOGTT 140-200 mg/dL) | x |  | x |  |  | • Rehospitalization with IS or HS • All-cause Mortality |
| Pezzini, 2014^75^ | Italian Project on Stroke in Young Adults, Italy | x |  |  | History of DM, treatment for DM or FPG ≥126 mg/dL | x |  |  |  | x | • Sudden loss of global or focal cerebral function >24 hours  • IS, TIA, MI, and other arterial events |
| Roquer, 2014^76^ | Single-center, Spain | x | x |  | • History of DM, treatment for DM or HbA1c 6.5 % •HbA1c 5.7-6.4% and FPG< 126 mg/dL |  |  | x |  |  | All-cause Mortality |
| Rutten-Jacobs, 2014^77^ | FUTURE study, Netherlands | x | x |  | • Treatment for DM or FPG ≥126 mg/dL during follow-up • IFG (Fasting glucose 100-124 mg/dL) | x |  |  |  | x | • IS or HS • IS or HS, MI, and cardiovascular procedures (CABG, percutaneous transluminal coronary angioplasty, carotid endarterectomy or other peripheral arterial revascularization procedures) |
| Toni, 2014^78^ | PRoFESS trial, international | x |  |  | nr | x |  |  |  |  | Recurrent stroke of any type |
| Wu, 2014^79^ | ACROSS-China Study, China | x |  | x | • History of DM and treatment for DM  • HOMA-IR >1 |  |  | x |  |  | All-cause Mortality |
| Zhao, 2014^80^ | China National Stroke Registry, China | x |  |  | History of DM and treatment for DM |  |  | x |  |  | All-cause Mortality |
| Balti, 2013^81^ | Single-center, Cameroon |  |  | x | Glucose/Insulin Ratio (Insulin Sensitivity), QUICKI Index (Insulin Sensitivity), HOMA-IR |  |  | x | x |  | • All-cause Mortality • ICD 10 Codes I 00-99 |
| Gomes, 2013^82^ | STEPS Stroke study, Mozambique | x |  |  | History of DM and treatment for DM |  |  | x |  |  | All-cause Mortality |
| Hanchate, 2013^83^ | Discharge databases, multi-center, USA | x |  |  | ICD Codes acquired from patient records |  |  | x |  |  | All-cause Mortality |
| Rocco, 2013^84^ | Single-center, Germany | x |  |  | History of DM |  |  | x |  |  | All-cause Mortality |
| Rutten-Jacobs, 2013^77^ | FUTURE study, Netherlands | x |  |  | Random blood glucose > 200 mg/dL or 2 consecutive FPG ≥ 126 mg/dL | x | x |  |  | x | • Focal neurologic deficit persisting > 24 hours.  • Ischemic symptoms with ECG, biomarker pathologic evidence of infarction  • Fatal or nonfatal IS or HS, or MI, or cardiovascular procedures (CABG, PTCA, carotid endarterectomy, or other peripheral arterial revascularization procedures), |
| Wang, 2013^85^ | China National Stroke Registry, China | x |  |  | Treatment for DM or FPG ≥120 mg/dL | x |  |  |  |  | IS or HS |
| Wang, 2013^86^ | China National Stroke Registry, China | x |  |  | History of DM and treatment for DM | x |  | x |  |  | • Self-reported stroke re-hospitalization with IS, ICH or SAH. • All-cause Mortality |
| Fuentes, 2012^87^ | Madrid Stroke Network, Spain | x |  |  | History of DM and treatment for DM |  |  | x |  |  | All-cause Mortality |
| Purroy, 2012^88^ | Multi-center, Spain | x |  |  | History of FPG ≥126 mg/dL or treatment for DM | x |  |  |  |  | New stroke |
| Tsivgoulis, 2012^89^ | Single-center, Greece | x |  |  | FPG ≥126 mg/dL, 2-hour OGTT ≥ 200 mg/dL, HbA1c >6.5% or treatment for DM | x |  |  |  |  | Aggravated or new neurologic deficit |
| Andersen, 2011^90^ | Danish National Indicator Project, Denmark | x |  |  | History of DM or diagnosis during admission |  |  | x |  |  | All-cause Mortality |
| Burns, 2011^91^ | Rochester Epidemiology Project, USA | x |  |  | nr |  |  | x |  |  | All-cause Mortality |
| Greisenegger, 2011^92^ | Vienna Stroke Registry, Austria | x |  |  | History of DM, treatment for DM or FPG >126 mg/dl |  |  | x |  |  | All-cause Mortality |
| Hassan, 2011^93^ | Single-center, Malaysia | x |  |  | Treatment for DM or history of glucose >126 mg/dl |  |  | x |  |  | All-cause Mortality |
| Jia, 2011^94^ | China National Stroke Registry, China | x |  |  | History of DM, treatment for DM during hositalization | x |  |  |  |  | Rehospitalization with IS, HS or SAH |
| Li, 2011^95^ | Bureau of National Health Insurance Database, Taiwan | x |  |  | Diagnosis acquired from database | x |  |  |  |  | Readmission with ICD-9 433 or 434 |
| Winell, 2011^96^ | National Hospital Discharge Register, Finland | x |  |  | T2DM Diagnosis acquired from patient records |  |  |  | x |  | Underlying or the direct cause of death being ICD-9 codes 410–414, 798, 430–434, 436 and 438 and ICD-10 codes I20–I25, I46, R96, R98, I60–I66 and I69 (except for I63-6) |
| Ahmed, 2010^97^ | Safe Implementation of Treatments in Stroke International Stroke Thrombolysis Registry, International | x |  |  | History of DM |  |  | x |  |  | All-cause Mortality |
| Fonarow, 2010^98^ | Get With The Guidelines (GWTG)-Stroke Registry, USA | x |  |  | History of DM and treatment for DM |  |  | x |  |  | All-cause Mortality |
| Hassan, 2010^99^ | Single-center, Malaysia | x |  |  | Treatment for DM or history of blood glucose >126 mg/dl |  |  | x |  |  | All-cause Mortality |
| Koton, 2010^100^ | Multi-center, Israel | x |  |  | nr |  |  | x |  |  | All-cause Mortality |
| Meurer, 2010^101^ | Multi-center, USA | x |  |  | History of DM |  |  | x |  |  | All-cause Mortality |
| Nakajima, 2010^102^ | Single-center, Japan | x |  |  | nr | x |  |  |  |  | Recurrent stroke based on discharge records |
| Putaala, 2010^103^ | Single-center, Finland | x |  |  | T1DM, T2DM. Treatment for DM or FPG ≥ 126 mg/dL | x |  |  |  | x | • Fatal or nonfatal stroke • Nonfatal or fatal recurrent IS; and nonfatal or fatal MI, other arterial thrombotic event, or revascularization procedure |
| Putaala, 2010^104^ | Single-center, Finland | x |  |  | nr |  |  | x |  |  | All-cause Mortality |
| Reeves, 2010^105^ | Get With The Guidelines Stroke Registry, USA | x |  |  | History of DM and treatment for DM |  |  | x |  |  | All-cause Mortality |
| Smith, 2010^106^ | Get With The Guidelines Stroke Registry, USA | x |  |  | History of DM and treatment for DM |  |  | x |  |  | All-cause Mortality |
| Spengos, 2010^107^ | Single-center, Greece | x |  |  | Treatment for DM or FPG ≥120 mg/dl |  |  |  |  | x | RS, MI, unstable angina, aortic aneurysm rupture, peripheral embolism, new onset of HF and sudden death, with or without resuscitation |
| Wu, 2010^108^ | Chengdu Stroke Registry, China | x |  |  | Treatment for DM or FPG >126 mg/dl |  |  | x |  |  | All-cause Mortality |
| Busch, 2009^109^ | Single-center, Germany | x |  |  | History of DM, treatment for DM or FPG >126 mg/dl or non-fasting glucose > 126 mg/dL |  |  |  |  | x | Stroke, MI or death |
| Gulli, 2009^110^ | Single-center, UK | x |  |  | History of DM, FPG ≥ 126 mg/dL, two random glucose values of ≥200 mg/dL | x |  |  |  |  | RS confirmed with imaging |
| Oksala, 2009^111^ | The Helsinki Stroke Aging Memory cohort, Finland | x |  |  | Treatment for DM or FPG >126 mg/dl |  |  | x |  |  | All-cause Mortality |
| Putaala, 2009^112^ | Helsinki Young Stroke Registry, Finland | x |  |  | FPG ≥ 126 mg/dL |  |  | x |  |  | All-cause Mortality |
| Sun, 2009^113^ | National Healthcare Group Database, Singapore | x |  |  | History of DM or FPG ≥ 126 mg/dL | x |  | x |  |  | • Readmission with stroke recurrence • All-cause Mortality |
| Yokota, 2009^114^ | Single-center, Japan | x |  |  | Treatment for DM, FPG ≥126 mg/dL or random glucose >200 mg/dL | x |  |  |  |  | Fatal or nonfatal stroke |
| Gunarathne, 2008^115^ | West Birmingham Stroke Project Registry, UK | x |  |  | History of DM or FPG ≥ 140 mg/dL |  |  | x |  |  | All-cause Mortality |
| Kamalesh, 2008^116^ | Multi-center, USA | x |  |  | History of DM acquired from patient records |  |  | x |  |  | All-cause Mortality |
| Staals, 2008^117^ | Maastricht Stroke Registry, Netherlands | x |  |  | History of DM, FPG > 126 mg/dL or two values of postprandial glucose >200 mg/dL before or at least 3 days after stroke |  |  | x |  |  | All-cause Mortality |
| Han, 2007^118^ | Single-center, China | x |  |  | Treatment for DM, FPG > 140 mg/dL or random glucose >200 mg/dL | x |  |  |  |  | Aggravated or new neurologic deficit |
| Johnston, 2007^119^ | Multi-center, international | x |  |  | Nr | x |  |  |  |  | Recurrent stroke |
| Prosser, 2007^120^ | The Virtual International Stroke Trials Archive, USA | x |  |  | nr |  | x |  |  |  | Nonfatal VT, VF, MI, pulmonary edema/ moderate-severe cardiac failure, or cardiac death |
| Roquer, 2007^121^ | Single-center, Spain | x |  |  | History of DM, treatment for DM, or FPG >126 mg/dL |  |  | x |  |  | All-cause Mortality |
| Toyoda, 2007^122^ | Japan Standard Stroke Registry Study, Japan | x |  |  | Treatment for DM or FPG ≥ 126 mg/dL | x |  |  |  |  | New or progressing neurological deficits and new infarct or hematoma |
| Bateman, 2006^123^ | Nationwide Inpatient Sample, USA | x |  |  | T2DM Diagnosis acquired from patient records |  |  | x |  |  | All-cause Mortality |
| Hamidon, 2006^124^ | Single-center, Malaysia | x |  |  | nr |  |  | x |  |  | All-cause Mortality |
| Kammersgaard, 2006^125^ | Single-center, Denmark | x |  |  | History of DM, or glucose >200 mg/dL |  |  | x |  |  | All-cause Mortality |
| Mateo, 2006^126^ | Single-center, Spain | x |  |  | Treatment for DM, FPG >140 mg/dl, or random glucose >200 mg/dL |  |  | x |  |  | All-cause Mortality |
| Sacco, 2006^127^ | Population based registry, Italy | x |  |  | nr |  |  | x |  |  | All-cause Mortality |
| Tsivgoulis, 2006^128^ | Single-center, Greece | x |  |  | Treatment for DM, FPG >126 mg/dl, or nonfasting glucose >200 mg/dL | x |  |  |  |  | New or progressing neurological deficits or TIA |
| Vermeer, 2006^129^ | Dutch TIA Trial, Netherlands | x | x |  | • History of DM, Treatment for DM, or non-fasting glucose >200 mg/dL • IGT (Non-fasting glucose 140-198 mg/dL) | x | x |  |  | x | • Fatal or nonfatal stroke with new infarct or bleeding • MI and cardiac death • Stroke, MI, cardiac death |
| Xu, 2006^130^ | Nanjing Stroke Registry Program, China | x |  |  | DM definition not reported. Analyzed: Controlled DM defined as FPG < 126 mg/dL | x |  |  |  |  | nr |
| Fu, 2005^131^ | Multi-center, China | x |  |  | Treatment for DM | x |  |  |  |  | A new neurological deficit or an exacerbation of a previous deficit lasting > 24 hours |
| Kaplan, 2005^132^ | Cardiovascular Health Study, US | x |  |  | History of DM or FPG ≥126 mg/dL | x | x | x |  |  | • Fatal or nonfatal IS and -HS  • Fatal or nonfatal MI and deaths attributed to coronary heart disease • All-cause Mortality |
| Kimura, 2005^133^ | Multi-center, Japan | x |  |  | Treatment for DM or HbA1c ≥ 6.5% |  |  | x |  |  | All-cause Mortality |
| Rothwell, 2005^134^ | Oxfordshire Community Stroke Project, UK | x |  |  | Treatment for DM | x |  |  |  |  | A new neurological deficit or an exacerbation of a previous deficit lasting > 24 hours |
| van Wijk, 2005^135^ | The Life Long After Cerebral ischemia cohort based on Dutch TIA Trial, Netherlands | x |  |  | History of DM | x |  | x |  | x | • Fatal or nonfatal Stroke  • All-cause Mortality • Death from all vascular causes, non-fatal stroke, or non-fatal myocardial infarction |
| Kaarisalo, 2005^136^ | FINMONICA stroke register, Finland | x |  |  | History of DM and treatment for DM |  |  | x |  |  | All-cause Mortality |
| Heuschmann, 2004^137^ | German Stroke Registers, Germany | x |  |  | History of DM and treatment for DM or pathological elevated FPG |  |  | x |  |  | All-cause Mortality |
| Hill, 2004^138^ | National registry, Canada | x |  |  | Two physician visits for diabetes over a 2-year period or a single hospitalization for diabetes in a single year. | x |  |  |  |  | IS, SAH, ICH |
| Lee, 2004^139^ | WA Data Linkage System, Australia | x |  |  | nr | x |  |  |  |  | IS |
| Soda, 2004^140^ | TULIPS Stroke registry, Japan | x |  |  | FPG ≥126 mg/dL, 2-hour OGTT ≥ 200 mg/dL, or HbA1c >6.5% | x |  |  |  |  | IS |
| Varona, 2004^141^ | Single-center, Spain | x |  |  | nr | x |  |  |  |  | nr |
| Conway, 2003^142^ | Single-center, UK | x |  |  | History of DM or elevated blood glucose |  |  | x |  |  | All-cause Mortality |
| De Jong, 2003^143^ | Maastricht Stroke Registry, Netherlands | x |  |  | History of DM, treatment for DM, FPG >126 mg/dl, or random glucose >200 mg/dL on two separate times |  |  | x |  |  | All-cause Mortality |
| Hamidon, 2003^144^ | Single-center, Malaysia | x |  |  | • Treatment for DM, FPG >140 mg/dl, or random glucose >200 mg/dL |  |  | x |  |  | All-cause Mortality |
| Lee, 2003^145^ | National Database, Australia | x |  |  | History of DM acquired from patient records |  |  | x |  |  | All-cause Mortality |
| Wong, 2003^146^ | Single-center, Japan | x |  |  | History of DM |  |  |  |  | x | Stroke, TIA, or acute coronary syndrome |
| De Jong, 2002^147^ | Maastricht Stroke Registry, Netherlands | x |  |  | History of DM, treatment for DM, FPG >126 mg/dl, or random glucose >200 mg/dL on two separate times | x |  | x |  |  | new neurological deficit or worsening of a preexistent deficit that could not that occurred at least 72 hours after the first stroke. |
| Johnston, 2000^148^ | Multi-center, USA | x |  |  | nr | x |  |  |  |  | Rapidly developed focal or global disturbance of cerebral function, lasting > 24 hours |
| Petty, 2000^149^ | Rochester Epidemiology Project, USA | x |  |  | FPG > 140 mg/dl on more than one occasion, >200 mg/dL during OGTT, hyperglycemic symptoms | x |  |  |  |  | New neurologic deficit occurred after a period of stability or improvement lasting at least 24 hours |
| Wong, 2000^150^ | Single-center, Japan | x |  |  | nr |  |  |  |  | x | TIA, stroke, acute coronary syndrome |
| Marini, 1999^151^ | Multi-center, Italy | x |  |  | History of DM, FPG > 140 mg/dl on more than one occasion, >200 mg/dL during OGTT, hyperglycemic symptoms |  |  | x |  | x | • All-cause Mortality  • Nonfatal Stroke, nonfatal MI, All-cause death |
| Samsa, 1999^152^ | National database, USA | x |  |  | nr |  |  | x |  |  | All-cause Mortality |
| Wong, 1999^153^ | Multi-center, Asia | x |  |  | Treatment for DM, FPG >140 mg/dl, or random glucose >200 mg/dL |  |  | x |  |  | All-cause Mortality |
| Clavier, 1994^154^ | Single-center, France | x |  |  | History of DM, treatment for DM, FPG >140 mg/dL, glucose after 75g glucose ingestion >200 mg/dL |  |  | x |  |  | All-cause Mortality |
| Hier, 1991^155^ | Stroke Databank, USA | x |  |  | nr | x |  |  |  |  | New cerebrovascular event, different subtype from index event |
| Howard, 1989^156^ | Multi-center, USA | x |  |  | nr |  |  | x |  |  | All-cause Mortality |
| Howard, 1987^157^ | Single-center, USA | x |  |  | nr |  |  | x |  |  | All-cause Mortality |

Studies are listed according to publication year. Abbreviations: IS: Ischemic Stroke, HS: Hemorrhagic Stroke, TIA: transient ischemic attack, DM: Diabetes mellitus; IGT: impaired glucose tolerance; IFG: impaired fasting glucose; FPG: fasting plasma glucose; OGTT: oral glucose tolerance test; MI: myocardial infarction, SAH: subarachnoid hemorrhage; nr: not reported, y: year or years

# Supplementary table 3: Eligible studies for the outcome composite vascular events

| **Study** | **Population** | | | | | **Follow-up² (max)** | **Effect size, 95% CI for Composite Events** | **QOE** |
| --- | --- | --- | --- | --- | --- | --- | --- | --- |
|  | **Stroke type** | **First Ever Event** | **Age¹ (mean)** | **Sex (Men %)** | **n** |  |  |  |
| **Exposure: Diabetes** | | | | | | | | |
| Echouffo-Tcheugui, 2018^39^ | IS | No | 80** | 41.9 | 409060 | 3 y | HR 1.19 (1.18 - 1.20) | G |
| Kang, 2016^56^ | IS | No | 70 | 58.7 | 12227 | 1 y | HR 1.38 (1.21 - 1.57) | G |
| Corrao, 2017^49^ | TIA | Yes | 73.3 | 47.1 | 7776 | 3.3 y* | HR 1.17 (1.03 - 1.33) | G |
| Pan, 2016^57^ | Acute Minor IS and high-risk TIA | No | 62.6 | 66.1 | 5135 | 3 mo | HR 2.42 (2.00 - 2.92) | G |
| Vermeer, 2006^129^ | Minor IS and TIA | No | 65.1 | 65 | 3127 | 2.6 y* | HR 2.5 (1.9 - 3.4) | G |
| Chen, 2017^48^ | Minor IS and High-Risk TIA | No | 62.2 | 66.6 | 3044 | 3 mo | HR 2.54 (1.90 - 3.39) | G |
| Guo, 2021^8^ | Minor IS and TIA | No | 62.2** | 66.5 | 3026 | 3 mo | HR 1.84 (1.40 - 2.43) | G |
| Lin, 2015^62^ | Minor IS and TIA | No | 62.6 | 66.2 | 3021 | 3 mo | HR 1.42 (1.10 – 1.85) | G |
| van Wijk, 2005^135^ | Minor IS and TIA | nr | 65 | 60.2 | 2362¦ | 10.1 y* | HR 2.05 (1.72 - 2.44) | G |
| Yoo, 2017^55^ | IS | No | 63.2 | 64.6 | 1893 | 4.4 y** | HR 1.12 (0.91 – 1.38) | G |
| Pezzini, 2014^75^ | IS | Yes | 36.7 | 51 | 1867 | 3.3 y** | HR 1.49 (0.71 – 3.12) | G |
| Soriano-Reixach, 2018^45^ | IS | Yes | 77** | 49.5 | 933 | 5 y | HR 1.50 (1.18 - 1.90) | G |
| Putaala, 2010^103^ | IS | Yes | 41.5 | 62.9 | 807 | 5 y |  | G |
|  |  |  |  |  |  |  | HR 4.39 (2.28 - 8.45)  (exposure: Type 2 DM) |  |
|  |  |  |  |  |  |  | HR 1.65 (0.87 - 3.12)  *(exposure: Type 1 DM)* |  |
| The, 2021^20^ | IS | No | 60.6 | 71 | 720 | 3.25 y* | HR 1.5 (1.08 - 2.10) | G |
| Wong, 2003^146^ | IS and TIA | No | 67.6 | 58 | 705 | 2.3 y* | OR 1.34 (1.01 – 1.77) | G |
| Altavilla, 2018^35^ | TIA | nr | 69.8 | 47 | 477 | 3.1 y** | OR 3.53 (1.18 - 10.6) | G |
| Marini, 1999^151^ | IS and TIA | Yes | nr | nr | 330 | 8 y* | HR 0.9 (0.2 – 3.9) | G |
| Spengos, 2010^107^ | IS | Yes | 36.9 | 56.5 | 253 | 10 y | HR 3.20 (1.39 – 7.37) | G |
| Busch, 2009^109^ | IS and TIA | No | 63.9 | 55 | 197 | 2.3 y* | HR 1.5 (0.8 – 3.1) | G |
| Wong, 2000^150^ | IS and TIA | No | 67.7 | 58.2 | 705 | 6 mo | OR 0.99 (0.59 – 1.67) | F |
| Rutten-Jacobs, 2013^158^ | IS and TIA | Yes | 40.7 | 47.1 | 656 | 20 y | HR 1.89 (1.12 – 3.19) | F |
| Serena, 2015^67^ | IS (non-cardioembolic) | Yes | 71.3 | 68.4 | 563 | 1 y | HR 2.05 (1.22 - 3.45) | F |
| Rutten-Jacobs, 2014^77^ | IS and TIA (nondiabetic) | No | 40.3 | 44.5 | 427 | 10.1 y* | OR 3.5 (1.5 - 8.4) | F |
| Sagris, 2021^19^ | AIS (cryptogenic) | No | 68 | 66.4 | 264 | 10 y | HR 1.58 (0.98 – 2.55) | F |
| **Exposure: Prediabetes** | | | | | | | | |
| Pan, 2016^57^ | Minor IS and high-risk TIA | No | 62.6 | 66.1 | 5135 | 3 mo | HR 1.56 (19.12 - 2.16 | G |
| Vermeer, 2006^129^ | Minor IS and TIA | No | 65.1 | 65 | 3127 | 2.6 y* | HR 1.4 (09.9 - 2.2) | G |
| Rutten-Jacobs, 2014^77^ | IS and TIA (nondiabetic) | No | 40.3 | 44.5 | 427 | 10.1 y* | OR 2.5 (1.3 - 4.8) | F |
| **Exposure: Insulin Resistance** | | | | | | | | |
| Lu, 2023^1^ | IS | Yes | 61.5 | 68.1 | 6271 | 1 y | OR 0.93 (0.75 – 1.17) | G |
| Jin, 2023^2^ | IS (nondiabetic) | No | 62.6 | 68.3 | 3808 | 1 y | HR 1.46 (1.05 – 2.03) | F |

Studies are listed according to risk of bias assessment and sample size.

When age and data was not available for the whole population, the age and sex data of the most representative subgroup was reported instead. These studies are indicated with ¦. ¹Age is reported in mean, unless marked with ** indicating median. ²Follow-up duration is reported as maximum follow-up unless marked with * indicating mean and ** indicating median. Abbreviations: IS: Ischemic Stroke, TIA: transient ischemic attack, nr: not reported, y: year or years, mo: month or months, DM; diabetes mellitus; QOE: quality of evidence; G: good, F: Fair, P: Poor

# Supplementary table 4: Meta-analysis of absolute risk estimates for all outcomes

|  | **Pooled absolute risk (95% confidence interval)** | | | | | |
| --- | --- | --- | --- | --- | --- | --- |
| **Outcomes** | **Diabetes** | **No Diabetes** | **Prediabetes** | **No prediabetes** | **Insulin resistance** | **No insulin resistance** |
| **Composite outcome** | 43% (23% - 64%) | 17% (3% - 31%) | 31% (12% - 50%) | 7% (5% - 10%) | n.a. | n.a. |
| **Recurrent stroke** | 13% (10% - 16%) | 9% (6% - 11%) | 10% (8% - 12%) | 7% (7% - 8%) | 10% (5% - 15%) | 7% (6% - 7%) |
| **Cardiac events** | 5% (-1% - 11%) | 3% (0% - 6%) | n.a. | n.a. | n.a. | n.a. |
| **Cardiovascular mortality** | 18% (-10% - 47%) | 16% (-9% - 41%) | n.a. | n.a. | n.a. | n.a. |
| **All-cause mortality** | 23% (14% - 31%) | 17% (11% - 23%) | 8% (2% - 15%) | 9% (0% - 18%) | 6% (-1% - 12%) | 4% (2% – 6%) |

n.a. denotes not applicable.

# Supplementary table 5: Eligible studies for the outcome recurrent stroke

| **Study** | **Population** | | | | |  |  |  |
| --- | --- | --- | --- | --- | --- | --- | --- | --- |
|  | **Stroke type** | **First**  **Ever event** | **Age (Mean)** | **Sex (Men %)** | **n** | **Follow-up (max)** | **Effect size, 95% CI for Recurrent Stroke** | **QOE** |
| **Exposure: Diabetes** | | | | | | | | |
| Echouffo-Tcheugui, 2018^39^ | IS | No | 80** | 41.9 | 409060 | 3 y | HR 1.20 (1.18 - 1.23) | G |
| Policardo, 2015^65^ | IS | Yes | 72.9 % >70 y | 53.3 | 43332 | 3 y |  | G |
|  | Age group 16-69 |  |  | 100 | 7694 |  | HR 1.17 (1.02 - 1.34) |  |
|  | Age group 16-69 |  |  | 0 | 4065 |  | HR 1.48 (1.22 - 1.8) |  |
|  | Age group ≥70 |  |  | 100 | 15398 |  | HR 1.07 (0.97 - 1.19) |  |
|  | Age group ≥70 |  |  | 0 | 16175 |  | HR 1.19 (1.06 - 1.33) |  |
| Morton, 2022^11^ | IS | nr | nr | 54 | 25421 | 1 y | HR 1.17 (1.06 - 1.29) | G |
| Ouk, 2020^24^ | IS | Yes | 70.6 | 53.5 | 20201¦ | 5.6 y** | HR 1.18 (1.2 – 1.26) | G |
| Zhang, 2022^13^ | IS and TIA | No | 62.2 | 68.5 | 14674 | 1 y | HR 1.25 (1.00 – 1.55)  *(expoure: DM duration >8 y)* | G |
| Jia, 2011^94^ | IS | No | 67** | 61.8 | 12907 | 6 mo | OR 1.09 (0.98 – 1.22) | G |
| Kang, 2016^56^ | IS | No | 70 | 58.7 | 12227 | 1 y | HR 1.41 (1.19 – 1.67) | G |
| Wang, 2013^86^ | IS | No | 67 | 61.6 | 11560 | 1 y | OR 1.26 (1.08 – 1.47) | G |
| Wang, 2013^85^ |  | No |  |  |  | 1 y |  | G |
|  | IS (Large artery atherosclerosis) |  | 65.6 | 63.4 | 5237 |  | OR 1.18 (1.01 – 1.38) |  |
|  | IS (small artery occlusion) |  | 65.1 | 64.4 | 1938 |  | OR 1.14 (1.08 – 1.93) |  |
|  | IS (cardioembolism) |  | 69.9 | 42 | 714 |  | OR 1.21 (1.06 – 2.36) |  |
|  | IS (other subtypes) |  | 64.7 | 61.2 | 3671 |  | OR 1.25 (1.02 – 1.53) |  |
| Toni, 2014^78^ | IS | No | 53.6 % >65 y | 63.4 | 10578 | 2.5 y* | HR 1.39 (1.10 – 1.77) | G |
| MacIntosh, 2021^17^ | IS | No | 53.6 | 63.7 | 7847 | 6.3 y** | OR 1.37 (1.21 - 1.54) | G |
| Lee, 2004^139^ | IS | Yes | 73.1 | 51.4 | 7816 | 5 y | HR 1.27 (1.07 - 1.51) | G |
| Pan, 2016^57^ | Minor IS and high-risk TIA | No | 62.6 | 66.1 | 5135 | 3 mo | HR 2.38 (1.97 - 2.88) | G |
| Wu, 2015^68^ | Minor IS | No | 65 | 64.5 | 4669 | 3 mo | OR 1.28 (1.02 - 1.60) | G |
| Pan, 2016^58^ | Minor IS | No | 64.1 | 64.9 | 4548 | 1 y | OR 1.23 (0.99 - 1.53) | G |
| Vermeer, 2006^129^ | Minor IS and TIA | No | 65.1 | 65 | 3127 | 2.6 y* | HR 2.8 (1.9 - 4.1) | G |
| Chen, 2017^48^ | Minor IS and High-Risk TIA | No | 62.2 | 66.6 | 3044 | 3 mo | HR 2.53 (1.89 - 3.37) | G |
| Guo, 2021^8^ | Minor IS and TIA | No | 62.2** | 66.5 | 3026 | 3 mo | HR 1.81 (1.38 - 2.39) | G |
| Olaiya, 2021^18^ | TIA | No | 74** | 46.4 | 2750 | 1 y | HR 0.79 (0.50 - 1.24) | G |
| Toyoda, 2007^122^ | IS | No | 71.1 | 63.1 | 2645 | 1 mo | OR 1.48 (1.06 - 2.08) | G |
| van Wijk, 2005^135^ | Minor IS and TIA | nr | 65 | 60.2 | 2362¦ | 10.1 y* | HR 2.02 (1.53 - 2.67) | G |
| Pezzini, 2014^75^ | IS | Yes | 36.7 | 51 | 1867 | 3.3 y** | HR 1.49 (0.68 – 3.26) | G |
| MacDougal, 2018^41^ | IS | No | 70 | 49.6 | 1295 ¦ | 1 y | HR 1.27 (0.78 - 2.07) | G |
| Hier, 1991^155^ | IS | No | 60.4 % >65 y | 47.5 | 1273 | 1.1 y** | HR 1.66 (1.14 - 2.42) | G |
| Jing, 2016^50^ | IS^a^ | Yes | 62.2 | 63.4 | 1210 | 1 y | HR 1.14 (1.01 - 1.28) | G |
| Purroy, 2012^88^ | TIA | No | 68.6 | 59.3 | 1137 | 3 mo | HR 0.91 (0.44 - 1.85) | G |
| Pan, 2017^52^ | Minor IS and TIA | No | 63.1 | 65.4 | 1089 | 3 mo | HR 0.92 (0.55 – 1.53) | G |
| Genceviciute, 2022^7^ | IS treated with thrombectomy | No | 73.1** | 51.1 | 1020 | 3 mo | OR 1.71 (1.02 - 2.87) | G |
| Li, 2015^61^ | IS | Nr | ≥75 | 56 | 871 | 3 y |  |  |
|  | Women |  |  |  |  |  | OR 0.73 (0.37 - 1.46) | G |
|  | Men |  |  |  |  |  | OR 0.85 (0.46 - 1.58) | G |
| Zhang, 2015^69^ | Minor IS | No | 61** | 70.6 | 863 | 1 y | HR 0.57 (0.29 – 1.14) | G |
| Soda, 2004^140^ | IS | No | 72.5 | 57.6 | 829 | 1 y |  | G |
|  | Cardioembolic |  | 76.5 | 50.2 | 205 |  | HR 1.08 (0.42 - 2.74) |  |
|  | Atherothrombotic |  | 71 | 62.4 | 237 |  | HR 0.93 (0.33 - 2.62) |  |
|  | Lacunar |  | 70.3 | 65.7 | 198 |  | HR 3.44 (1.03 - 11.49) |  |
|  | Uncertain cause |  | 72.4 | 50.8 | 189 |  | HR 1.37 (0.41 - 4.52) |  |
| Putaala, 2010^103^ | IS | Yes | 41.5 | 62.9 | 807 | 5 y |  | G |
|  |  |  |  |  |  |  | HR 1.49 (0.71 - 3.13)  (exposure: Type 2 DM) |  |
|  |  |  |  |  |  |  | HR 3.85 (1.80 - 8.23)  *(exposure: Type 1 DM)* |  |
| The, 2021^20^ | IS | No | 60.6 | 71 | 720 | 3.2 y* | HR 1. 54 (1.05 - 2.24) | G |
| Wang, 2023^5^ | IS | No | 61 | 69.5 | 610 | In hospital | HR 1.97 (1.09 – 3.57) | G |
| Kaplan, 2005^132^ | IS | Yes | 80 | 41 | 546 | 3.2 y* | HR 1.59 (1.07 – 2.37) | G |
| Petty, 2000^149^ | IS | Yes | 64.2 | 41 | 454 | 3.2 y* | HR 1.88 (1.25 – 2.85) | G |
| Rothwell, 2005^134^ | TIA | Yes | 71.7 | 47.9 | 399 | 7 days | HR 4.39 (1.36 – 14.2) | G |
| Yokota, 2009^114^ | IS and TIA | No | 70.1 | 66.3 | 371 | 505.4 follow-up y | HR 1.80 (0.68 - 4.78) | G |
| De Jong, 2002^147^ | IS (lacunar stroke) | Yes | nr | nr | 333 | 2.3 y* | HR 2.08 (1.12 - 3.88) | G |
| Fu, 2005^131^ | IS | Yes | 68.3 | 57.0 | 228 | 1.9 y** | HR 2.1 (0.82 – 5.33) | G |
| Tsivgoulis, 2006^128^ | TIA | No | 63.9 | 58.8 | 226 | 1 mo | HR 2.98 (1.28 – 6.94) | G |
| Gulli, 2009^110^ | IS (in posterior circulation) or TIA | No | 68.9 | 61.1 | 216 | 3 mo | HR 1.9 (0.8 – 4.3) | G |
| Han, 2007^118^ | IS (mild-moderate) | No | 69.4 | 52.4 | 210 | 4 y* | HR 2.47 (1.23 - 4.95) | G |
| Tsivgoulis, 2012^89^ | IS and TIA | nr | 64 | 59.1 | 176 | 1 mo | OR 3.67 (0.86 - 15.74) | G |
| Szlachetka, 2020^25^ | IS | Yes | 65.5 | 53.4 | 302603¦ | 4.2 y** | HR 1.27 (1.23 - 1.32) | F |
| Bergström, 2017^47^ | IS | No | 76 | 50 | 196765 | 1 y | HR 1.18 (1.12 - 1.25) | F |
| Li, 2021^15^ | IS and TIA | No | 63 ** | 68.3 | 15166 | 1 y | HR 1.17 (1.04 - 1.32) | F |
| Cao, 2022^6^ | IS | No | 62.3 | 68.7 | 13972 | 3 mo | OR 1.24 (1.07 - 1.44) | F |
| Jin, 2018^40^ | IS | Yes | 38 | 52.4 | 12392 | 3 mo | HR 1.5 (1.22 - 1.84) | F |
| Sun, 2009^113^ |  | Yes | 67.4 | 55.3 | 7785 | 1 y |  | F |
|  | IS |  | 68.1 | 76.6 | 6464 |  | HR 1.07 (0.93 - 1.23) |  |
|  | TIA |  | 63.8 | 56.1 | 1321 |  | HR 1.11 (0.82 - 1.51) |  |
| Johnston, 2007^119^ | TIA | nr | 77% >60 | 47.3 | 4809 | 3 mo | OR 1.70 (1.03 – 2.10) | F |
| Hill, 2004^138^ | TIA | No | 71.4 | 48.9 | 2285 | 1 y | HR 1.35 (1.05 – 1.74) | F |
| Akhtar, 2019^28^ | IS | No | 55.2 | 82 | 1860¦ | 3 mo | OR 1.08 (0.45 - 2.59) | F |
| Johnston, 2000^148^ | TIA | No | 72 | 47.3 | 1707 | 3 mo | OR 2.0 (1.4 – 2.9) | F |
| Li, 2011^95^ | IS | nr | 68 | 59.3 | 1194 | 1 y | HR 1.46 p: 0.001 | F |
| Cruz-Herranz, 2015^60^ | IS | No | 68.9 | 60.4 | 1137 | In-hospital | OR 2.24 p: 0.689 | F |
| Xu, 2006^130^ | IS | Yes | 68.5 | 66.7 | 834 | 1 y |  | F |
|  |  |  |  |  |  |  | HR 1.64 (1.10 - 2.45) *(exposure: controlled diabetes)* |  |
|  |  |  |  |  |  |  | HR 1.69 (1.06 - 2.68) *(exposure: uncontrolled diabetes)* |  |
| Rutten-Jacobs, 2013^158^ | IS and TIA | Yes | 40.7 | 47.1 | 656 | 20 y | HR 1.44 (0.71 – 2.90) | F |
| Yuan, 2020^26^ | IS | Yes | 42.3 | 79.7 | 604 | 3 mo | HR 3.22 (1.85 - 5.62) | F |
| Rutten-Jacobs, 2014^77^ | IS and TIA^a^ | No | 40.3 | 44.5 | 427 | 10.1 y* | OR 1.2 (0.4 - 4.0) | F |
| Nakajima, 2010^102^ | TIA | No | 65 | 71.7 | 113 | 6 mo | OR 1.72 (0.41 – 7.19) | F |
| Varona, 2004^141^ | IS | Yes | 36.6 | 65 | 272 | 12.3 y* | OR 2.5 p: 0.01 | P |
| Vera, 2023^4^ | AIS and TIA (cryptogenic) | nr | 78 | 45 | 78 | 1.9 y** | HR 103 (1.30 – 8261.0) | P |
| **Exposure: Prediabetes** | | | | | | | | |
| Pan, 2016^57^ | Minor IS or high-risk TIA | No | 62.6 | 66.1 | 5135 | 3 mo | HR 1.57 (1.13 - 2.19) | G |
| Jia, 2014^74^ | IS | Yes | 62.5 | 63.7 | 2167 | 1 y | HR 1.14 (0.69 - 1.87) | G |
| Rutten-Jacobs, 2014^77^ | IS and TIA^a^ | No | 40.3 | 44.5 | 427 | 10.1 y* | OR 1.4 (0.6 - 3.3) | F |
| Vermeer, 2006^129^ | Minor IS and TIA | No | 65.1 | 65 | 3127 | 2.6 y* | HR 1.8 (1.1 - 3.0) | G |
| **Exposure: Insulin Resistance** | | | | | | | | |
| Zhou, 2020^27^ | IS | No | 64.8 | 63.5 | 16310 | 1 y | HR 1.32 (1.11 - 1.57) | G |
| Lu, 2023^1^ | IS | Yes | 61.5 | 68.1 | 6271 | 1 y | OR 0.93 (0.75 – 1.17) | G |
| Ago, 2018^34^ | IS | No | 70.3 | 63.5 | 4655¦ | 3 mo | OR 0.96 (0.63 - 1.45) | G |
| Jing, 2017^159^ | IS^a^ | Yes | 62.3 | 63.1 | 1245 | 1 y | HR 2.21 (1.37 - 3.57) | G |
| Pan, 2017^51^ | IS^a^ | Yes | 62.1 | 63.3 | 1203 | 1 y |  | G |
|  |  |  |  |  |  |  | HR 1.65 (1.18 - 2.30) *(exposure: ISI Composite)* |  |
|  |  |  |  |  |  |  | HR 2.75 (2.00 - 3.78) *(exposure: ISI 1,120)* |  |
| Jin, 2023^2^ | IS (nondiabetic) | No | 62.6 | 68.3 | 3808 | 1 y | HR 1.46 (1.05 – 2.03) | F |
| Liu, 2022^10^ | IS^b^ | No | 64.7 | 59.2 | 3359 | 1 y | HR 1.06 (0.60 - 1.85) | F |

Studies are listed according to risk of bias assessment and sample size.

When age and data was not available for the whole population, the age and sex data of the most representative subgroup was reported instead. These studies are indicated with ¦. ¹Age is reported in mean, unless marked with ** indicating median. ²Follow-up duration is reported as maximum follow-up unless marked with * indicating mean and ** indicating median. Studies marked with ^a^ included patients only that are non-diabetic, and ^b^ only diabetic. Abbreviations: IS: Ischemic Stroke, TIA: transient ischemic attack, nr: not reported, y: year or years, mo: month or months, DM; diabetes mellitus; QOE: quality of evidence; G: good, F: Fair, P: Poor

# Supplementary table 6: Eligible studies for the outcome cardiac events

| **Study** | **Population** | | | | |  | |  |  |
| --- | --- | --- | --- | --- | --- | --- | --- | --- | --- |
|  | **Stroke type** | **First-ever event** | **Age**  **(Mean)** | **Sex (Men%)** | **n** | **Follow-up**  **(max)** | **Effect size, 95% CI for Myocardial Infarction** | | **QOE** |
| **Exposure: Diabetes** | | | | | | | | | |
| Hu, 2022^9^ | IS | No | 66.2 | 62.6 | 838229 | In-hospital | OR 1.08 (1.00 - 1.18) | | G |
| Echouffo-Tcheugui, 2018^39^ | IS | No | 80** | 41.9 | 409060 | 3 y | HR 1.55 (1.49 - 1.60) | | G |
| Kang, 2016^56^ | IS | No | 70 | 58.7 | 12227 | 1 y | HR 1.03 (0.59 - 1.77) | | G |
| Pan, 2016^57^ | Acute Minor IS and high-risk TIA | No | 62.6 | 66.1 | 5135 | 3 mo | HR 14.95 (1.15 - 194.16) | | G |
| Vermeer, 2006^129^ | Minor IS and TIA | No | 65.1 | 65 | 3127 | 2.6 y* | HR 2.0 (1.3 - 3.2) | | G |
| Prosser, 2007^120^ | IS | No | 73** | 48.9 | 800¦ | 3 mo | OR 2.13 (1.39 - 3.27) | | G |
| The, 2021^20^ | IS | No | 60.6 | 71 | 720 | 3.2 y* | HR 1.31. (0.62 - 2.75) | | G |
| Kaplan, 2005^132^ | IS | Yes | 80 | 41 | 546 | 3.2 y* | HR 1.70 (1.09 – 2.64) | | G |
| Jin, 2018^40^ | IS | Yes | 38 | 52.4 | 12392 | 3 mo | HR 1.49 (1.17 - 1.88) | | F |
| Rutten-Jacobs, 2013^158^ | IS and TIA | Yes | 40.7 | 47.1 | 656 | 20 y | HR 2.91 (1.53 – 5.57) | | F |
| **Exposure: Prediabetes** | | | | | | | | | |
| Vermeer, 2006^129^ | Minor IS and TIA | No | 65.1 | 65 | 3127 | 2.6 y* | HR 1.0 (0.50 – 2.00) | | G |

Studies are listed according to risk of bias assessment and sample size.

When age and data was not available for the whole population, the age and sex data of the most representative subgroup was reported instead. These studies are indicated with ¦. ¹Age is reported in mean, unless marked with ** indicating median. ²Follow-up duration is reported as maximum follow-up unless marked with * indicating mean and ** indicating median. Studies marked with ^a^ included patients only that are non-diabetic, and ^b^ only diabetic. Abbreviations: IS: Ischemic Stroke, TIA: transient ischemic attack, nr: not reported, y: year or years, mo: month or months, DM; diabetes mellitus; QOE: quality of evidence; G: good, F: Fair, P: Poor

# Supplementary table 7: Eligible studies for the outcome cardiovascular mortality

| **Study** | **Population** | | | | |  |  |  |
| --- | --- | --- | --- | --- | --- | --- | --- | --- |
|  | **Stroke type** | **First-ever event** | **Age (Mean)** | **Sex (Men%)** | **n** | **Follow-up (max)** | **Effect size, 95% CI for Cardiovascular Death** | **QOE** |
| **Exposure: Diabetes** | | | | | | | | |
| Bauza, 2018^37^ | IS Underweight/ normal weight | No | 58.0% ≥65 y | 65.0 | 7864 | 2.4 y** | HR 1.53 (1.23 - 1.89) | G |
| Soriano-Reixach, 2018^45^ | IS | Yes | 77** | 49.5 | 933 | 5 y | HR 2.19 (1.01 - 4.72) | G |
| Pirinen, 2017^53^ | IS | Yes | nr | nr | 690 | 8.8 y* | HR 3.19 (1.43 - 7.10) | G |
| Olaiya, 2021^18^ | TIA | No | 74** | 46.4 | 2750 | 1 y | HR 1.75 (1.06 - 2.91) | G |
| Winell, 2011^96^ | IS | Yes | 54.4% > 75-94 Y | 45.1 | 115208 | 1 y |  | P |
|  | 35-74 years |  |  | 0 | 20796 | 1 y | HR 2.17 (1.71 - 2.74) |  |
|  | 35-74 years |  |  | 100 | 31748 | 1 y | HR 1.43 (1.15 - 1.77) |  |
|  | 75-94 years |  |  | 0 | 42482 | 1 y | HR 1.45 (1.28 - 1.63) |  |
|  | 75-94 years |  |  | 100 | 20182 | 1 y | HR 1.62 (1.33 - 1.96) |  |
| **Exposure: Insulin Resistance** | | | | | | | | |
| Balti, 2013^81^ | IS | Yes | 61.5 | 55 | 45 | 5 y |  | G |
|  |  |  |  |  |  |  | HR 0.81 (0.36 - 1.84) *(exposure: Glucose/Insulin Ratio)* |  |
|  |  |  |  |  |  |  | HR 1.07 (0.89 - 1.28) *(exposure: QUICKI Index)* |  |
|  |  |  |  |  |  |  | HR 0.04 (0 - 107.94) *(exposure: HOMA-IR)* |  |

Studies are listed according to risk of bias assessment and sample size.

¹Age is reported in mean, unless marked with ** indicating median. ²Follow-up duration is reported as maximum follow-up unless marked with * indicating mean and ** indicating median Abbreviations: IS: Ischemic Stroke, TIA: transient ischemic attack, nr: not reported, y: year or years, mo: month or months, DM; diabetes mellitus; QOE: quality of evidence; G: good, F: Fair, P: Poor

# Supplementary table 8: Eligible studies for the outcome all-cause mortality

| **Study** | **Population** | | | | |  |  |  |
| --- | --- | --- | --- | --- | --- | --- | --- | --- |
|  | **Stroke type** | **First-ever Event** | **Age (mean)** | **Sex (Men %)** | **n** | **Follow-up (max)** | **Effect size, 95% CI for All-cause Mortality** | **QOE** |
| **Exposure: Diabetes** | | | | | | | | |
| Hu, 2022^9^ | IS | No | 66.2 | 62.6 | 838229 | in-hosp. | OR 1.33 (1.24 – 1.42) | G |
| Reeves, 2010^105^ | IS | No | 71 | 47.4 | 410418¦ | in-hosp. | OR 1.12 (1.08 - 1.15) | G |
| Echouffo-Tcheugui, 2018^39^ | IS | No | 80** | 41.9 | 409060 | 3 y | HR 1.24 (1.23 - 1.25) | G |
| Wood, 2016^59^ | IS | No | nr | nr | 306154 | in-hosp. | OR 1.11 (1.07 - 1.16) | G |
| Bateman, 2006^123^ | IS | nr |  |  |  | in-hosp |  | G |
|  | IS^a^ |  | 66.1 % >65 y | 54.1 | 2594 |  | OR 1.31 (0.97 – 1.76) |  |
|  | IS (without thrombolysis) |  | 75.7% >65 y | 43.8 | 246370 |  | OR 1.01 (0.97 – 1.04) |  |
| Hanchate, 2013^83^ | IS | No | 70.7 % >65 y | 46.3 | 147780 | in-hosp. | OR 0.97 (0.92 – 1.03) | G |
| Lopez-de-Andres, 2021^16^ | IS | No |  |  |  | in-hosp. |  | G |
|  | Men |  | 72.51 | 100 | 56802 |  | OR 1.03 (0.97 - 1.10) |  |
|  | Women |  | 79.17 | 0 | 44004 |  | OR 1.06 (0.99 - 1.13) |  |
| Read, 2018^43^ | IS and Stroke of undetermined cause | Yes | nr | 48.5 | 69757 | 1 mo |  | G |
|  | Men |  |  | 100 | 33813 |  | OR 1.15 (1.05 - 1.26) |  |
|  | Women |  |  | 0 | 35944 |  | OR 1.18 (1.09 - 1.29) |  |
| Policardo, 2015^66^ | IS | nr | nr | nr | 65165 | in-hosp. |  | G |
|  | Men |  |  |  |  |  | OR 0.97 (0.81 - 1.16) |  |
|  | Women |  |  |  |  |  | OR 1.19 (1.04 - 1.36) |  |
| Tsivgoulis, 2019^33^ | IS^a^ | No | 70.1 | 54.6 | 54206 | 3 mo | OR 1.52 (1.41 - 1.64) | G |
| Zamir, 2021^21^ | IS | Yes | 71.5 | 52.9 | 41639 | 5 y |  | G |
|  | 20-29 |  |  |  |  |  | HR 13.9 (7.60 – 25.40) |  |
|  | 30-39 |  |  |  |  |  | HR 9.60 (5.80 – 15.80) |  |
|  | 40-49 |  |  |  |  |  | HR 6.60 (4.40 - 9.90) |  |
|  | 50-59 |  |  |  |  |  | HR 4.50 (3.30 – 6.20) |  |
|  | 60-69 |  |  |  |  |  | HR 3.10 (2.50- 3.90) |  |
|  | 70-79 |  |  |  |  |  | HR 2.10 (1.90 – 2.50) |  |
|  | 80-89 |  |  |  |  |  | HR 1.50 (1.30 – 1.60) |  |
|  | 90+ |  |  |  |  |  | HR 1.00 (0.90 – 1.20) |  |
| Masrur, 2015^63^ | IS^a^ | No | 70** | 50.3 | 31505 | in-hosp. | OR 1.36 (1.21 - 1.53) | G |
| Andersen, 2011^90^ | IS | Yes | 71.2 | 51.5 | 26818 | 1 y | OR 1.33 (1.06 - 1.69) | G |
| Morton, 2022^11^ | IS | nr | nr | 54 | 25421 | 1 y | HR 1.11 (1.03 - 1.19) | G |
| Ouk, 2020^24^ | IS | Yes | 70.6 | 53.5 | 23579 | in-hosp. | OR 1.47 (1.01 - 2.14) | G |
| Zhang, 2022^13^ | IS and TIA | No | 62.2 | 68.5 | 14674 | 1 y | HR 1.66 (1.09 - 2.52)  *expoure:*  *DM duration >8 y)* | G |
| Wang, 2013^86^ | IS | No | 67 | 61.6 | 11560 | 1 y | OR 1.30 (1.09 – 1.54) | G |
| Zhao, 2014^80^ | IS | No | 65** | 62.1 | 10905 | 3 mo¦ | OR 1.10 (0.91 – 1.33) | G |
| Heuschmann, 2004^137^ | IS (without thrombolysis) | No |  |  | 10800 | in-hosp. |  | G |
|  |  |  | 73.2 | 0 | 5049 |  | HR 0.90 (0.7 – 1.1) |  |
|  |  |  | 67.3 | 100 | 5751 |  | HR 1.30 (1.0 – 1.8) |  |
| Bauza, 2018^37^ | IS (Underweight or normal weight) | No | 54.9% ≥65 y | 64.1 | 7864 | 2.4 y** | HR 1.47 (1.24 - 1.73) | G |
| MacIntosh, 2021^17^ | IS | No | 53.6 | 63.7 | 7847 | 6.3 y | OR 1.68 1.497 - 1.882) | G |
| Pan, 2016^57^ | Minor IS and high-risk TIA | No | 62.6 | 66.1 | 5135 | 3 mo | HR 1.33 (0.44 - 4.04) | G |
| Wu, 2015^68^ | Minor IS | No | 65 | 64.5 | 4669 | 3 mo | OR 0.90 (0.61 - 1.34) | G |
| Tram, 2022^3^ | IS | Yes | 72.8 | 57.9 | 4636 | 2.7 y* |  | G |
|  |  |  |  |  |  |  | HR 1.24 (1.06 – 1.46) exposure: good controlled DM |  |
|  |  |  |  |  |  |  | HR 1.27 (1.08 – 1.50) exposure: poor controlled DM |  |
| Pan, 2016^58^ | Minor IS | No | 64.1 | 64.9 | 4548 | 1 y | OR 1.05 (0.77 - 1.44) | G |
| Kaarisalo, 2005^136^ | IS | Yes | 72.4 | 43.6 | 4390 | 1 mo | OR 1.20 (1.00 - 1.45) | G |
| Chen, 2018^38^ | IS^a^ | nr | 67 | 62 | 3288 | 3 mo | OR 0.83 (0.51 - 1.36) | G |
| Olaiya, 2021^18^ | TIA | No | 74* | 46.4 | 2750 | 1 y | HR 1.81 (1.35 - 2.43) | G |
| Wong, 1999^153^ | IS | No | 63.7 | 58.3 | 2403 | in-hosp. | OR 1.52 (1.04 - 2.22) | G |
| van Wijk, 2005^135^ | Minor IS and TIA | nr | 65 | 60.2 | 2362¦ | 10.1 y* | HR 1.94 (1.64 - 2.30) | G |
| Lee, 2003^145^ | TIA | Yes | 72.6 | 50.5 | 1974 | nr | HR 1.66 (1.25 -2.22) | G |
| Wu, 2014^79^ | IS | Yes | 62.8 | 62.6 | 1779¦ | 1 y | OR 1.48 (1.08 - 2.02) | G |
| Wu, 2010^108^ | Lacunar Stroke | Yes | 65.6 | 61.3 | 1650 | 1 y | OR 1.36 (1.00 - 1.81) | G |
| Roquer, 2007^121^ | IS | Yes | 73 | 50.5 | 1527 | in-hosp. | HR 0.85 (0.62 - 1.16) | G |
| Fuentes, 2012^87^ | IS^a^ | No | 67.9 | 53.1 | 1475 | 3 mo | OR 1.09 (0.67 - 1.77) | G |
| Erdur, 2014^72^ | IS | No | 73** | 54 | 1335 | in-hosp. | OR 1.05 (0.48 – 2.27) | G |
| MacDougal, 2018^41^ | IS | No | 70 | 49.6 | 1260¦ | 1 y | HR 1.47 (1.09 - 1.97) | G |
| Jing, 2016^50^ | IS^b^ | Yes | 62.2 | 63.4 | 1251 | 1 y | HR 1.12 (1.00 - 1.26) | G |
| Roquer, 2014^76^ | IS | Yes | 77** | 49.5 | 1088 | 3 mo | OR 1.09 (0.65 - 1.71) | G |
| Fang, 2020^22^ | IS^a^ | No | 63.4* | 60.9 | 1084 | 3 mo | OR 1.36 (0.82 - 2.25) | G |
| Genceviciute, 2022^7^ | IS^c^ | No | 73.1** | 51.1 | 1020 | 3 mo | OR 1.71 (1.02 - 2.87) | G |
| De Jong, 2003^143^ | IS | Yes | 71 | nr | 998 | 1 mo | OR 2.14 (1.32 – 3.48) | G |
| Aarnio, 2014^70^ | IS | Yes | 44** | 62.7 | 970 | 17 y | HR 4.15 (2.26 – 7.61) | G |
| Soriano-Reixach, 2018^45^ | IS | Yes | 77** | 49.5 | 933 | 5 y | HR 1.48 (1.20 - 1.81) | G |
| Kammersgaard, 2006^125^ | IS | No | 73.7 | 51.9 | 899¦ | 5 y | HR 1.30 (1.00 - 1.60) | G |
| Li, 2015^61^ | IS | nr |  |  | 871 | 3y |  | G |
|  |  |  | ≥ 75 y | 0 | 383 |  | OR 0.97 (0.46 – 2.07) |  |
|  |  |  | ≥ 75 y | 100 | 488 |  | OR 0.70 (0.36 – 1.37) |  |
| Kim, 2021^14^ | IS^a^ | No | 60 | 68.6 | 661 | in-hosp. | OR 2.52 (0.83 - 7.60) | G |
| Putaala, 2010^104^ | IS^a^ | No | 69** | 55.2 | 851 | 3 mo | OR 0.82 (0.26 – 2.62) | G |
| Conway, 2003^142^ | IS and Stroke of undetermined cause | No | 74 | 54 | 832 | 87 d** | OR 1.22 (0.95 - 1.56) | G |
| Staals, 2008^117^ | IS | Yes | 70.7 | 52.3 | 782 | 15 y* | HR 1.37 (1.11 – 1.70) | G |
| Pirinen, 2017^53^ | IS | Yes | nr | nr | 690 | 8.8 y* | HR 3.11 (1.55 - 6.22) | G |
| Greisenegger, 2011^92^ | IS and TIA | No | 50.2 | 65 | 661 | 5 y | HR 1.87 (1.10 – 3.19) | G |
| Kaplan, 2005^132^ | IS | Yes | 80 | 41 | 546 | 3.2 y* | HR 1.26 (0.97 – 1.64)) | G |
| Mateo, 2006^126^ | IS | No | 75.5 | 49.2 | 504 | 1 mo | OR 1.92 (1.05 - 3.52) | G |
| Mengel, 2019^31^ | IS | No | 70 | 57.8 | 434 | 3 mo | HR 0.74 (0.19 - 2.88) | G |
| Arnold, 2014^71^ | IS^a^ | No | 64 | 54.8 | 389 | 3 mo | OR 1.44 (0.71 - 2.89) | G |
| Gomes, 2013^82^ | IS | No | 41.9% > 65 y | 51.3 | 351 | 1 mo | HR 1.89 (1.45 – 3.11) | G |
| De Jong, 2002^147^ | IS (lacunar Stroke) | Yes | nr | nr | 333 | 2.3 y* | HR 1.62 (1.00 – 2.61) | G |
| Marini, 1999^151^ | IS and TIA | Yes | nr | nr | 330 | 8 y* | HR 0.7 (0.1 – 5.8) | G |
| Hassan, 2010^99^ | IS | No | 64.6 | 60.2 | 327 | in-hosp. | OR 2.4 (1.2 - 5.0) | G |
| Hassan, 2011^93^ | IS | No | 64.8 | 59.3 | 273 | in-hosp. | OR 2.0 (1.0 - 3.8) | G |
| Meurer, 2010^101^ | IS^a^ | nr | nr | nr | 273 | in-hosp. | OR 1.99 (0.89 - 4.46) | G |
| Gunarathne, 2008^115^ | IS | Yes | 68 | 59 | 242 | 5 y | HR 1.66 (1.02 - 2.6) | G |
| Clavier, 1994^154^ | IS (lacunar Stroke) | No | nr | 62.7 | 177 | 2.9 y | HR 2.71 (0.6 - 12) | G |
| Fonarow, 2010^98^ |  |  |  |  |  | in-hosp. |  | F |
|  | IS | No | 73** | 47.5 | 601599 |  | OR 1.11 (1.08 – 1.14) |  |
|  | TIA | No | 74** | 43.1 | 227788 |  | OR 1.22 (1.01 – 1.47) |  |
| Muñoz-Rivas, 2015^64^ | IS | No | 74.2 | 52.2 | 423475 | in-hosp. |  | F |
|  | Men |  | 71.3 | 100 | 221418 |  | OR 0.98 (0.95 - 1.02) |  |
|  | Women |  | 77.5 | 0 | 202057 |  | OR 1.07 (1.05 - 1.11) |  |
| Szlachetka, 2020^25^ | IS | Yes | 65.5 | 53.4 | 370527 |  | HR 1.54 (1.27 - 1.86) | F |
| Smith, 2010^106^ | IS | No | 71.6 | 46.5 | 274988 | in-hosp. | OR 1.11 (1.05 - 1.16) | F |
| Samsa, 1999^152^ | IS and TIA | No | 78.8 | 40.2 | 49333 | 2 y | HR 1.14 p= 0.001 | F |
| Kamalesh, 2008^116^ | IS | nr | 67.5 | 98.2 | 48733 | 1 y | HR 1.15 (1.11 - 1.19) | F |
| Ahmed, 2010^97^ | IS^a^ | No | 69** | 58.8 | 16049 | 3 mo | OR 1.31 (1.12 - 1.53) | F |
| Cao, 2022^6^ | IS | No | 62.3 | 68.7 | 13972 | 3 mo | OR 1.36 (0.99 - 1.87) | F |
| Kimura, 2005^133^ | IS and TIA | No | 70.4 | 63.2 | 10981 | 9 mo** | HR 1.42 (1.17 – 1.71) | F |
| Forti, 2020^23^ | IS | Nr | 79 | 46.9 | 2076 | 1 y | HR 1.30 (0.90 - 1.70) | F |
| Rocco, 2013^84^ | IS^a^ | Nr | 74** | 52.4 | 1112 | 3 mo | OR 1.57 (1.02 - 2.43) | F |
| Koton, 2010^100^ | IS | Yes | 71 | 53 | 1079 | 3y | HR 1.60 (1.00 – 2.40) | F |
| Putaala, 2009^112^ | IS | IS | 41.5 | 62.8 | 731 | 5 y |  | F |
|  |  |  |  |  |  |  | HR 2.19 (0.88 – 5.48) *(exposure: type 1 diabetes)* |  |
|  |  |  |  |  |  |  | HR 1.23 (0.52 – 2.90) *(exposure: type 2 diabetes)* |  |
| Sun, 2009^113^ | IS | Yes | 68.1 | 76.6 | 6464 | in-hosp. | HR 1.44 (1.16 - 1.79) | F |
| Bauza, 2018^36^ | IS^a^ | No | 58.4% > 65 y | 51.8 | 645 | 1 y | HR 0.98 (0.64 - 1.51) | F |
| Choi, 2019^29^ | IS^c^ | No | 69.6 | 52 | 534 | 3 mo | OR 4.32 (2.41 - 7.75) | F |
| Stefanovic, 2017^54^ | IS | No | 57.5 | 69.5 | 518 | 3 y** | HR 2.12 (1.09 - 5.46) | F |
| Hjalmarsson, 2014^73^ | IS | No | 78.8 | 48.5 | 501 | 1 y | HR 3.06 (1.07 - 8.73) | F |
| Sacco, 2006^127^ | Lacunar Stroke | Yes | 72.7 | 51.3 | 491 | 5 y | HR 1.56 (1.10 - 2.21) | F |
| Sagris, 2021^19^ | IS (cryptogenic) | No | 68 | 66.4 | 264 | 10 y | HR 1.61 (1.00 – 2.59) | F |
| Burns, 2011^91^ | TIA | nr | 71 | 41 | 456 | 10.2 y** | HR 1.54 (1.05 - 2.28) | F |
| Osei, 2018^42^ | IS^a^ | nr | 64 | 47.7 | 220 | 3 mo | OR 4.45 (1.36 - 14.52) | F |
| de Oliveira, 2019^30^ | IS and Stroke of undetermined cause | No | 85.3 | 49.2 | 195 | 1 mo | OR 3.41 (1.30 - 8.87) | F |
| Hamidon, 2003^144^ | IS | nr | 62.1 | 47.8 | 163 | in-hosp. | OR 4.88 (1.25 - 19.1) | F |
| Howard, 1989^156^ | IS | No | 70** | 50 | 1629 | 1 y | HR 1.35 p= 0.016 | P |
| Howard, 1987^157^ | TIA | No | 40.1 % >65 y | 55.6 | 451 | 8 y | HR 2.11 p: 0.02 | P |
| Oksala, 2009^111^ | IS | No | 70.8 | 48.5 | 396 | 7.5 y* | HR 1.33 (0.98 - 1.81) | P |
| Sarfo, 2018^44^ | IS | nr | 58.1 | 50.4 | 249¦ | 2.6 y* | HR 2.83 (1.31 - 6.12) | P |
| Panni, 2019^32^ | IS^c^ | nr | 65.7 | 59.3 | 216 | 3 mo | OR 8.04 (2.30 - 28.60) | P |
| Hamidon, 2006^124^ | IS | Yes | 64.4 | 50 | 134 | 1 mo | OR 1.33 (0.43 - 4.10) | P |
| **Exposure: Prediabetes** | | | | | | | |  |
| Pan, 2016^57^ | Minor IS and high-risk TIA | No | 62.6 | 66.1 | 5135 | 3 mo | HR 3.2 (0.83 - 12.39) | G |
| Jia, 2014^74^ | IS | Yes | 62.5 | 63.7 | 2167 | 1 y | HR 3.09 (1.39 - 6.88) | G |
| Roquer, 2014^76^ | IS | Yes | 77** | 49.5 | 1088 | 3 mo | OR 0.66 (0.38 - 1.17) | G |
| Kim, 2021^14^ | IS^a^ | No | 60 | 68.6 | 661 | in-hosp. | OR 3.12 (01.06 - 9.09) | G |
| Forti, 2020^23^ | IS | Nr | 79 | 46.9 | 2076 | 1 y | HR 0.90 (0.69 - 1.18) | F |
| Osei, 2018^42^ | IS^a^ | nr | 64 | 47.7 | 220 | 3 mo | OR 1.63 (0.62 - 4.34) | F |
| **Exposure: Insulin Resistance** | | | | | | | |  |
| Zhou, 2020^27^ | IS | No | 64.8 | 63.5 | 16310 | 1 y | HR 1.25 (1.06 - 1.47)  *(exposure: TyG Index Q4)* | G |
| Ago, 2018^34^ | IS | No | 70.3 | 63.5 | 4655¦ | 3 mo | OR 1.39 (0.76 - 2.57)  *(exposure: HOMA-IR Q5)* | G |
| Wu, 2014^79^ | IS | Yes | 62.8 | 62.6 | 1779¦ | 1 y | OR 0.95 (0.69 - 1.29)  (exposure: HOMA-IR>1) | G |
| Jing, 2017^159^ | IS^b^ | Yes | 62.3 | 63.1 | 1245 | 1 y | HR 2.59 (1.43 - 4.70)  (exposure: HOMA-IR Q4) | G |
| Yang, 2022^12^ | IS^b^ | Yes | 62 ** | 63.3 | 1226 | 1 y | HR 2.91 (1.62 - 5.23)  (exposure: TyG Index Q4) | G |
| Pan, 2017^51^ | IS^b^ | Yes | 62.1 | 63.3 | 1203 | 1 y |  | G |
|  |  |  |  |  |  |  | HR 2.22 (1.49 - 3.29) *(exposure: ISI Composite)* |  |
|  |  |  |  |  |  |  | HR 3.99 (2.69 - 5.91) *(exposure: ISI 1,120)* |  |
| Balti, 2013^81^ | IS | Yes | 61.5 | 55 | 45 | 5 y |  | G |
|  |  |  |  |  |  |  | HR 0.85 (0.46 - 1.58) *(exposure: Glucose/Insulin Ratio)* |  |
|  |  |  |  |  |  |  | HR 1.03 (0.86 - 1.25) *(exposure: QUICKI Index)* |  |
|  |  |  |  |  |  |  | HR 0.26 (0.1 - 72.86) *(exposure: HOMA-IR)* |  |
| Jin, 2023^2^ | IS (nondiabetic) | No | 62.6 | 68.3 | 3808 | 1 y | HR 0.70 (0.38 – 1.29) | F |
| Liu, 2022^10^ | IS^d^ | No | 64.7 | 59.2 | 3359 | 1 y | HR 1.67 (0.76 - 3-69)  (exposure: TyG Index Q4) | F |
| Zhou, 2018^46^ | IS^b^ ( with GFR > 90 mL/min/1.73 m2) | Yes | 62.2 | 63.5 | 1196 | 1 y |  | F |
|  |  |  | 62.2 | 63.5 | 761 |  | HR 2.88 (1.30-6.37) *(exposure: HOMA-IR)* |  |
|  |  |  | 62.2 | 63.5 | 761 |  | HR 2.43 (1.18-4.99) *(exposure: ISI)* |  |

Studies are listed according to risk of bias assessment and sample size.

When age and data was not available for the whole population, the age and sex data of the most representative subgroup was reported instead. These studies are indicated with ¦. ¹Age is reported in mean, unless marked with ** indicating median. ²Follow-up duration is reported as maximum follow-up unless marked with * indicating mean and ** indicating median. Studies marked with ^a^ only included patients treated with thrombolysis, ^b^ only included patients without diabetes, ^c^ only included patients treated with thrombectomy, ^d^ only included patients with diabetes Abbreviations: IS: Ischemic Stroke, TIA: transient ischemic attack, nr: not reported, y: year or years, mo: month or months, DM; diabetes mellitus; QOE: quality of evidence; G: good, F: Fair, P: Poor; GFR: glomerular filtration rate

# Supplementary figure 1: Risk of bias assessment of the studies included in the review


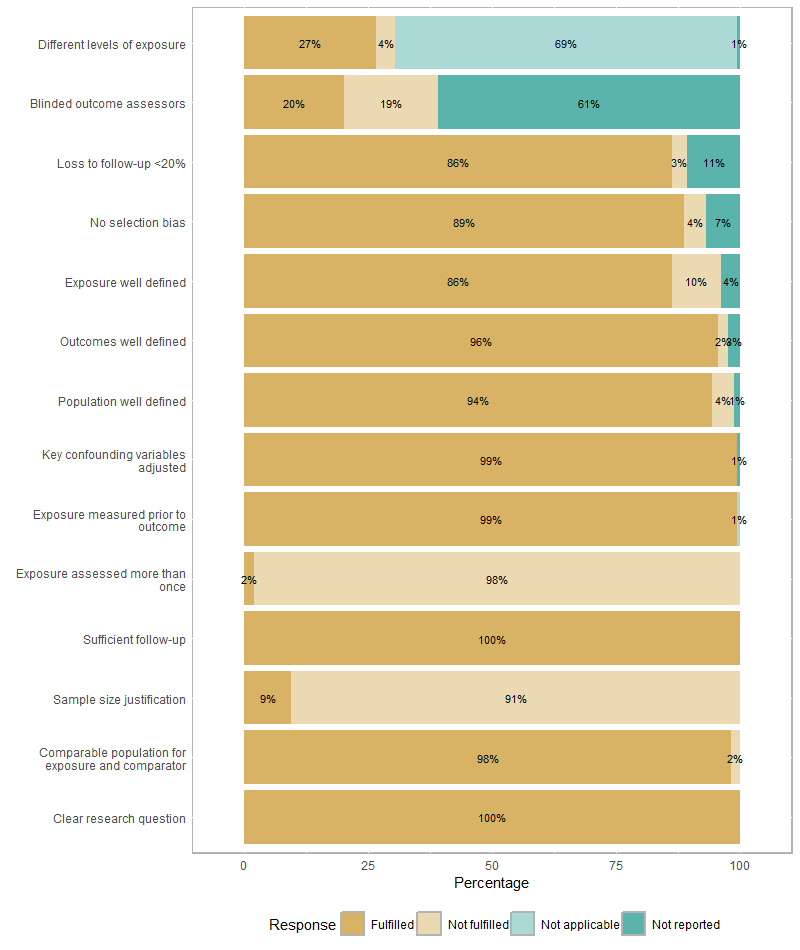


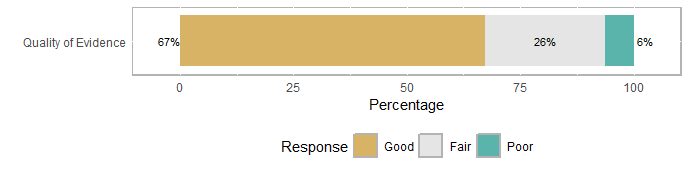


**Risk of bias assessment of the individual studies**

| **Study** | **Q1** | **Q2** | **Q3** | **Q4** | **Q5** | **Q6** | **Q7** | **Q8** | **Q9** | **Q10** | **Q11** | **Q12** | **Q13** | **Q14** | **QOE** |
| --- | --- | --- | --- | --- | --- | --- | --- | --- | --- | --- | --- | --- | --- | --- | --- |
| Vera, 2023 | Yes | Yes | n.r. | Yes | No | Yes | Yes | No | Yes | No | No | n.r. | n.r. | Yes | Poor |
| Wang, 2023 | Yes | Yes | Yes | Yes | No | Yes | Yes | Yes | Yes | No | Yes | n.r. | Yes | Yes | Good |
| Jin, 2023 | Yes | Yes | No | Yes | No | Yes | Yes | Yes | Yes | No | Yes | n.r. | Yes | Yes | Fair |
| Lu Z, 2023 | Yes | Yes | Yes | Yes | No | Yes | Yes | Yes | Yes | No | Yes | n.r. | Yes | Yes | Good |
| Cao, 2022 | Yes | No | Yes | Yes | No | Yes | Yes | n.a. | Yes | No | Yes | n.r. | Yes | Yes | Fair |
| Genceviciute, 2022 | Yes | Yes | Yes | Yes | No | Yes | Yes | Yes | Yes | No | Yes | n.r. | Yes | Yes | Good |
| Guo, 2022 | Yes | Yes | Yes | Yes | No | Yes | Yes | Yes | Yes | No | Yes | n.r. | Yes | Yes | Good |
| Morton, 2022 | Yes | Yes | Yes | Yes | No | Yes | Yes | n.a. | Yes | No | Yes | n.r. | Yes | Yes | Good |
| Zhang, 2022 | Yes | Yes | Yes | Yes | No | Yes | Yes | n.a. | Yes | No | Yes | Yes | Yes | Yes | Good |
| Liu, 2022 | Yes | Yes | No | Yes | No | Yes | Yes | Yes | Yes | No | Yes | n.r. | Yes | Yes | Fair |
| Hu, 2022 | Yes | Yes | Yes | Yes | No | Yes | Yes | Yes | Yes | No | Yes | n.r. | Yes | Yes | Good |
| Tram, 2022 | Yes | Yes | Yes | Yes | No | Yes | Yes | Yes | Yes | No | Yes | n.r. | Yes | Yes | Good |
| Yang, 2022 | Yes | Yes | Yes | Yes | No | Yes | Yes | Yes | Yes | No | Yes | Yes | Yes | Yes | Good |
| MacIntosh, 2021 | Yes | Yes | Yes | Yes | No | Yes | Yes | n.a. | Yes | No | Yes | n.r. | Yes | Yes | Good |
| Olaiya, 2021 | Yes | Yes | Yes | Yes | No | Yes | Yes | n.a. | Yes | No | Yes | n.r. | Yes | Yes | Good |
| The, 2021 | Yes | Yes | Yes | Yes | No | Yes | Yes | n.a. | Yes | No | Yes | n.r. | Yes | Yes | Good |
| Li, 2021 | Yes | Yes | No | Yes | No | Yes | Yes | n.a. | Yes | No | Yes | n.r. | Yes | Yes | Fair |
| Kim, 2021 | Yes | Yes | Yes | Yes | No | Yes | Yes | No | Yes | No | Yes | n.r. | Yes | Yes | Good |
| Lopez-de-Andres, 2021 | Yes | Yes | Yes | Yes | No | Yes | Yes | n.a. | Yes | No | Yes | n.r. | Yes | Yes | Good |
| Zamir, 2021 | Yes | Yes | Yes | Yes | No | Yes | Yes | n.a. | Yes | No | Yes | n.r. | Yes | Yes | Good |
| Sagris, 2021 | Yes | Yes | Yes | Yes | No | Yes | Yes | n.a. | No | No | Yes | n.r. | n.r. | Yes | Fair |
| Szlachetka, 2020 | Yes | No | Yes | Yes | Yes | Yes | Yes | No | Yes | Yes | Yes | No | Yes | Yes | Fair |
| Yuan, 2020 | Yes | Yes | n.r. | Yes | No | Yes | Yes | n.a. | Yes | No | Yes | n.r. | n.r. | Yes | Fair |
| Ouk, 2020 | Yes | Yes | Yes | Yes | Yes | Yes | Yes | n.a. | Yes | No | Yes | n.r. | Yes | Yes | Good |
| Zhou, 2020 | Yes | Yes | Yes | Yes | No | Yes | Yes | Yes | Yes | No | Yes | Yes | Yes | Yes | Good |
| Forti, 2020 | Yes | Yes | Yes | No | No | Yes | Yes | Yes | Yes | No | Yes | No | No | Yes | Fair |
| Fang, 2020 | Yes | Yes | Yes | Yes | No | Yes | Yes | Yes | Yes | No | Yes | n.r. | Yes | Yes | Good |
| Akhtar, 2019 | Yes | Yes | Yes | Yes | No | Yes | Yes | Yes | Yes | No | Yes | n.r. | No | Yes | Fair |
| Mengel, 2019 | Yes | Yes | Yes | Yes | No | Yes | Yes | n.a. | Yes | No | Yes | n.r. | Yes | Yes | Good |
| de Oliveira, 2019 | Yes | Yes | Yes | Yes | No | Yes | Yes | n.a. | Yes | No | n.r. | n.r. | Yes | Yes | Fair |
| Tsivgoulis, 2019 | Yes | Yes | Yes | Yes | No | Yes | Yes | Yes | Yes | No | Yes | n.r. | Yes | Yes | Good |
| Choi, 2019 | Yes | Yes | n.r. | Yes | No | Yes | Yes | Yes | Yes | No | Yes | n.r. | n.r. | Yes | Fair |
| Panni P, 2019 | Yes | No | n.r. | Yes | No | Yes | Yes | n.a. | No | No | Yes | Yes | n.r. | Yes | Poor |
| Echouffo-Tcheugui, 2018 | Yes | Yes | Yes | Yes | No | Yes | Yes | Yes | Yes | No | Yes | No | Yes | Yes | Good |
| Jin, 2018 | Yes | No | Yes | Yes | No | Yes | Yes | n.a. | Yes | No | Yes | n.r. | Yes | Yes | Fair |
| MacDougal, 2018 | Yes | Yes | Yes | Yes | Yes | Yes | Yes | n.a. | Yes | No | Yes | No | Yes | Yes | Good |
| Ago, 2018 | Yes | Yes | Yes | Yes | No | Yes | Yes | Yes | Yes | No | Yes | Yes | Yes | Yes | Good |
| Sarfo, 2018 | Yes | n.r. | Yes | Yes | No | Yes | Yes | n.a. | Yes | No | Yes | No | No | Yes | Poor |
| Bauza, 2018 | Yes | Yes | Yes | Yes | No | Yes | Yes | n.a. | Yes | No | Yes | Yes | n.r. | Yes | Fair |
| Read, 2018 | Yes | Yes | Yes | Yes | No | Yes | Yes | n.a. | Yes | No | Yes | n.r. | Yes | Yes | Good |
| Chen, 2018 | Yes | Yes | Yes | Yes | No | Yes | Yes | NA | Yes | No | Yes | Yes | Yes | Yes | Good |
| Soriano-Reixach, 2018 | Yes | Yes | Yes | Yes | No | Yes | Yes | n.a. | Yes | No | Yes | No | Yes | Yes | Good |
| Bauza, 2018 | Yes | Yes | Yes | Yes | No | Yes | Yes | n.a. | Yes | No | Yes | Yes | Yes | Yes | Good |
| Osei, 2018 | Yes | Yes | n.r. | Yes | No | Yes | Yes | Yes | Yes | No | Yes | Yes | n.r. | Yes | Fair |
| Zhou, 2018 | Yes | Yes | Yes | Yes | No | Yes | Yes | Yes | Yes | No | Yes | Yes | No | Yes | Fair |
| Altavilla, 2018 | Yes | Yes | Yes | Yes | Yes | Yes | Yes | n.a. | Yes | No | Yes | n.r. | Yes | Yes | Good |
| Chen, 2017 | Yes | Yes | Yes | Yes | No | Yes | Yes | n.a. | Yes | No | Yes | Yes | Yes | Yes | Good |
| Bergström L, 20 | Yes | Yes | Yes | Yes | No | Yes | Yes | n.a. | No | No | Yes | n.r. | Yes | Yes | Fair |
| Pan Y, 2017 | Yes | Yes | No | Yes | No | Yes | Yes | n.a. | Yes | No | Yes | Yes | Yes | Yes | Good |
| Jing, 2017 | Yes | Yes | Yes | Yes | Yes | Yes | Yes | Yes | Yes | No | Yes | Yes | Yes | Yes | Good |
| Pan, 2017 | Yes | Yes | Yes | Yes | No | Yes | Yes | Yes | Yes | No | Yes | Yes | Yes | Yes | Good |
| Stefanovic, 2017 | Yes | Yes | Yes | Yes | No | Yes | Yes | n.a. | No | No | Yes | n.r. | n.r. | Yes | Fair |
| Pirinen, 2017 | Yes | Yes | Yes | Yes | No | Yes | Yes | n.a. | Yes | No | Yes | n.r. | Yes | Yes | Good |
| Corrao, 2017 | Yes | Yes | Yes | Yes | No | Yes | Yes | n.r. | Yes | No | Yes | n.r. | Yes | Yes | Good |
| Yoo J, 2017 | Yes | No | Yes | Yes | No | Yes | Yes | n.a. | Yes | No | Yes | n.r. | Yes | Yes | Good |
| Pan, 2016 | Yes | Yes | Yes | Yes | Yes | Yes | Yes | Yes | Yes | No | Yes | Yes | Yes | Yes | Good |
| Jing, 2016 | Yes | Yes | Yes | Yes | No | Yes | Yes | Yes | Yes | No | Yes | No | Yes | Yes | Good |
| Pan, 2016 | Yes | Yes | Yes | Yes | No | Yes | Yes | Yes | Yes | No | Yes | n.r. | Yes | Yes | Good |
| Kang, 2016 | Yes | Yes | Yes | Yes | No | Yes | Yes | n.a. | n.r. | No | Yes | n.r. | Yes | Yes | Good |
| Wood, 2016 | Yes | Yes | Yes | Yes | No | Yes | Yes | n.a. | Yes | No | Yes | No | Yes | Yes | Good |
| Policardo, 2015 | Yes | Yes | Yes | Yes | No | Yes | Yes | n.a. | Yes | No | Yes | No | Yes | Yes | Good |
| Cruz-Herranz, 2015 | Yes | Yes | n.r. | Yes | No | Yes | Yes | Yes | Yes | No | Yes | n.r. | Yes | Yes | Fair |
| Wu, 2015 | Yes | Yes | Yes | Yes | No | Yes | Yes | n.a. | Yes | No | Yes | Yes | Yes | Yes | Good |
| Zhang C, 2015 | Yes | Yes | Yes | Yes | No | Yes | Yes | n.a. | Yes | No | Yes | Yes | Yes | Yes | Good |
| Li B, 2015 | Yes | Yes | Yes | Yes | No | Yes | Yes | n.a. | Yes | No | Yes | n.r. | Yes | Yes | Good |
| Muñoz-Rivas, 201 | Yes | Yes | Yes | Yes | No | Yes | Yes | n.a. | Yes | No | No | No | n.r. | Yes | Fair |
| Policardo, 2015 | Yes | Yes | Yes | Yes | No | Yes | Yes | n.a. | Yes | No | Yes | No | Yes | Yes | Good |
| Masrur, 2015 | Yes | Yes | Yes | Yes | No | Yes | Yes | Yes | Yes | No | Yes | n.r. | Yes | Yes | Good |
| Serena, 2015 | Yes | Yes | Yes | Yes | No | Yes | Yes | n.a. | Yes | No | n.r. | n.r. | Yes | Yes | Fair |
| Lin J, 2015 | Yes | Yes | Yes | Yes | No | Yes | Yes | n.a. | Yes | No | Yes | Yes | Yes | Yes | Good |
| Rutten-Jacobs, 2014 | Yes | Yes | No | Yes | No | Yes | Yes | Yes | Yes | Yes | Yes | n.r. | Yes | Yes | Fair |
| Jia, 2014 | Yes | Yes | Yes | Yes | No | Yes | Yes | n.a. | Yes | No | Yes | No | Yes | Yes | Good |
| Toni, 2014 | Yes | Yes | Yes | Yes | No | Yes | Yes | n.a. | Yes | No | Yes | Yes | Yes | Yes | Good |
| Pezzini A, 2014 | Yes | Yes | Yes | Yes | No | Yes | Yes | n.a. | Yes | No | Yes | n.r. | Yes | Yes | Good |
| Wu, 2014 | Yes | Yes | Yes | Yes | No | Yes | Yes | Yes | Yes | No | Yes | Yes | Yes | Yes | Good |
| Roquer, 2014 | Yes | Yes | Yes | Yes | No | Yes | Yes | Yes | Yes | No | Yes | Yes | Yes | Yes | Good |
| Hjalmarsson, 2014 | Yes | Yes | Yes | Yes | No | Yes | Yes | Yes | Yes | No | Yes | n.r. | n.r. | Yes | Fair |
| Arnold, 2014 | Yes | Yes | Yes | Yes | No | Yes | Yes | Yes | Yes | No | Yes | n.r. | Yes | Yes | Good |
| Zhao, 2014 | Yes | Yes | Yes | Yes | No | Yes | Yes | n.a. | Yes | No | Yes | Yes | Yes | Yes | Good |
| Aarnio, 2014 | Yes | Yes | Yes | Yes | No | Yes | Yes | n.a. | Yes | No | Yes | n.r. | Yes | Yes | Good |
| Erdur, 2014 | Yes | Yes | Yes | Yes | No | Yes | Yes | n.a. | Yes | No | Yes | n.r. | Yes | Yes | Good |
| Rutten-Jacobs, 2013 | Yes | Yes | n.r. | Yes | No | Yes | Yes | n.a. | Yes | No | Yes | n.r. | n.r. | Yes | Fair |
| Wang, 2013 | Yes | Yes | Yes | Yes | No | Yes | Yes | n.a. | Yes | No | Yes | Yes | Yes | NA | Good |
| Wang, 2013 | Yes | Yes | Yes | Yes | No | Yes | Yes | No | Yes | No | Yes | n.r. | Yes | Yes | Good |
| Rocco, 2013 | Yes | Yes | n.r. | Yes | No | Yes | Yes | Yes | Yes | No | Yes | n.r. | n.r. | Yes | Fair |
| Gomes, 2013 | Yes | Yes | Yes | Yes | No | Yes | Yes | n.a. | Yes | No | Yes | n.r. | Yes | Yes | Good |
| Hanchate, 2013 | Yes | Yes | Yes | Yes | No | Yes | Yes | n.a. | Yes | No | Yes | n.r. | Yes | Yes | Good |
| Balti, 2013 | Yes | Yes | Yes | Yes | Yes | Yes | Yes | Yes | Yes | No | Yes | n.r. | Yes | Yes | Good |
| Tsivgoulis, 2012 | Yes | Yes | Yes | Yes | No | Yes | Yes | n.a. | Yes | No | Yes | Yes | Yes | Yes | Good |
| Purroy, 2012 | Yes | Yes | Yes | Yes | No | Yes | Yes | n.a. | Yes | No | Yes | n.r. | Yes | Yes | Good |
| Fuentes, 2012 | Yes | Yes | Yes | Yes | No | Yes | Yes | Yes | Yes | No | Yes | No | Yes | Yes | Good |
| Li, 2011 | Yes | Yes | No | Yes | No | Yes | Yes | n.a. | Yes | No | Yes | No | Yes | Yes | Fair |
| Jia, 2011 | Yes | Yes | Yes | Yes | No | Yes | Yes | n.a. | Yes | No | Yes | Yes | Yes | Yes | Good |
| Hassan, 2011 | Yes | Yes | Yes | Yes | No | Yes | Yes | n.a. | Yes | No | Yes | No | Yes | Yes | Good |
| Andersen, 2011 | Yes | Yes | Yes | Yes | No | Yes | Yes | n.a. | Yes | No | Yes | No | Yes | Yes | Good |
| Greisenegger, 2011 | Yes | Yes | Yes | Yes | No | Yes | Yes | n.a. | Yes | No | Yes | n.r. | Yes | Yes | Good |
| Burns, 2011 | Yes | Yes | Yes | Yes | No | Yes | Yes | n.a. | n.r. | No | Yes | n.r. | Yes | Yes | Fair |
| Winell, 2011 | Yes | No | Yes | No | No | Yes | Yes | n.a. | Yes | No | Yes | No | Yes | Yes | Poor |
| Putaala, 2010 | Yes | Yes | Yes | Yes | No | Yes | Yes | n.a. | Yes | No | Yes | Yes | Yes | Yes | Good |
| Nakajima, 2010 | Yes | Yes | Yes | Yes | No | Yes | Yes | n.a. | No | No | Yes | n.r. | Yes | Yes | Fair |
| Reeves, 2010 | Yes | Yes | Yes | Yes | No | Yes | Yes | n.a. | Yes | No | Yes | n.r. | Yes | Yes | Good |
| Wu, 2010 | Yes | Yes | Yes | Yes | No | Yes | Yes | n.a. | Yes | No | Yes | n.r. | Yes | Yes | Good |
| Hassan, 2010 | Yes | Yes | Yes | Yes | No | Yes | Yes | n.a. | Yes | No | Yes | No | Yes | Yes | Good |
| Meurer, 2010 | Yes | Yes | Yes | Yes | No | Yes | Yes | No | Yes | No | Yes | Yes | n.r. | Yes | Good |
| Ahmed, 2010 | Yes | Yes | Yes | Yes | No | Yes | Yes | Yes | Yes | No | Yes | n.r. | n.r. | Yes | Fair |
| Fonarow, 2010 | Yes | Yes | Yes | Yes | Yes | Yes | Yes | n.a. | No | No | Yes | n.r. | Yes | Yes | Fair |
| Smith, 2010 | Yes | Yes | Yes | Yes | Yes | Yes | Yes | n.a. | No | No | Yes | n.r. | Yes | Yes | Fair |
| Koton S, 2010 | Yes | Yes | Yes | Yes | No | Yes | Yes | n.a. | No | No | Yes | n.r. | Yes | Yes | Fair |
| Putaala J, 2010 | Yes | Yes | Yes | Yes | No | Yes | Yes | Yes | Yes | Yes | Yes | n.r. | Yes | Yes | Good |
| Spengos K, 2010 | Yes | Yes | Yes | Yes | Yes | Yes | Yes | n.a. | Yes | No | Yes | n.r. | Yes | Yes | Good |
| Sun, 2009 | Yes | Yes | Yes | No | No | Yes | Yes | n.a. | Yes | No | Yes | No | Yes | Yes | Fair |
| Yokota, 2009 | Yes | Yes | Yes | Yes | Yes | Yes | Yes | No | Yes | No | Yes | No | Yes | Yes | Good |
| Gulli, 2009 | Yes | Yes | Yes | Yes | No | Yes | Yes | Yes | Yes | No | Yes | n.r. | Yes | Yes | Good |
| Oksala, 2009 | Yes | Yes | No | Yes | No | Yes | Yes | n.a. | Yes | No | Yes | No | Yes | Yes | Poor |
| Putaala, 2009 | Yes | Yes | n.r. | Yes | No | Yes | Yes | Yes | Yes | No | Yes | n.r. | n.r. | Yes | Fair |
| Busch, 2009 | Yes | Yes | Yes | Yes | Yes | Yes | Yes | n.a. | Yes | No | Yes | n.r. | Yes | Yes | Good |
| Kamalesh, 2008 | Yes | Yes | Yes | Yes | No | Yes | Yes | n.a. | Yes | No | Yes | No | n.r. | Yes | Fair |
| Gunarathne, 2008 | Yes | Yes | Yes | Yes | No | Yes | Yes | n.a. | Yes | No | Yes | No | Yes | Yes | Good |
| Staals, 2008 | Yes | Yes | Yes | Yes | No | Yes | Yes | n.a. | Yes | No | Yes | Yes | Yes | Yes | Good |
| Toyoda, 2007 | Yes | Yes | Yes | Yes | No | Yes | Yes | n.a. | Yes | No | Yes | n.r. | Yes | Yes | Good |
| Han, 2007 | Yes | Yes | Yes | Yes | Yes | Yes | Yes | n.a. | Yes | No | Yes | Yes | Yes | Yes | Good |
| Johnston, 2007 | Yes | Yes | n.r. | Yes | No | Yes | Yes | n.a. | No | No | Yes | n.r. | Yes | Yes | Fair |
| Prosser, 2007 | Yes | Yes | Yes | Yes | No | Yes | Yes | n.a. | Yes | No | Yes | No | Yes | Yes | Good |
| Roquer, 2007 | Yes | Yes | Yes | Yes | No | Yes | Yes | Yes | Yes | No | Yes | No | Yes | Yes | Good |
| Vermeer, 2006 | Yes | Yes | Yes | Yes | No | Yes | Yes | Yes | Yes | No | Yes | n.r. | Yes | Yes | Good |
| Xu, 2006 | Yes | Yes | Yes | Yes | No | Yes | Yes | Yes | Yes | No | n.r. | n.r. | Yes | Yes | Fair |
| Tsivgoulis, 2006 | Yes | Yes | Yes | Yes | No | Yes | Yes | n.a. | Yes | No | Yes | Yes | Yes | Yes | Good |
| Kammersgaard, 2006 | Yes | Yes | Yes | Yes | No | Yes | Yes | n.a. | Yes | No | Yes | n.r. | Yes | Yes | Good |
| Sacco, 2006 | Yes | Yes | Yes | Yes | No | Yes | Yes | n.a. | No | No | Yes | n.r. | Yes | Yes | Fair |
| Hamidon, 2006 | Yes | Yes | Yes | Yes | Yes | Yes | Yes | n.a. | n.r. | No | n.r. | n.r. | Yes | Yes | Poor |
| Mateo, 2006 | Yes | Yes | Yes | Yes | No | Yes | Yes | n.a. | Yes | No | Yes | n.r. | Yes | Yes | Good |
| Bateman, 2006 | Yes | Yes | Yes | Yes | No | Yes | Yes | n.a. | Yes | No | Yes | n.r. | Yes | Yes | Good |
| van Wijk, 2005 | Yes | Yes | Yes | Yes | No | Yes | Yes | n.a. | Yes | No | Yes | No | Yes | Yes | Good |
| Fu, 2005 | Yes | Yes | Yes | Yes | No | Yes | Yes | n.a. | Yes | No | Yes | n.r. | Yes | Yes | Good |
| Rothwell, 2005 | Yes | Yes | Yes | Yes | Yes | Yes | Yes | n.a. | Yes | No | Yes | n.r. | Yes | Yes | Good |
| Kaplan, 2005 | Yes | Yes | Yes | Yes | No | Yes | Yes | n.a. | Yes | No | Yes | n.r. | Yes | Yes | Good |
| Kaarisalo, 2005 | Yes | Yes | Yes | Yes | No | Yes | Yes | n.a. | Yes | No | Yes | n.r. | Yes | Yes | Good |
| Kimura, 2005 | Yes | Yes | Yes | Yes | No | Yes | Yes | n.a. | Yes | No | Yes | n.r. | No | Yes | Fair |
| Soda, 2004 | Yes | Yes | Yes | Yes | No | Yes | Yes | n.a. | Yes | No | Yes | n.r. | Yes | Yes | Good |
| Lee, 2004 | Yes | Yes | Yes | Yes | No | Yes | Yes | n.a. | n.r. | No | Yes | No | Yes | Yes | Good |
| Varona, 2004 | Yes | Yes | n.r. | Yes | No | Yes | Yes | n.a. | No | No | Yes | n.r. | Yes | Yes | Poor |
| Hill, 2004 | Yes | Yes | Yes | Yes | No | Yes | Yes | n.a. | No | No | Yes | n.r. | Yes | Yes | Fair |
| Heuschmann, 2004 | Yes | Yes | Yes | Yes | No | Yes | Yes | n.a. | Yes | No | Yes | n.r. | Yes | Yes | Good |
| Hamidon, 2003 | Yes | Yes | Yes | Yes | No | Yes | Yes | Yes | Yes | No | Yes | No | Yes | n.r. | Fair |
| Conway, 2003 | Yes | Yes | Yes | Yes | No | Yes | Yes | n.a. | Yes | No | Yes | No | Yes | Yes | Good |
| Lee, 2003 | Yes | Yes | Yes | Yes | No | Yes | Yes | n.a. | Yes | No | Yes | n.r. | Yes | Yes | Good |
| De Jong, 2003 | Yes | Yes | Yes | Yes | No | Yes | Yes | n.a. | Yes | No | Yes | n.r. | Yes | Yes | Good |
| Wong, 2003 | Yes | Yes | Yes | Yes | No | Yes | Yes | n.a. | Yes | No | Yes | n.r. | Yes | Yes | Good |
| De Jong, 2002 | Yes | Yes | Yes | Yes | No | Yes | Yes | n.a. | Yes | No | Yes | Yes | Yes | Yes | Good |
| Petty, 2000 | Yes | Yes | Yes | Yes | No | Yes | Yes | n.a. | Yes | No | Yes | n.r. | Yes | Yes | Good |
| Johnston, 2000 | Yes | Yes | Yes | Yes | No | Yes | Yes | n.a. | No | No | Yes | Yes | Yes | Yes | Fair |
| Wong, 2000 | Yes | Yes | Yes | Yes | No | Yes | Yes | n.a. | No | No | Yes | n.r. | Yes | Yes | Fair |
| Wong, 1999 | Yes | Yes | Yes | Yes | No | Yes | Yes | n.a. | Yes | No | Yes | n.r. | Yes | Yes | Good |
| Marini, 1999 | Yes | Yes | Yes | Yes | No | Yes | Yes | n.a. | Yes | No | Yes | n.r. | Yes | Yes | Good |
| Samsa, 1999 | Yes | Yes | Yes | Yes | No | Yes | Yes | n.a. | No | No | Yes | n.r. | Yes | Yes | Fair |
| Clavier, 1994 | Yes | Yes | Yes | Yes | No | Yes | Yes | n.a. | Yes | No | Yes | n.r. | Yes | Yes | Good |
| Hier, 1991 | Yes | Yes | Yes | Yes | No | Yes | Yes | n.a. | n.r. | No | Yes | n.r. | Yes | Yes | Good |
| Howard G, 1989 | Yes | No | Yes | Yes | No | Yes | Yes | n.a. | No | No | No | n.r. | n.r. | Yes | Poor |
| Howard, 1987 | Yes | n.r. | Yes | Yes | No | n.a. | Yes | n.a. | n.r. | No | Yes | No | Yes | Yes | Poor |

QOE, Quality of Evidence; n.a.: not applicable; n.r.: not reported.

Q1: Was the research question or objective in this paper clearly stated?

Q2. Was the study population clearly specified and defined?

Q3. Was the participation rate of eligible persons at least 50%?

Q4. Were all the subjects selected or recruited from the same or similar populations (including the same time period)? Were inclusion and exclusion criteria for being in the study prespecified and applied uniformly to all participants?

Q5. Was a sample size justification, power description, or variance and effect estimates provided?

Q6. For the analyses in this paper, were the exposure(s) of interest measured prior to the outcome(s) being measured?

Q7. Was the timeframe sufficient so that one could reasonably expect to see an association between exposure and outcome if it existed?

Q8. For exposures that can vary in amount or level, did the study examine different levels of the exposure as related to the outcome (e.g., categories of exposure, or exposure measured as continuous variable)?

Q9. Were the exposure measures (independent variables) clearly defined, valid, reliable, and implemented consistently across all study participants?

Q10. Was the exposure(s) assessed more than once over time?

Q11. Were the outcome measures (dependent variables) clearly defined, valid, reliable, and implemented consistently across all study participants?

Q12. Were the outcome assessors blinded to the exposure status of participants?

Q13. Was loss to follow-up after baseline 20% or less?

Q14. Were key potential confounding variables measured and adjusted statistically for their impact on the relationship between exposure(s) and outcome(s)

# Supplementary figure 2: Funnel plot for the outcome composite vascular events and diabetes mellitus


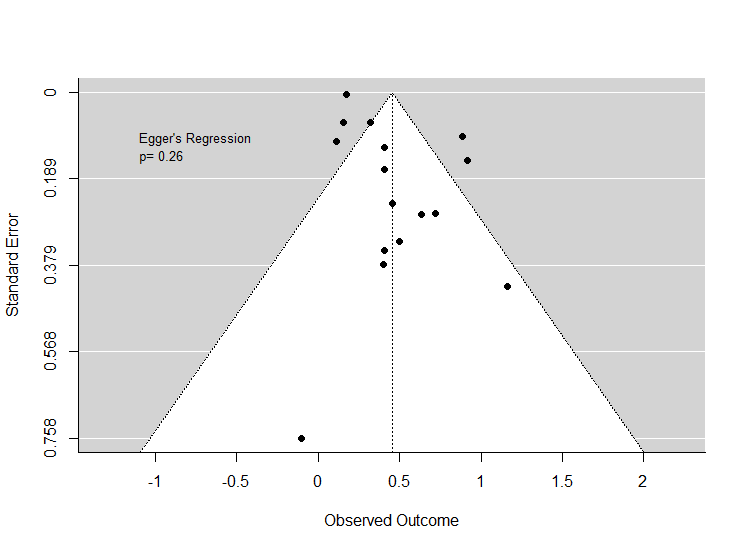


# Supplementary figure 3: Funnel plot for the outcome recurrent stroke and diabetes mellitus


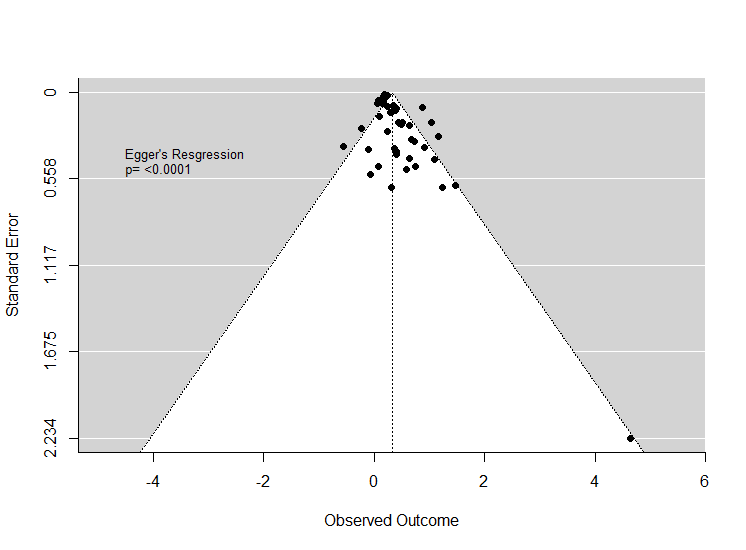


# Supplementary figure 4: Forest plot for the outcome recurrent stroke and diabetes mellitus in odds ratios


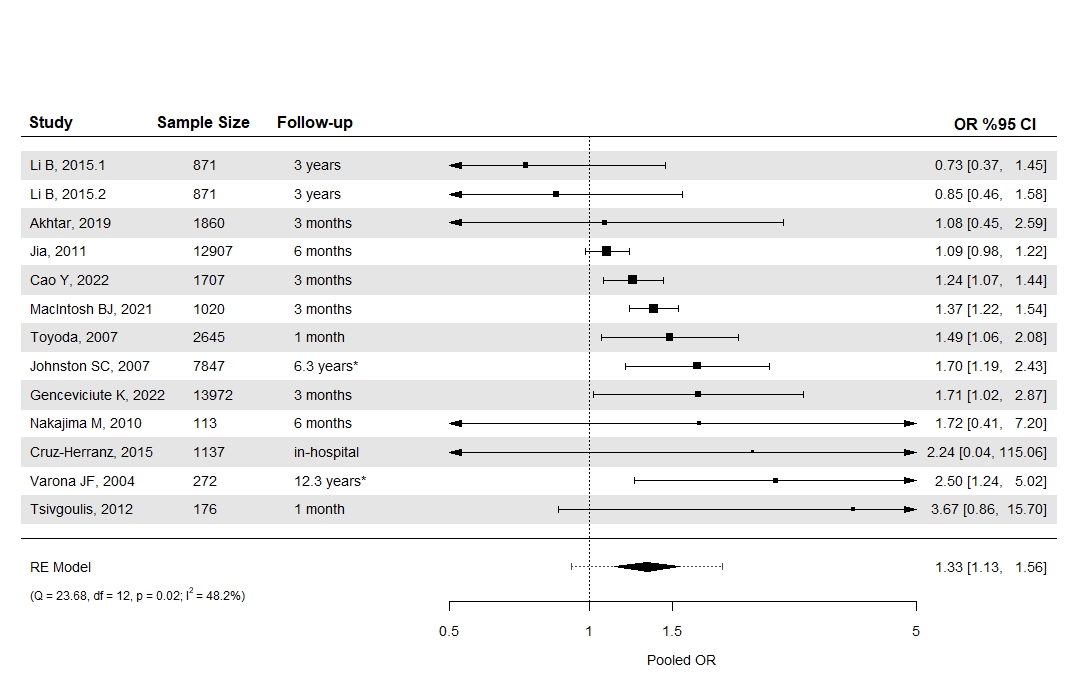


Follow-up time is given as maximum. * denotes mean and ** denotes median follow-up. The edges of the diamond represent confidence interval and the bars represent prediction interval.

# Supplementary figure 5: Forest plot for subgroup analysis of the outcome recurrent stroke and diabetes mellitus

Subgroups based on baseline event (TIA/IS/both).


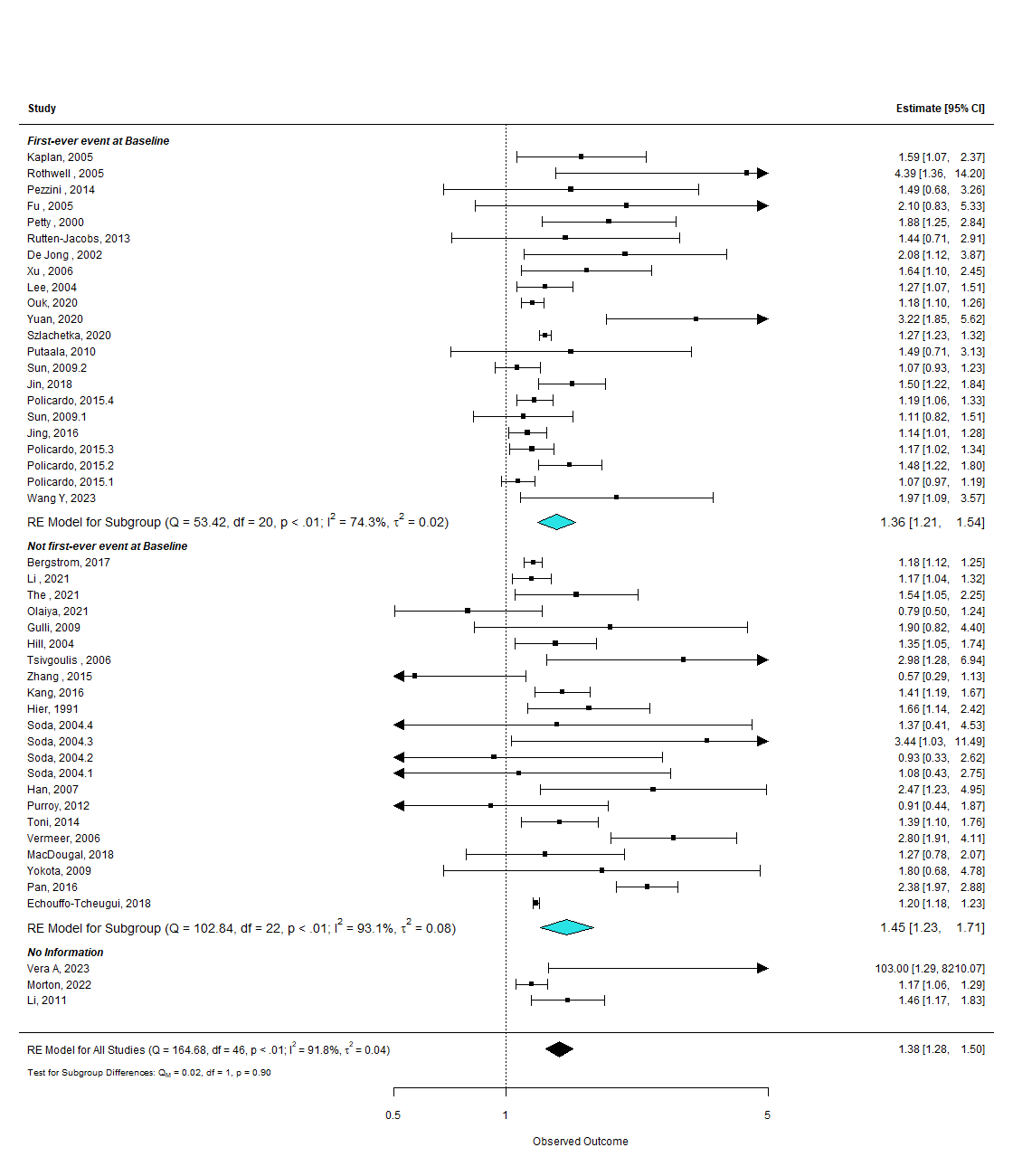


Follow-up time is given as maximum. * denotes mean and ** denotes median follow-up.

Subgroups based on baseline event (TIA/IS/both).


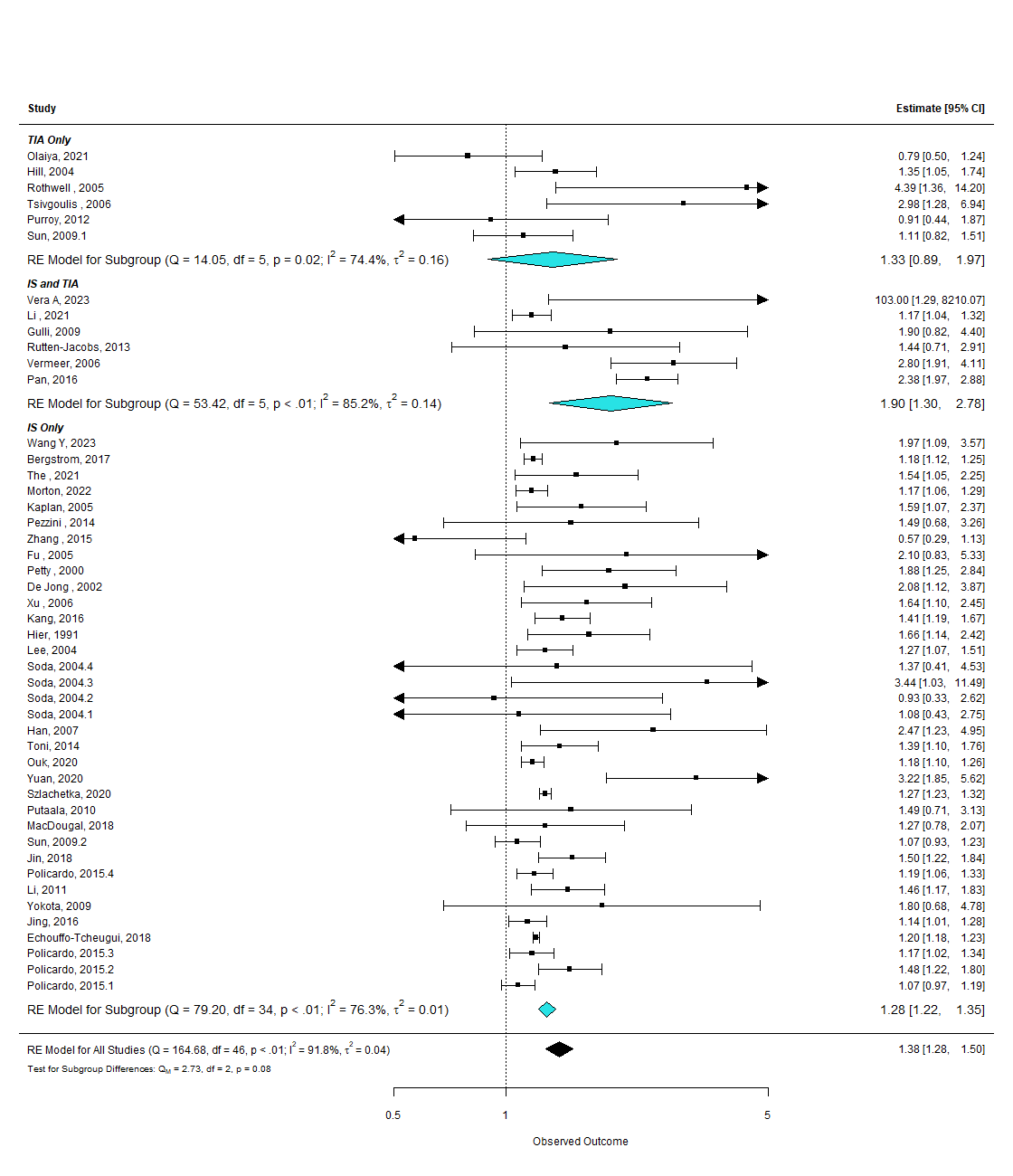


Follow-up time is given as maximum. * denotes mean and ** denotes median follow-up.

#
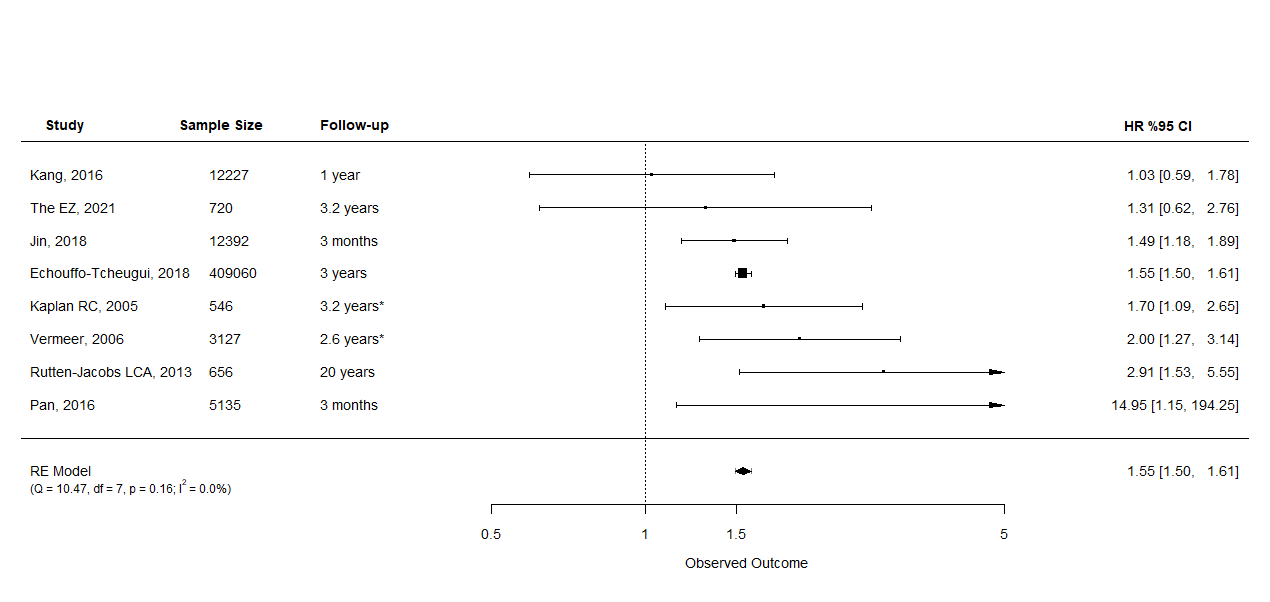
Supplementary figure 6: Forest plot for the outcome cardiac events and diabetes mellitus

Follow-up time is given as maximum. * denotes mean and ** denotes median follow-up. The edges of the diamond represent confidence interval and the bars represent prediction interval.

# Supplementary figure 7: Forest plot for the outcome cardiovascular mortality and diabetes mellitus


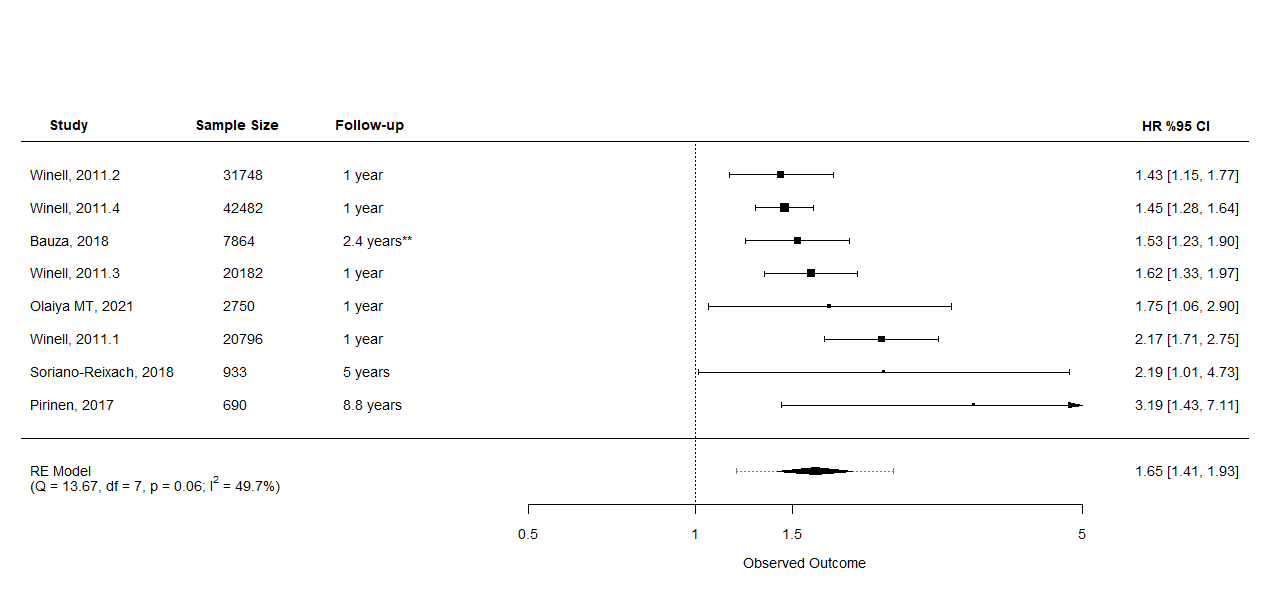


Follow-up time is given as maximum. * denotes mean and ** denotes median follow-up. The edges of the diamond represent confidence interval and the bars represent prediction interval.

# Supplementary figure 8: Forest plot for subgroup analysis of the outcome all-cause mortality and diabetes mellitus


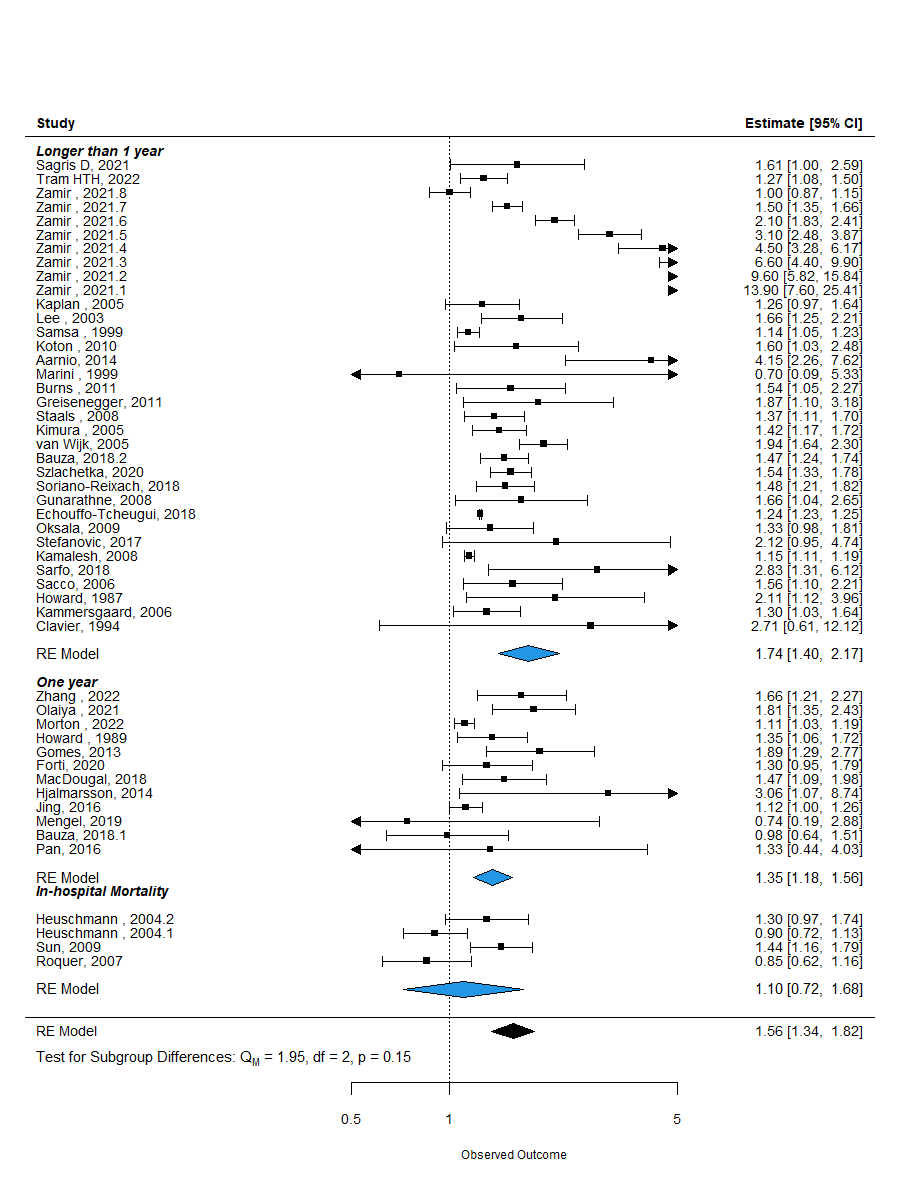


# Supplementary figure 9: Galbraith plot for the outcome all-cause mortality and exposure diabetes mellitus


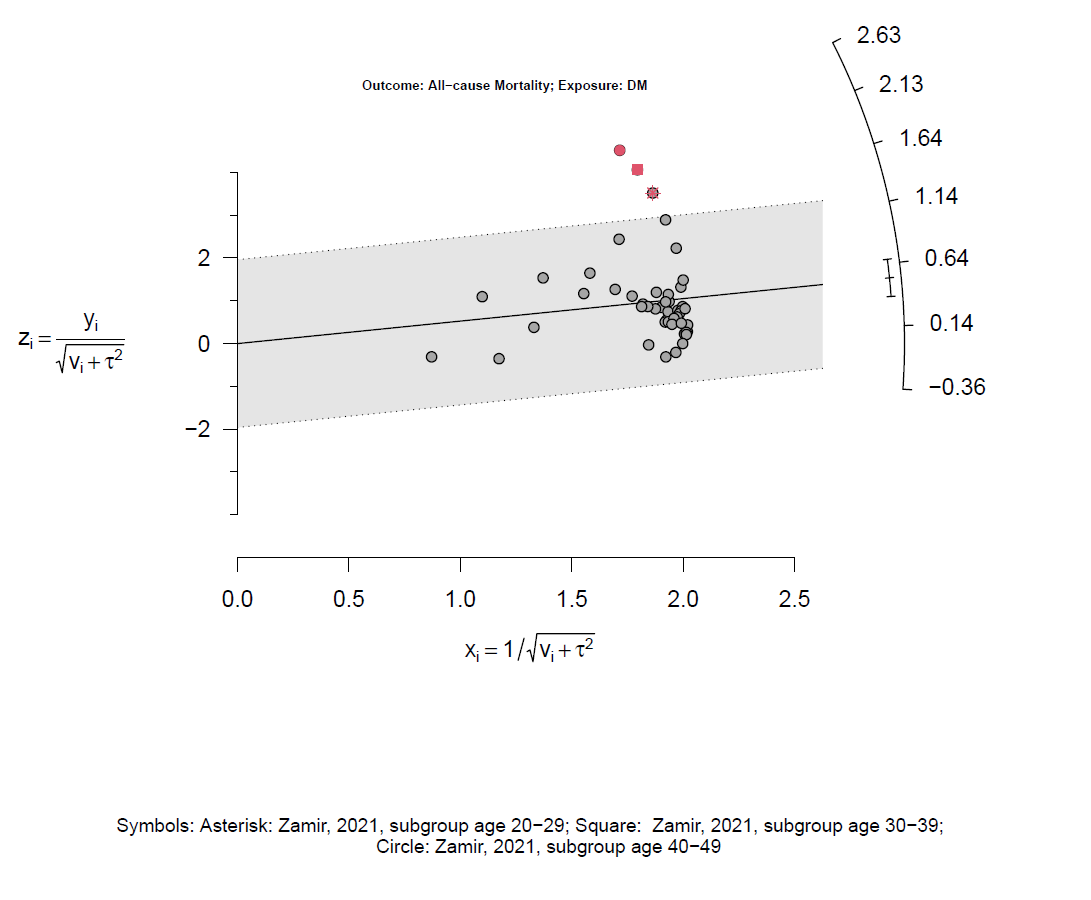


# Supplementary figure 10: Forest plot for the outcome all-cause mortality and diabetes mellitus in odds ratios


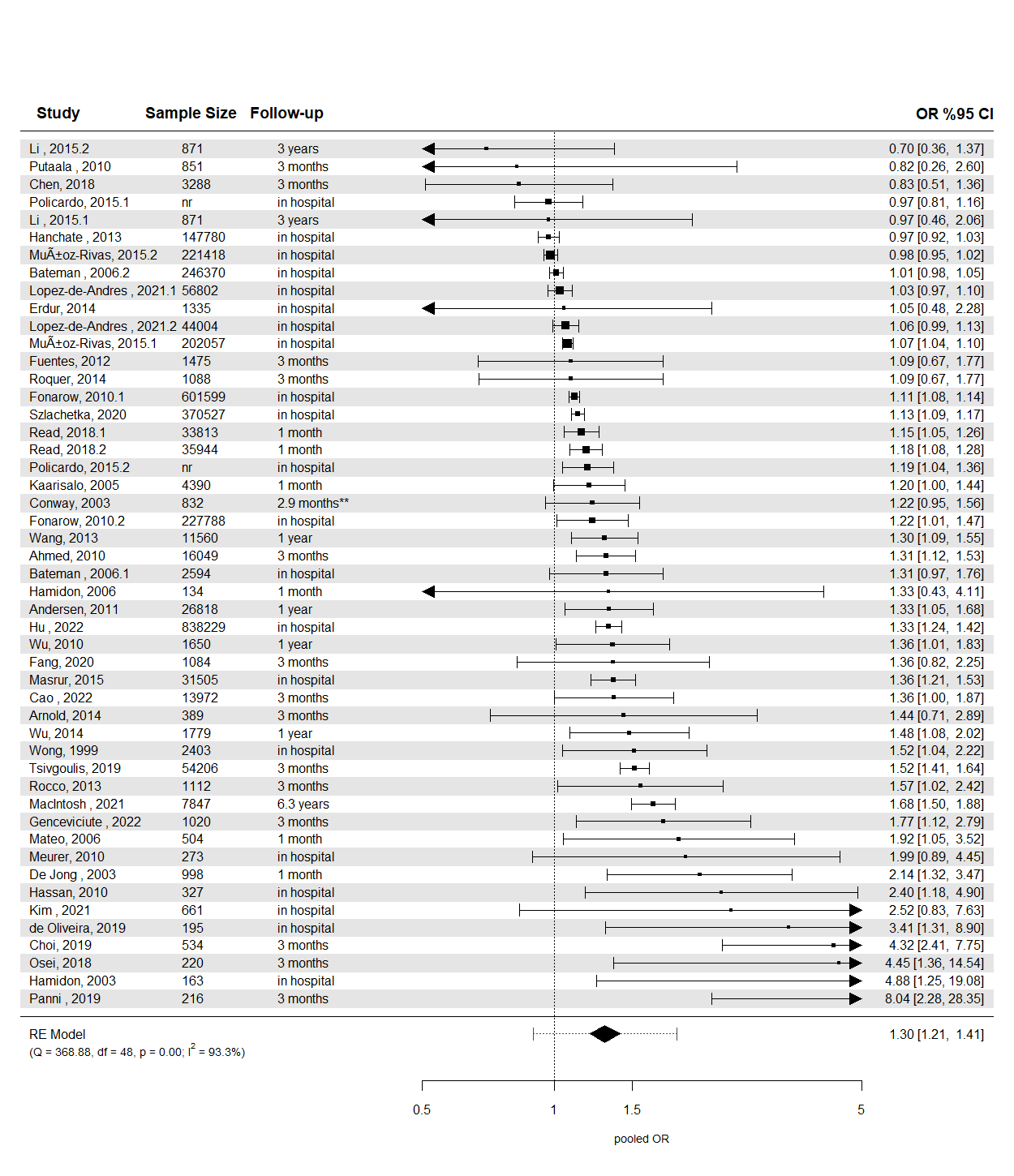


Follow-up time is given as maximum. * denotes mean and ** denotes median follow-up. The edges of the diamond represent confidence interval and the bars represent prediction interval.

# Supplementary figure 11: Funnel plots for the outcome all-cause mortality diabetes mellitus


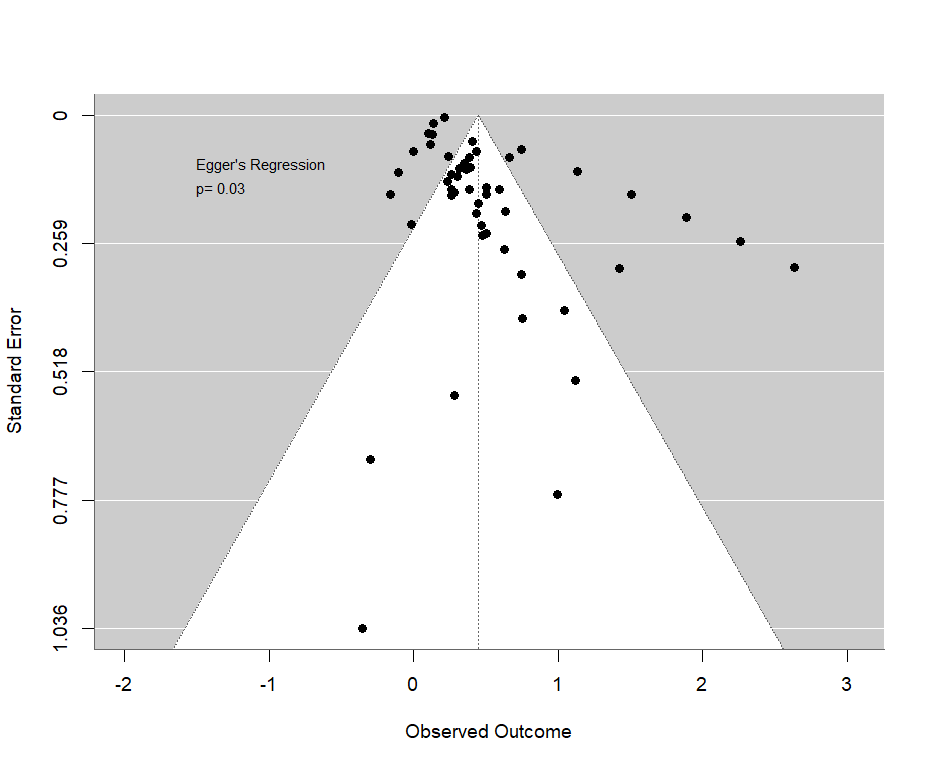
A) Hazard Ratios

B) Odds Ratios


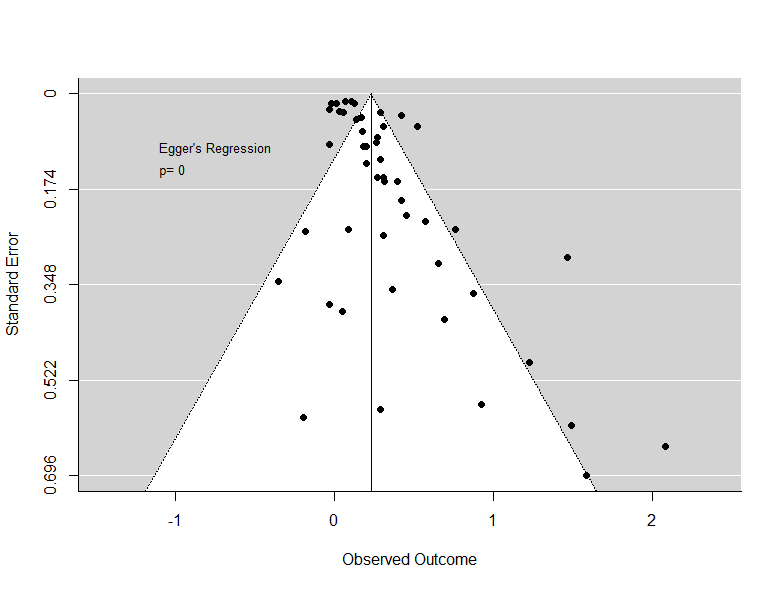


# Supplementary figure 12: Forest plot for the outcome all-cause mortality and prediabetes


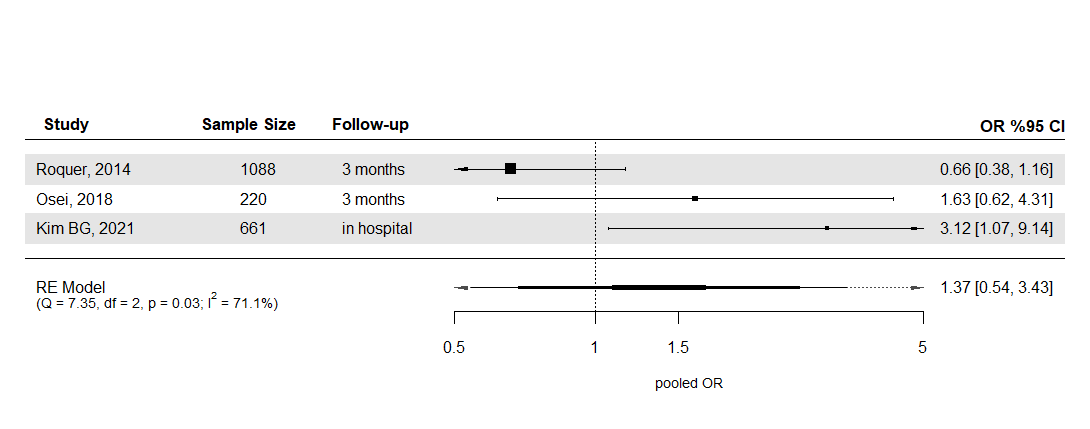


Follow-up time is given as maximum. The edges of the diamond represent confidence interval and the bars represent prediction interval.

# Supplementary Figure 13: Adjustment Factors of the Studies Included in the Review


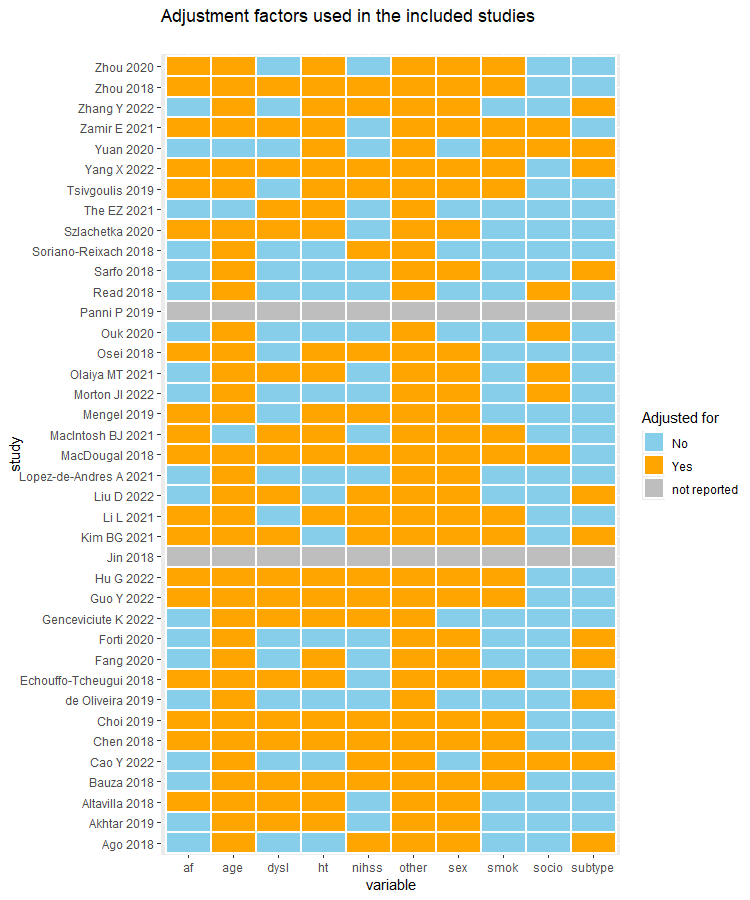


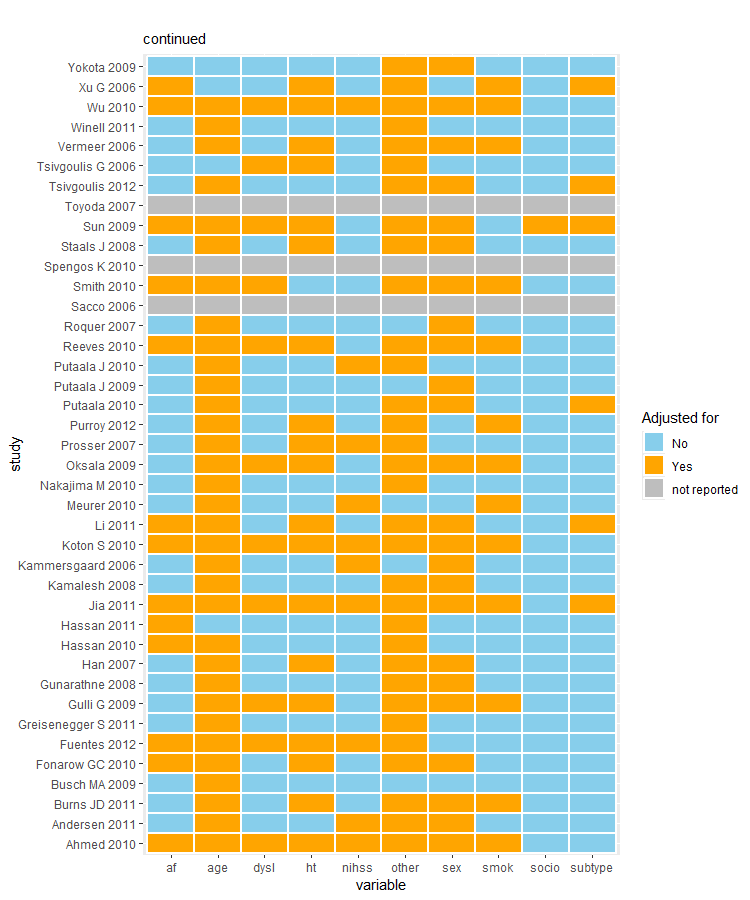

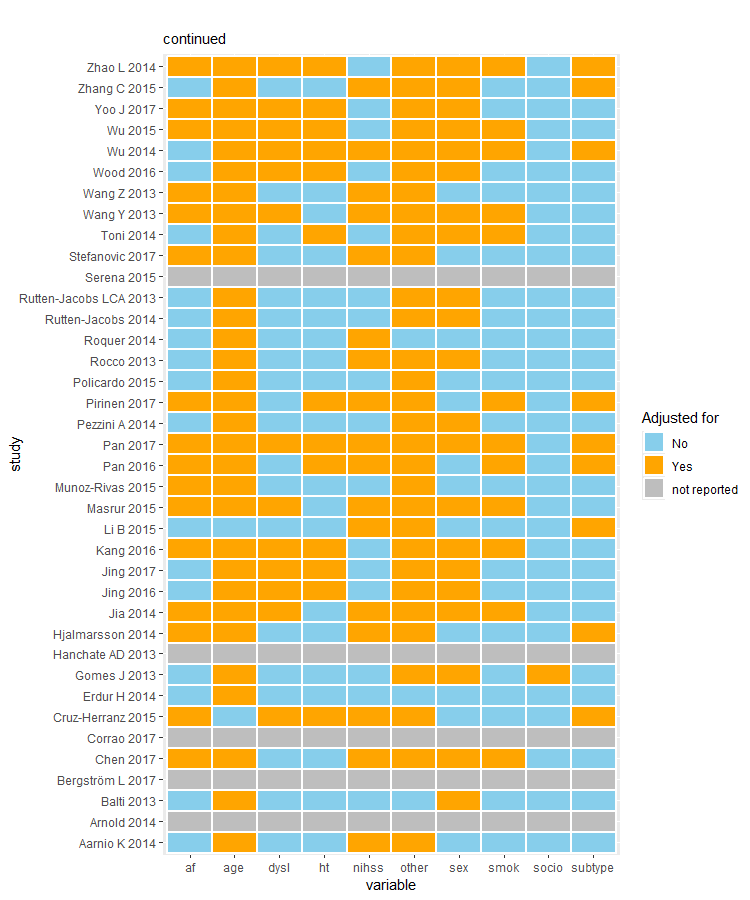


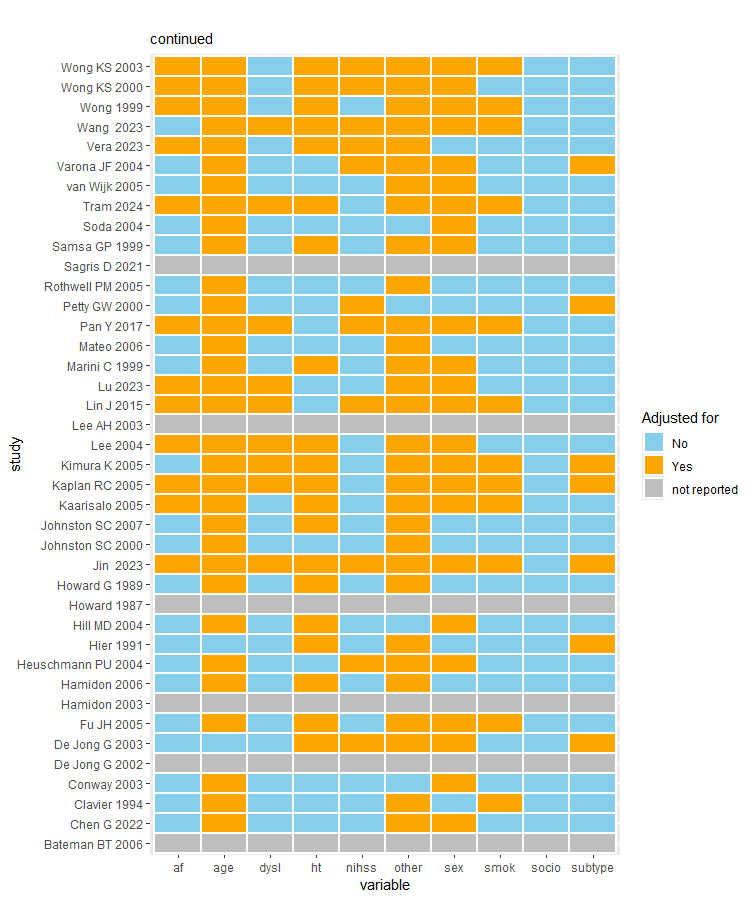


Adjustment factors denote: af: atrial fibrillation; dysl: dyslipidemia including hypercholesterolemia, hypertriglyceridemia, blood lipid levels; ht: hypertension, blood pressure levels; nihss: NIH Stroke scale; other: other adjustment factors as listed here; sex: female/male; smok: history of or active smoking; socio: socioeconomic status and/or education status; subtype: stroke etiology and/or subtype.

#
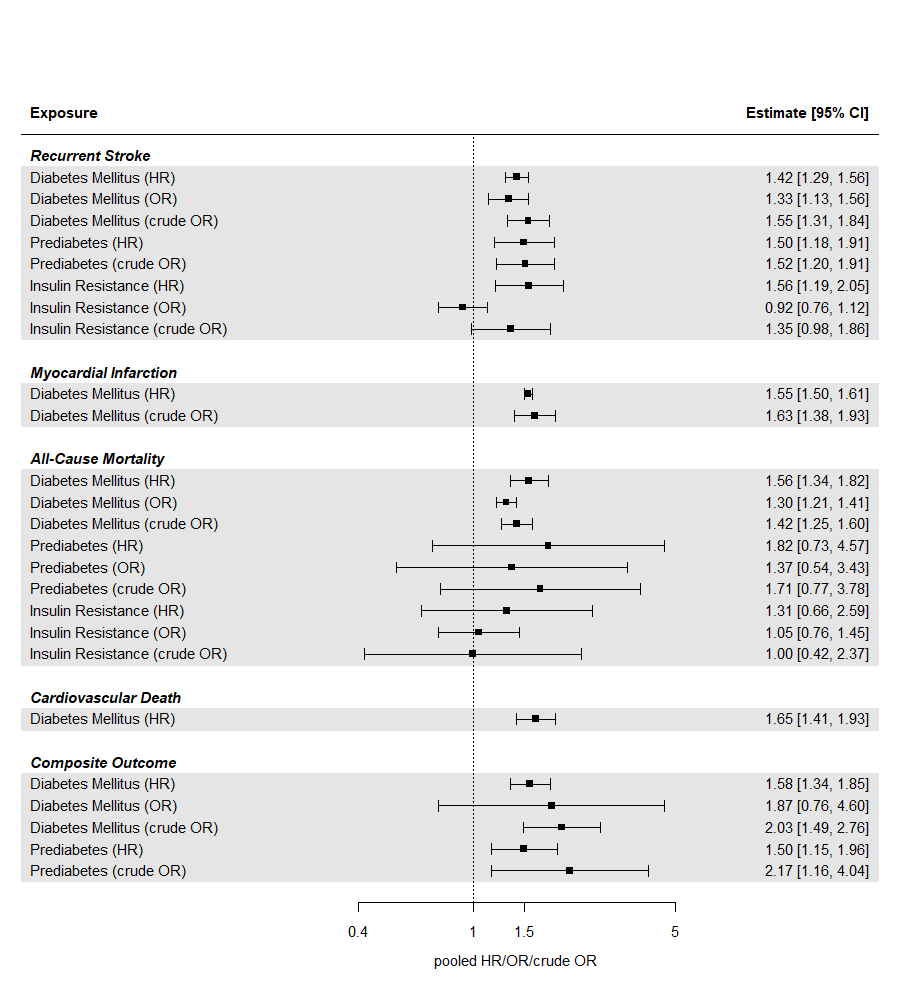
Supplementary figure 14: Crude odds ratios for all outcomes

# Supplementary References

1. Lu Z, Xiong Y, Feng X, et al. Insulin resistance estimated by estimated glucose disposal rate predicts outcomes in acute ischemic stroke patients. *Cardiovasc Diabetol*. 2023;22(1):225. doi:10.1186/s12933-023-01925-1

2. Jin A, Wang S, Li J, et al. Mediation of Systemic Inflammation on Insulin Resistance and Prognosis of Nondiabetic Patients With Ischemic Stroke. *Stroke*. 2023;54(3):759-769. doi:10.1161/STROKEAHA.122.039542

3. Tram HTH, Tanaka-Mizuno S, Takashima N, et al. Control of Diabetes Mellitus and Long-Term Prognosis in Stroke Patients: The Shiga Stroke and Heart Attack Registry. *Cerebrovascular Diseases*. 2023;52(1):81-88. doi:10.1159/000525648

4. Vera A, Cecconi A, Ximénez-Carrillo Á, et al. Risk of recurrent stroke and mortality after cryptogenic stroke in diabetic patients. *Heart Vessels*. 2023;38(6):817-824. doi:10.1007/s00380-023-02235-y

5. Wang Y, Fan H, Duan W, et al. Elevated stress hyperglycemia and the presence of intracranial artery stenosis increase the risk of recurrent stroke. *Front Endocrinol (Lausanne)*. 2023;13. doi:10.3389/fendo.2022.954916

6. Cao Y, Chen Y, Zhang X, Wang Y. Analysis of Prognostic Risk Factors for Ischemic Stroke in China: A Multicentre Retrospective Clinical Study; A National Survey in China. *Curr Neurovasc Res*. 2022;19(1):117-126. doi:10.2174/1567202619666220331160024

7. Genceviciute K, Göldlin MB, Kurmann CC, et al. Association of diabetes mellitus and admission glucose levels with outcome after endovascular therapy in acute ischaemic stroke in anterior circulation. *Eur J Neurol*. 2022;29(10):2996-3008. doi:10.1111/ene.15456

8. Guo Y, Wang G, Jing J, et al. Stress hyperglycemia may have higher risk of stroke recurrence than previously diagnosed diabetes mellitus. *Aging*. 2021;13(6):9108-9118. doi:10.18632/aging.202797

9. Hu G, Gu H, Jiang Y, et al. Prevalence and In-hospital outcomes of diabetes among acute ischemic stroke patients in china: results from the Chinese Stroke Center Alliance. *J Neurol*. 2022;269(9):4772-4782. doi:10.1007/s00415-022-11112-z

10. Liu D, Yang K, Gu H, Li Z, Wang Y, Wang Y. Predictive effect of triglyceride-glucose index on clinical events in patients with acute ischemic stroke and type 2 diabetes mellitus. *Cardiovasc Diabetol*. 2022;21(1). doi:10.1186/s12933-022-01704-4

11. Morton JI, Ilomäki J, Wood SJ, Bell JS, Shaw JE, Magliano DJ. One-year readmission and mortality following ischaemic stroke by diabetes status, sex, and socioeconomic disadvantage: An analysis of 27,802 strokes from 2012 to 2017. *J Neurol Sci*. 2022;434. doi:10.1016/j.jns.2022.120149

12. Yang X, Wang G, Jing J, et al. Association of triglyceride-glucose index and stroke recurrence among nondiabetic patients with acute ischemic stroke. *BMC Neurol*. 2022;22(1). doi:10.1186/s12883-022-02588-3

13. Zhang Y, Jin A, Meng X, et al. Association between diabetes duration and 1-year prognosis of stroke: A national registry study. *Brain Behav*. 2022;12(9). doi:10.1002/brb3.2725

14. Kim BG, Kim GY, Cha JK. Pre-diabetes is a predictor of short-term poor outcomes after acute ischemic stroke using IV thrombolysis. *BMC Neurol*. 2021;21(1). doi:10.1186/s12883-021-02102-1

15. Li L, Pan Y, Wang M, et al. Trends and predictors of myocardial infarction or vascular death after ischaemic stroke or TIA in China, 2007-2018: Insights from China National Stroke Registries. *Stroke Vasc Neurol*. 2021;6(2):214-221. doi:10.1136/svn-2020-000503

16. López-De-andrés A, Jimenez-Garcia R, Hernández-Barrera V, et al. Sex-related disparities in the incidence and outcomes of ischemic stroke among type 2 diabetes patients. A matched-pair analysis using the spanish national hospital discharge database for years 2016–2018. *Int J Environ Res Public Health*. 2021;18(7). doi:10.3390/ijerph18073659

17. Macintosh BJ, Cohen E, Colby-Milley J, et al. Diabetes mellitus is associated with poor in-hospital and long-term outcomes in young and midlife stroke survivors. *J Am Heart Assoc*. 2021;10(14). doi:10.1161/JAHA.120.019991

18. Olaiya MT, Cadilhac DA, Kim J, et al. Quality of Care and One-Year Outcomes in Patients with Diabetes Hospitalised for Stroke or TIA: A Linked Registry Study. *Journal of Stroke and Cerebrovascular Diseases*. 2021;30(11). doi:10.1016/j.jstrokecerebrovasdis.2021.106083

19. Sagris D, Perlepe K, Leventis I, et al. Statin treatment and outcomes after embolic stroke of undetermined source. *Intern Emerg Med*. 2021;16(5):1261-1266. doi:10.1007/s11739-021-02743-7

20. The EZ, Ng MY, Ng GJ, et al. Long-term outcomes of ischaemic stroke patients with diabetes in a multi-ethnic cohort in Singapore. *Ann Acad Med Singap*. 2021;50(1):16-25. doi:10.47102/annals-acadmedsg.2020105

21. Zamir E, Libruder C, Murad H, et al. Diabetes associated risk for mortality increases with time among first stroke survivors - Findings from the Israeli National Stroke Registry. *J Diabetes Complications*. 2021;35(10). doi:10.1016/j.jdiacomp.2021.107999

22. Fang HJ, Pan YS, Wang YJ, Wang CX, Wang YL, Zhong LY. Prognostic value of admission hyperglycemia on outcomes of thrombolysis in ischemic stroke patients with or without diabetes. *Chin Med J (Engl)*. 2020;133(18):2244-2246. doi:10.1097/CM9.0000000000001005

23. Forti P, Maioli F, Nativio V, Maestri L, Coveri M, Zoli M. Association of prestroke glycemic status with stroke mortality. *BMJ Open Diabetes Res Care*. 2020;8(1). doi:10.1136/bmjdrc-2019-000957

24. Ouk M, Wu CY, Colby-Milley J, et al. Depression and Diabetes Mellitus Multimorbidity Is Associated With Loss of Independence and Dementia Poststroke. *Stroke*. 2020;51(12):3531-3540. doi:10.1161/STROKEAHA.120.031068

25. Szlachetka WA, Pana TA, Tiamkao S, et al. Impact of diabetes on complications, long term mortality and recurrence in 608,890 hospitalised patients with stroke. *Glob Heart*. 2020;15(1). doi:10.5334/GH.364

26. Yuan K, Chen J, Xu P, et al. A Nomogram for Predicting Stroke Recurrence Among Young Adults. *Stroke*. 2020;51(6):1865-1867. doi:10.1161/STROKEAHA.120.029740

27. Zhou Y, Pan Y, Yan H, et al. Triglyceride Glucose Index and Prognosis of Patients With Ischemic Stroke. *Front Neurol*. 2020;11. doi:10.3389/fneur.2020.00456

28. Akhtar N, Kamran S, Singh R, et al. The Impact of Diabetes on Outcomes After Acute Ischemic Stroke: A Prospective Observational Study. *Journal of Stroke and Cerebrovascular Diseases*. 2019;28(3):619-626. doi:10.1016/j.jstrokecerebrovasdis.2018.11.003

29. Choi KH, Kim JH, Kang KW, et al. HbA1c (Glycated Hemoglobin) Levels and Clinical Outcome Post-Mechanical Thrombectomy in Patients with Large Vessel Occlusion. *Stroke*. 2019;50(1):119-126. doi:10.1161/STROKEAHA.118.021598

30. Oliveira ADP de, Andrade-Valença LPA de, Valença MM. Factors Associated With In-Hospital Mortality in Very Elderly Patients With Ischemic Stroke: A Cohort Study. *Journal of Stroke and Cerebrovascular Diseases*. 2019;28(10). doi:10.1016/j.jstrokecerebrovasdis.2019.06.039

31. Mengel A, Ulm L, Hotter B, et al. Biomarkers of immune capacity, infection and inflammation are associated with poor outcome and mortality after stroke - The PREDICT study. *BMC Neurol*. 2019;19(1). doi:10.1186/s12883-019-1375-6

32. Panni P, Gory B, Xie Y, et al. Acute Stroke With Large Ischemic Core Treated by Thrombectomy: Predictors of Good Outcome and Mortality. *Stroke*. 2019;50(5):1164-1171. doi:10.1161/STROKEAHA.118.024295

33. Tsivgoulis G, Katsanos AH, Mavridis D, et al. Association of baseline hyperglycemia with outcomes of patients with and without diabetes with acute ischemic stroke treated with intravenous thrombolysis: A propensity score-matched analysis from the SITS-ISTR registry. *Diabetes*. 2019;68(9):1861-1869. doi:10.2337/db19-0440

34. Ago T, Matsuo R, Hata J, et al. Insulin resistance and clinical outcomes after acute ischemic stroke. *Neurology*. 2018;90(17):E1470-E1477. doi:10.1212/WNL.0000000000005358

35. Altavilla R, Anticoli S, Venti MP, et al. Clinical skills or high-tech MR in TIA patients: what makes the difference? *Neurological Sciences*. 2018;39(12):2091-2096. doi:10.1007/s10072-018-3546-4

36. Bauza C, Yeatts SD, Borg K, et al. Determining the joint effect of obesity and diabetes on functional disability at 3-months and on all-cause mortality at 1-year following an ischemic stroke. *BMC Endocr Disord*. 2018;18(1). doi:10.1186/s12902-018-0255-1

37. Bauza C, Martin R, Yeatts SD, et al. Determining the Joint Effect of Obesity and Diabetes on All-Cause Mortality and Cardiovascular-Related Mortality following an Ischemic Stroke. *Stroke Res Treat*. 2018;2018. doi:10.1155/2018/4812712

38. Chen G, Wang X, Robinson TG, et al. Comparative effects of low-dose versus standard-dose alteplase in ischemic patients with prior stroke and/or diabetes mellitus: The ENCHANTED trial. *J Neurol Sci*. 2018;387:1-5. doi:10.1016/j.jns.2018.01.014

39. Echouffo-Tcheugui JB, Xu H, Matsouaka RA, et al. Diabetes and long-term outcomes of ischaemic stroke: Findings from get with the guidelines-stroke. *Eur Heart J*. 2018;39(25):2376-2386. doi:http://dx.doi.org/10.1093/eurheartj/ehy036

40. Jin P, Matos Diaz I, Stein L, Thaler A, Tuhrim S, Dhamoon MS. Intermediate risk of cardiac events and recurrent stroke after stroke admission in young adults. *International Journal of Stroke*. 2018;13(6):576-584. doi:10.1177/1747493017733929

41. MacDougal EL, Herman WH, Wing JJ, Morgenstern LB, Lisabeth LD. Diabetes and ischaemic stroke outcome. *Diabetic Medicine*. 2018;35(9):1249-1257. doi:10.1111/dme.13665

42. Osei E, Fonville S, Zandbergen AAM, Koudstaal PJ, Dippel DWJ, den Hertog HM. Impaired fasting glucose is associated with unfavorable outcome in ischemic stroke patients treated with intravenous alteplase. *J Neurol*. 2018;265(6):1426-1431. doi:10.1007/s00415-018-8866-z

43. Read SH, McAllister DA, Colhoun HM, et al. Incident ischaemic stroke and Type 2 diabetes: trends in incidence and case fatality in Scotland 2004–2013. *Diabetic Medicine*. 2018;35(1):99-106. doi:10.1111/dme.13528

44. Sarfo FS, Akassi J, Kyem G, et al. Long-Term Outcomes of Stroke in a Ghanaian Outpatient Clinic. *J Stroke Cerebrovasc Dis*. 2018;27(4):1090-1099. doi:10.1016/j.jstrokecerebrovasdis.2017.11.017

45. Soriano-Reixach MM, Vivanco-Hidalgo RM, Ois A, Rodríguez-Campello A, Roquer J. Interaction of sex and diabetes on outcome after ischemic stroke. *Front Neurol*. 2018;9(APR). doi:10.3389/fneur.2018.00250

46. Zhou Y, Zhang Y, Pan Y, et al. Insulin Resistance Index from Oral Glucose Tolerance Test Predicts Ischemic Stroke Outcomes in Non-Diabetic Patients with Different Estimated Glomerular Filtration Rate Strata. *Cerebrovascular Diseases*. 2018;46(3-4):140-149. doi:10.1159/000492125

47. Bergström L, Irewall AL, Söderström L, Ögren J, Laurell K, Mooe T. One-Year Incidence, Time Trends, and Predictors of Recurrent Ischemic Stroke in Sweden From 1998 to 2010: An Observational Study. *Stroke*. 2017;48(8):2046-2051. doi:10.1161/STROKEAHA.117.016815

48. Chen W, Pan Y, Jing J, et al. Recurrent stroke in minor ischemic stroke or transient ischemic attack with metabolic syndrome and/or diabetes mellitus. *J Am Heart Assoc*. 2017;6(6). doi:10.1161/JAHA.116.005446

49. Corrao G, Rea F, Merlino L, Mazzola P, Annoni F, Annoni G. Management, prognosis and predictors of unfavourable outcomes in patients newly hospitalized for transient ischemic attack: A real-world investigation from Italy. *BMC Neurol*. 2017;17(1). doi:10.1186/s12883-017-0796-3

50. Jing J, Pan Y, Zhao X, et al. Prognosis of Ischemic Stroke with Newly Diagnosed Diabetes Mellitus According to Hemoglobin A1c Criteria in Chinese Population. *Stroke*. 2016;47(8):2038-2044. doi:10.1161/STROKEAHA.116.013606

51. Pan Y, Jing J, Chen W, et al. Post-glucose load measures of insulin resistance and prognosis of nondiabetic patients with ischemic stroke. *J Am Heart Assoc*. 2017;6(1). doi:10.1161/JAHA.116.004990

52. Pan Y, Meng X, Jing J, et al. Association of multiple infarctions and ICAS with outcomes of minor stroke and TIA. *Neurology*. 2017;88(11):1081-1088. doi:10.1212/WNL.0000000000003719

53. Pirinen J, Putaala J, Aarnio K, et al. Twelve-lead electrocardiogram and mortality in young adults after ischaemic stroke. *Eur Stroke J*. 2017;2(1):77-86. doi:10.1177/2396987316684706

54. Stefanovic Budimkic M, Pekmezovic T, Beslac-Bumbasirevic L, et al. Long-Term Prognosis in Ischemic Stroke Patients Treated with Intravenous Thrombolytic Therapy. *Journal of Stroke and Cerebrovascular Diseases*. 2017;26(1):196-203. doi:10.1016/j.jstrokecerebrovasdis.2016.09.009

55. Yoo J, Song D, Baek JH, et al. Poor long-term outcomes in stroke patients with asymptomatic coronary artery disease in heart CT. *Atherosclerosis*. 2017;265:7-13. doi:10.1016/j.atherosclerosis.2017.07.029

56. Kang K, Park TH, Kim N, et al. Recurrent Stroke, Myocardial Infarction, and Major Vascular Events during the First Year after Acute Ischemic Stroke: The Multicenter Prospective Observational Study about Recurrence and Its Determinants after Acute Ischemic Stroke i. *Journal of Stroke and Cerebrovascular Diseases*. 2016;25(3):656-664. doi:10.1016/j.jstrokecerebrovasdis.2015.11.036

57. Pan Y, Jing J, Li H, Wang Y, Wang Y, He Y. Abnormal glucose regulation increases stroke risk in minor ischemic stroke or TIA. *Neurology*. 2016;87(15):1551. doi:10.1212/WNL.0000000000003200

58. Pan Y, Wang Y, Li H, Gaisano HY, Wang Y, He Y. Association of diabetes and prognosis of minor stroke and its subtypes: A prospective observational study. *PLoS One*. 2016;11(4). doi:10.1371/journal.pone.0153178

59. Wood AD, Mannu GS, Clark AB, et al. Rheumatic mitral valve disease is associated with worse outcomes in stroke: A Thailand national database study. *Stroke*. 2016;47(11):2695-2701. doi:10.1161/STROKEAHA.116.014512

60. Cruz-Herranz A, Fuentes B, Martínez-Sánchez P, et al. Is diabetes an independent risk factor for in-hospital complications after a stroke? *J Diabetes*. 2015;7(5):657-663. doi:10.1111/1753-0407.12222

61. Li B, Wang T, Lou Y, et al. Sex Differences in Outcomes and Associated Risk Factors after Acute Ischemic Stroke in Elderly Patients: A Prospective Follow-up Study. *Journal of Stroke and Cerebrovascular Diseases*. 2015;24(10):2277-2284. doi:10.1016/j.jstrokecerebrovasdis.2015.06.007

62. Lin J, Zheng H, Cucchiara BL, et al. Association of Lp-PLA2-A and early recurrence of vascular events after TIA and minor stroke. *Neurology*. 2015;85(18):1585-1591. doi:10.1212/WNL.0000000000001938

63. Masrur S, Cox M, Bhatt DL, et al. Association of acute and chronic hyperglycemia with acute ischemic stroke outcomes post-thrombolysis: Findings from get with the guidelines-stroke. *J Am Heart Assoc*. 2015;4(10). doi:10.1161/JAHA.115.002193

64. Munoz-Rivas N, Mendez-Bailon M, Hernandez-Barrera V, et al. Time trends in ischemic stroke among type 2 diabetic and non-diabetic patients: Analysis of the Spanish national hospital discharge data (2003-2012). *PLoS One*. 2015;10(12). doi:10.1371/journal.pone.0145535

65. Policardo L, Seghieri G, Francesconi P, et al. Gender difference in diabetes-associated risk of first-ever and recurrent ischemic  stroke. *J Diabetes Complications*. 2015;29(5):713-717. doi:10.1016/j.jdiacomp.2014.12.008

66. Policardo L, Seghieri G, Anichini R, et al. Effect of diabetes on hospitalization for ischemic stroke and related in-hospital mortality: A study in Tuscany, Italy, over years 2004-2011. *Diabetes Metab Res Rev*. 2015;31(3):280-286. doi:10.1002/dmrr.2607

67. Serena J, Segura T, Roquer J, García-Gil M, Castillo J. The ARTICO study: Identification of patients at high risk of vascular recurrence after a first non-cardioembolic stroke. *BMC Neurol*. 2015;15(1). doi:10.1186/s12883-015-0278-4

68. Wu L, Wang A, Wang X, et al. Factors for short-term outcomes in patients with a minor stroke: Results from China National Stroke Registry. *BMC Neurol*. 2015;15(1). doi:10.1186/s12883-015-0505-z

69. Zhang C, Zhao X, Wang C, et al. Prediction factors of recurrent ischemic events in one year after minor stroke. *PLoS One*. 2015;10(3). doi:10.1371/journal.pone.0120105

70. Aarnio K, Haapaniemi E, Melkas S, Kaste M, Tatlisumak T, Putaala J. Long-term mortality after first-ever and recurrent stroke in young adults. *Stroke*. 2014;45(9):2670-2676. doi:10.1161/STROKEAHA.114.005648

71. Arnold M, Mattle S, Galimanis A, et al. Impact of admission glucose and diabetes on recanalization and outcome after intra-arterial thrombolysis for ischaemic stroke. *International Journal of Stroke*. 2014;9(8):985-991. doi:10.1111/j.1747-4949.2012.00879.x

72. Erdur H, Scheitz JF, Grittner U, Laufs U, Endres M, Nolte CH. Heart rate on admission independently predicts in-hospital mortality in acute ischemic stroke patients. *Int J Cardiol*. 2014;176(1):206-210. doi:10.1016/j.ijcard.2014.07.001

73. Hjalmarsson C, Manhem K, Bokemark L, Andersson B. The Role of Prestroke Glycemic Control on Severity and Outcome of Acute Ischemic Stroke. *Stroke Res Treat*. 2015;2014. doi:10.1155/2014/694569

74. Jia Q, Liu G, Zheng H, et al. Impaired Glucose Regulation Predicted 1-Year Mortality of Chinese Patients With Ischemic Stroke. *Stroke*. 2014;45(5):1498-1500. doi:10.1161/STROKEAHA.113.002977

75. Pezzini A, Grassi M, Lodigiani C, et al. Predictors of long-term recurrent vascular events after ischemic stroke at young age: The Italian project on stroke in young adults. *Circulation*. 2014;129(16):1668-1676. doi:10.1161/CIRCULATIONAHA.113.005663

76. Roquer J, Rodríguez-Campello A, Cuadrado-Godia E, et al. Ischemic stroke in prediabetic patients. *J Neurol*. 2014;261(10):1866-1870. doi:10.1007/s00415-014-7431-7

77. L.C.A. RJ, P.A.J. K, R.M. A, et al. High incidence of diabetes after stroke in young adults and risk of recurrent vascular events: The FUTURE study. *PLoS One*. 2014;9(1):e87171. doi:http://dx.doi.org/10.1371/journal.pone.0087171

78. Toni D, Di Angelantonio E, Di Mascio MT, Vinisko R, Bath PMW. Types of stroke recurrence in patients with ischemic stroke: A substudy from the PRoFESS trial. *International Journal of Stroke*. 2014;9(7):873-878. doi:10.1111/ijs.12150

79. Wu S, Wang C, Jia Q, et al. HbA1c is associated with increased all-cause mortality in the first year after acute ischemic stroke. *Neurol Res*. 2014;36(5):444-452. doi:10.1179/1743132814Y.0000000355

80. Zhao L, Du W, Zhao X, et al. Favorable functional recovery in overweight ischemic stroke survivors: Findings from the China National Stroke Registry. *Journal of Stroke and Cerebrovascular Diseases*. 2014;23(3). doi:10.1016/j.jstrokecerebrovasdis.2013.10.002

81. Balti EV, Kengne AP, Fokouo JVF, Nouthé BE, Sobngwi E. Metabolic Syndrome and Fatal Outcomes in the Post-Stroke Event: A 5-Year Cohort Study in Cameroon. *PLoS One*. 2013;8(4). doi:10.1371/journal.pone.0060117

82. Gomes J, Damasceno A, Carrilho C, et al. Determinants of early case-fatality among stroke patients in Maputo, Mozambique and impact of in-hospital complications. *International Journal of Stroke*. 2013;8(100 A):69-75. doi:10.1111/j.1747-4949.2012.00957.x

83. Hanchate AD, Schwamm LH, Huang W, Hylek EM. Comparison of Ischemic Stroke Outcomes and Patient and Hospital Characteristics by Race/Ethnicity and Socioeconomic Status. *Stroke*. 2013;44(2):469-476. doi:10.1161/STROKEAHA.112.669341

84. Rocco A, Heuschmann PU, Schellinger PD, et al. Glycosylated hemoglobin a1 predicts risk for symptomatic hemorrhage after thrombolysis for acute stroke. *Stroke*. 2013;44(8):2134-2138. doi:10.1161/STROKEAHA.111.675918

85. Wang Y, Xu J, Zhao X, et al. Association of hypertension with stroke recurrence depends on ischemic stroke subtype. *Stroke*. 2013;44(5):1232-1237. doi:10.1161/STROKEAHA.111.000302

86. Wang Z, Li J, Wang C, et al. Gender Differences in 1-Year Clinical Characteristics and Outcomes after Stroke: Results from the China National Stroke Registry. *PLoS One*. 2013;8(2). doi:10.1371/journal.pone.0056459

87. Fuentes B, Martínez-Sánchez P, Alonso de Leciñana M, et al. Diabetes and previous stroke: Hazards for intravenous thrombolysis? *Eur J Neurol*. 2012;19(4):587-593. doi:10.1111/j.1468-1331.2011.03576.x

88. Purroy F, Caballero PEJ, Gorospe A, et al. Prediction of early stroke recurrence in transient ischemic attack patients from the PROMAPA study: A comparison of prognostic risk scores. *Cerebrovascular Diseases*. 2012;33(2):182-189. doi:10.1159/000334771

89. Tsivgoulis G, Bogiatzi C, Heliopoulos I, et al. Low Ankle-Brachial Index predicts early risk of recurrent stroke in patients with acute cerebral ischemia. *Atherosclerosis*. 2012;220(2):407-412. doi:10.1016/j.atherosclerosis.2011.11.009

90. Andersen KK, Andersen ZJ, Olsen TS. Predictors of early and late case-fatality in a nationwide danish study of 26 818 patients with first-ever ischemic stroke. *Stroke*. 2011;42(10):2806-2812. doi:10.1161/STROKEAHA.111.619049

91. Burns JD, Rabinstein AA, Roger VL, et al. Incidence and predictors of myocardial infarction after transient ischemic attack: A population-based study. *Stroke*. 2011;42(4):935-940. doi:10.1161/STROKEAHA.110.593723

92. Greisenegger S, Zehetmayer S, Ferrari J, et al. Clinical predictors of death in young and middle-aged patients with ischemic stroke or transient ischemic attack: Long-term results of the Vienna Stroke Registry : redictors of ischemic stroke mortality in patients 60 years<. *J Neurol*. 2011;258(6):1105-1113. doi:10.1007/s00415-010-5893-9

93. Hassan Y, Al-Jabi SW, Aziz NA, Looi I, Zyoud SH. Statin use prior to ischemic stroke onset is associated with decreased in-hospital mortality. *Fundam Clin Pharmacol*. 2011;25(3):388-394. doi:10.1111/j.1472-8206.2010.00846.x

94. Jia Q, Zhao X, Wang C, et al. Diabetes and poor outcomes within 6 months after acute ischemic stroke: the China  National Stroke Registry. *Stroke*. 2011;42(10):2758-2762. doi:10.1161/STROKEAHA.111.621649

95. Li HW, Yang MC, Chung KP. Predictors for readmission of acute ischemic stroke in Taiwan. *Journal of the Formosan Medical Association*. 2011;110(10):627-633. doi:10.1016/j.jfma.2011.08.004

96. Winell K, Pääkkönen R, Pietilä A, Reunanen A, Niemi M, Salomaa V. Prognosis of ischaemic stroke is improving similarly in patients with type 2 diabetes as in nondiabetic patients in Finland. *International Journal of Stroke*. 2011;6(4):295-301. doi:10.1111/j.1747-4949.2010.00567.x

97. Ahmed N, Dá A, Eriksson N, et al. *Association of Admission Blood Glucose and Outcome in Patients Treated With Intravenous Thrombolysis: Results From the Safe Implementation of Treatments in Stroke International Stroke Thrombolysis Register (SITS-ISTR)*. Vol 67.; 2010. https://jamanetwork.com/

98. Fonarow GC, Reeves MJ, Smith EE, et al. Characteristics, performance measures, and in-hospital outcomes of the first one million stroke and transient ischemic attack admissions in Get with the guidelines-stroke. *Circ Cardiovasc Qual Outcomes*. 2010;3(3):291-302. doi:10.1161/CIRCOUTCOMES.109.921858

99. Hassan Y, Aziz NA, Al-Jabi SW, Looi I, Zyoud SH. Impact of angiotensin-converting enzyme inhibitors administration prior to acute  ischemic stroke onset on in-hospital mortality. *J Cardiovasc Pharmacol Ther*. 2010;15(3):274-281. doi:10.1177/1074248410373751

100. Koton S, Tanne D, Green MS, Bornstein NM. Mortality and predictors of death 1 month and 3 years after first-ever ischemic stroke: Data from the first National Acute Stroke Israeli Survey (NASIS 2004). *Neuroepidemiology*. 2010;34(2):90-96. doi:10.1159/000264826

101. Meurer WJ, Scott PA, Caveney AF, et al. Lack of association between hyperglycaemia at arrival and clinical outcomes in acute stroke patients treated with tissue plasminogen activator. *International Journal of Stroke*. 2010;5(3):163-166. doi:10.1111/j.1747-4949.2010.00425.x

102. Nakajima M, Hirano T, Naritomi H, Minematsu K. Symptom progression or fluctuation in transient ischemic attack patients predicts subsequent stroke. *Cerebrovascular Diseases*. 2010;29(3):221-227. doi:10.1159/000267844

103. Putaala J, Haapaniemi E, Metso AJ, et al. Recurrent ischemic events in young adults after first-ever ischemic stroke. *Ann Neurol*. 2010;68(5):661-671. doi:10.1002/ana.22091

104. Putaala J, Sairanen T, Meretoja A, et al. Post-thrombolytic hyperglycemia and 3-month outcome in acute ischemic stroke. *Cerebrovascular Diseases*. 2010;31(1):83-92. doi:10.1159/000321332

105. Reeves MJ, Vaidya RS, Fonarow GC, et al. Quality of care and outcomes in patients with diabetes hospitalized with ischemic  stroke: findings from Get With the Guidelines-Stroke. *Stroke*. 2010;41(5):e409-17. doi:10.1161/STROKEAHA.109.572693

106. Smith EE, Shobha N, Dai D, et al. Risk score for in-hospital ischemic stroke mortality derived and validated within the get with the guidelines-stroke program. *Circulation*. 2010;122(15):1496-1504. doi:10.1161/CIRCULATIONAHA.109.932822

107. Spengos K, Vemmos K. Risk factors, etiology, and outcome of first-ever ischemic stroke in young adults aged 15 to 45 - the Athens young stroke registry. *Eur J Neurol*. 2010;17(11):1358-1364. doi:10.1111/j.1468-1331.2010.03065.x

108. Wu B, Lin S, Hao Z, et al. Proportion, risk factors and outcome of lacunar infarction: A hospital-based study in a Chinese population. *Cerebrovascular Diseases*. 2010;29(2):181-187. doi:10.1159/000267277

109. Busch MA, Lutz K, Röhl JE, Neuner B, Masuhr F. Low ankle-brachial index predicts cardiovascular risk after acute ischemic stroke or transient ischemic attack. *Stroke*. 2009;40(12):3700-3705. doi:10.1161/STROKEAHA.109.559740

110. Gulli G, Khan S, Markus HS. Vertebrobasilar stenosis predicts high early recurrent stroke risk in posterior circulation stroke and TIA. *Stroke*. 2009;40(8):2732-2737. doi:10.1161/STROKEAHA.109.553859

111. Oksala NKJ, Oksala A, Pohjasvaara T, et al. Age related white matter changes predict stroke death in long term follow-up. *J Neurol Neurosurg Psychiatry*. 2009;80(7):762-766. doi:10.1136/jnnp.2008.154104

112. Putaala J, Curtze S, Hiltunen S, Tolppanen H, Kaste M, Tatlisumak T. Causes of death and predictors of 5-year mortality in young adults after first-ever ischemic stroke: The Helsinki young stroke registry. *Stroke*. 2009;40(8):2698-2703. doi:10.1161/STROKEAHA.109.554998

113. Sun Y, Toh MPHS. Impact of diabetes mellitus (DM) on the health-care utilization and clinical outcomes of patients with stroke in Singapore. *Value in Health*. 2009;12(SUPPL. 3). doi:10.1111/j.1524-4733.2009.00639.x

114. Yokota C, Minematsu K, Ito A, Toyoda K, Nagasawa H, Yamaguchi T. Albuminuria, but not metabolic syndrome, is a significant predictor of stroke recurrence in ischemic stroke. *J Neurol Sci*. 2009;277(1-2):50-53. doi:10.1016/j.jns.2008.10.002

115. Gunarathne A, Patel J V., Potluri R, et al. Increased 5-year mortality in the migrant South Asian stroke patients with diabetes mellitus in the United Kingdom: The West Birmingham Stroke Project. *Int J Clin Pract*. 2008;62(2):197-201. doi:10.1111/j.1742-1241.2007.01580.x

116. Kamalesh M, Shen J, Eckert GJ. Long term postischemic stroke mortality in diabetes: A veteran cohort analysis. *Stroke*. 2008;39(10):2727-2731. doi:10.1161/STROKEAHA.108.517441

117. Staals J, Van Raak L, Hilton A, Lodder J. Differences in long-term survival in two lacunar stroke types: A 15-year follow-up study in 782 cerebral infarct patients. *Cerebrovascular Diseases*. 2008;25(1-2):26-31. doi:10.1159/000111496

118. Han JH, Ho SSY, Lam WWM, Wong KS. Total cerebral blood flow estimated by color velocity imaging quantification ultrasound: A predictor for recurrent stroke? *Journal of Cerebral Blood Flow and Metabolism*. 2007;27(4):850-856. doi:10.1038/sj.jcbfm.9600392

119. Johnston SC, Rothwell PM, Nguyen-Huynh MN, et al. Validation and refinement of scores to predict very early stroke risk after transient ischaemic attack. *Lancet*. 2007;369(9558):283-292. doi:10.1016/S0140-6736(07)60150-0

120. Prosser J, MacGregor L, Lees KR, Diener HC, Hacke W, Davis S. Predictors of early cardiac morbidity and mortality after ischemic stroke. *Stroke*. 2007;38(8):2295-2302. doi:10.1161/STROKEAHA.106.471813

121. Roquer J, Ois A, Rodríguez Campello A, et al. Clustering of vascular risk factors and in-hospital death after acute ischemic stroke. *J Neurol*. 2007;254(12):1636-1641. doi:10.1007/s00415-007-0559-y

122. Toyoda K, Okada Y, Kobayashi S. Early recurrence of ischemic stroke in Japanese patients: the Japan standard stroke  registry study. *Cerebrovasc Dis*. 2007;24(2-3):289-295. doi:10.1159/000105682

123. Bateman BT, Schumacher HC, Boden-Albala B, et al. Factors associated with in-hospital mortality after administration of thrombolysis in acute ischemic stroke patients: An analysis of the nationwide inpatient sample 1999 to 2002. *Stroke*. 2006;37(2):440-446. doi:10.1161/01.STR.0000199851.24668.f1

124. Hamidon BB, Nabil I, Raymond AA. Risk factors and outcome of dysphagia after an acute ischaemic stroke. *Med J Malaysia*. 2006;61(5):553-557.

125. Kammersgaard LP, Olsen TS. Cardiovascular risk factors and 5-year mortality in the Copenhagen Stroke Study. *Cerebrovascular Diseases*. 2006;21(3):187-193. doi:10.1159/000090531

126. Mateo I, Pinedo A, Escalza I, Garcia-Monco JC. Laterality does not influence early mortality in MCA ischemic stroke. *Clin Neurol Neurosurg*. 2006;108(7):628-631. doi:10.1016/j.clineuro.2005.10.002

127. Sacco S, Marini C, Totaro R, Russo T, Cerone D, Carolei A. A population-based study of the incidence and prognosis of lacunar stroke. *Neurology*. 2006;66(9):1335-1338. doi:10.1212/01.wnl.0000210457.89798.0e

128. Tsivgoulis G, Spengos K, Manta P, et al. Validation of the ABCD score in identifying individuals at high early risk of stroke after a transient ischemic attack: A hospital-based case series study. *Stroke*. 2006;37(12):2892-2897. doi:10.1161/01.STR.0000249007.12256.4a

129. Vermeer SE, Sandee W, Algra A, Koudstaal PJ, Kappelle LJ, Dippel DWJ. Impaired glucose tolerance increases stroke risk in nondiabetic patients with transient ischemic attack or minor ischemic stroke. *Stroke*. 2006;37(6):1413-1417. doi:10.1161/01.STR.0000221766.73692.0b

130. Xu G, Liu X, Wu W, Zhang R, Yin Q. Recurrence after ischemic stroke in chinese patients: Impact of uncontrolled modifiable risk factors. *Cerebrovascular Diseases*. 2007;23(2-3):117-120. doi:10.1159/000097047

131. Fu JH, Lu CZ, Hong Z, Dong Q, Luo Y, Wong KS. Extent of white matter lesions is related to acute subcortical infarcts and predicts further stroke risk in patients with first ever ischaemic stroke. *J Neurol Neurosurg Psychiatry*. 2005;76(6):793-796. doi:10.1136/jnnp.2003.032771

132. Kaplan RC, Tirschwell DL, Longstreth WT, et al. Vascular events, mortality, and preventive therapy following ischemic stroke in the elderly. *Neurology*. 2005;65(6):835-842. doi:10.1212/01.wnl.0000176058.09848.bb

133. Kimura K, Minematsu K, Kazui S, Yamaguchi T. Mortality and cause of death after hospital discharge in 10,981 patients with ischemic stroke and transient ischemic attack. *Cerebrovascular Diseases*. 2005;19(3):171-178. doi:10.1159/000083252

134. Rothwell P, Giles M, Flossmann E, et al. A simple score (ABCD) to identify individuals at high early risk of stroke after transient ischaemic attack. *The Lancet*. 2005;366(9479):29-36. doi:10.1016/S0140-6736(05)66702-5

135. Van Wijk I, Kappelle LJ, Van Gijn J, et al. Long-term survival and vascular event risk after transient ischaemic attack or minor ischaemic stroke: A cohort study. *Lancet*. 2005;365(9477):2098-2104. doi:10.1016/S0140-6736(05)66734-7

136. Kaarisalo MM, Räihä I, Sivenius J, et al. Diabetes worsens the outcome of acute ischemic stroke. *Diabetes Res Clin Pract*. 2005;69(3):293-298. doi:10.1016/j.diabres.2005.02.001

137. Heuschmann PU, Kolominsky-Rabas PL, Misselwitzet B. Predictors of in-hospital mortality and attributable risks of death after ischemic stroke: The German Stroke Registers Study Group. *ACC Curr J Rev*. 2004;13(12):19-20. doi:10.1016/j.accreview.2004.11.080

138. Hill MD, Yiannakoulias N, Jeerakathil T, Tu J V., Svenson LW, Schopflocher DP. The high risk of stroke immediately after transient ischemic attack: A population-based study. *Neurology*. 2004;62(11):2015-2020. doi:10.1212/01.WNL.0000129482.70315.2F

139. Lee AH, Somerford PJ, Yau KKW. Risk factors for ischaemic stroke recurrence after hospitalisation. *Medical Journal of Australia*. 2004;181(5):244-246. doi:10.5694/j.1326-5377.2004.tb06261.x

140. Soda T, Nakayasu H, Maeda M, et al. Stroke recurrence within the first year following cerebral infarction - Tottori University Lacunar Infarction Prognosis Study (TULIPS). *Acta Neurol Scand*. 2004;110(6):343-349. doi:10.1111/j.1600-0404.2004.00290.x

141. Varona JF, Bermejo F, Guerra JM, Molina JA. Long-term prognosis of ischemic stroke in young adults: Study of 272 cases. *J Neurol*. 2004;251(12):1507-1514. doi:10.1007/s00415-004-0583-0

142. Conway DSG, Lip GYH. Ethnicity in relation to atrial fibrillation and stroke (the West Birmingham Stroke Project). *American Journal of Cardiology*. 2003;92(12):1476-1479. doi:10.1016/j.amjcard.2003.08.065

143. De Jong G, Van Raak L, Kessels F, Lodder J. Stroke subtype and mortality: A follow-up study in 998 patients with a first cerebral infarct. *J Clin Epidemiol*. 2003;56(3):262-268. doi:10.1016/S0895-4356(02)00572-3

144. Hamidon BB, Raymond AA. The impact of diabetes mellitus on in-hospital stroke mortality. *J Postgrad Med*. 2003;49(4):307-310.

145. Lee AH, Somerford PJ, Yau KKW. Factors influencing survival after stroke in Western Australia. *Medical Journal of Australia*. 2003;179(6):289-293. doi:10.5694/j.1326-5377.2003.tb05549.x

146. Wong KS, Li H. Long-term mortality and recurrent stroke risk among Chinese stroke patients with predominant intracranial atherosclerosis. *Stroke*. 2003;34(10):2361-2366. doi:10.1161/01.STR.0000089017.90037.7A

147. De Jong G, Kessels F, Lodder J. Two types of lacunar infarcts: Further arguments from a study on prognosis. *Stroke*. 2002;33(8):2072-2076. doi:10.1161/01.STR.0000022807.06923.A3

148. Johnston SC, Gress DR, Browner WS, Sidney S. Short-term Prognosis After Emergency Department Diagnosis of TIA. *JAMA*. 2000;284(22):2901-2906.

149. Petty GW, Brown RD, Whisnant JP, Sicks JD, O’Fallon WM, Wiebers DO. Ischemic Stroke Subtypes. *Stroke*. 2000;31(5):1062-1068. doi:10.1161/01.str.31.5.1062

150. Wong KS, Li H, Chan YL, et al. Use of transcranial doppler ultrasound to predict outcome in patients with intracranial large-artery occlusive disease. *Stroke*. 2000;31(11):2641-2647. doi:10.1161/01.STR.31.11.2641

151. Marini C, Totaro R, Carolei A. Long-term prognosis of cerebral ischemia in young adults. *Stroke*. 1999;30(11):2320-2325. doi:10.1161/01.STR.30.11.2320

152. Samsa GP, Bian J, Lipscomb J, Matchar DB. *Epidemiology of Recurrent Cerebral Infarction A Medicare Claims-Based Comparison of First and Recurrent Strokes on 2-Year Survival and Cost*.; 1999. http://ahajournals.org

153. Wong KS. Risk factors for early death in acute ischemic stroke and intracerebral hemorrhage:  A prospective hospital-based study in Asia. Asian Acute Stroke Advisory Panel. *Stroke*. 1999;30(11):2326-2330. doi:10.1161/01.str.30.11.2326

154. Clavier I, Hommel M, Besson G, Noèlle B, Perret JE. Long-term prognosis of symptomatic lacunar infarcts. A hospital-based study. *Stroke*. 1994;25(10):2005-2009. doi:10.1161/01.str.25.10.2005

155. Hier DB, Foulkes MA, Swiontoniowski M, et al. Stroke recurrence within 2 years after ischemic infarction. *Stroke*. 1991;22(2):155-161. doi:10.1161/01.str.22.2.155

156. Howard G, Toole JF, Becker C, et al. Changes in survival following stroke in five north carolina counties observed during two different periods. *Stroke*. 1989;20(3):345-350. doi:10.1161/01.STR.20.3.345

157. Howard G, Toole JF, Frye-Pierson J, Hinshelwood LC. Factors influencing the survival of 451 transient ischemic attack patients. *Stroke*. 1987;18(3):552-557. doi:10.1161/01.str.18.3.552

158. Rutten-Jacobs LCA, Maaijwee NAM, Arntz RM, et al. Long-term risk of recurrent vascular events after young stroke: The FUTURE study. *Ann Neurol*. 2013;74(4):592-601. doi:10.1002/ana.23953

159. Jing J, Pan Y, Zhao X, et al. Insulin Resistance and Prognosis of Nondiabetic Patients with Ischemic Stroke: The ACROSS-China Study (Abnormal Glucose Regulation in Patients with Acute Stroke Across China). *Stroke*. 2017;48(4):887-893. doi:10.1161/STROKEAHA.116.015613

160. Tu WJ, Liu Z, Chao BH, et al. Metformin use is associated with low risk of case fatality and disability rates  in first-ever stroke patients with type 2 diabetes. *Ther Adv Chronic Dis*. 2022;13:20406223221076896. doi:10.1177/20406223221076894

161. Tsivgoulis G, Goyal N, Iftikhar S, et al. Sulfonylurea Pretreatment and In-Hospital Use Does Not Impact Acute Ischemic Strokes  (AIS) Outcomes Following Intravenous Thrombolysis. *J Stroke Cerebrovasc Dis*. 2017;26(4):795-800. doi:10.1016/j.jstrokecerebrovasdis.2016.10.019

162. Favilla CG, Mullen MT, Ali M, Higgins P, Kasner SE. Sulfonylurea use before stroke does not influence outcome. *Stroke*. 2011;42(3):710-715. doi:10.1161/STROKEAHA.110.599274

163. Horsdal HT, Mehnert F, Rungby J, Johnsen SP. Type of preadmission antidiabetic treatment and outcome among patients with ischemic  stroke: a nationwide follow-up study. *J Stroke Cerebrovasc Dis*. 2012;21(8):717-725. doi:10.1016/j.jstrokecerebrovasdis.2011.03.007

164. Yoo J, Jeon J, Baik M, Kim J. Lobeglitazone, a novel thiazolidinedione, for secondary prevention in patients with ischemic stroke: a nationwide nested case-control study. *Cardiovasc Diabetol*. 2023;22(1):106. doi:10.1186/s12933-023-01841-4

165. Woo MH, Lee HS, Kim J. Effect of pioglitazone in acute ischemic stroke patients with diabetes mellitus: a  nested case-control study. *Cardiovasc Diabetol*. 2019;18(1):67. doi:10.1186/s12933-019-0874-5

166. Morgan CL, Inzucchi SE, Puelles J, Jenkins‐Jones S, Currie CJ. Impact of treatment with pioglitazone on stroke outcomes: A real‐world database analysis. *Diabetes Obes Metab*. 2018;20(9):2140-2147. doi:10.1111/dom.13344

167. Chen DY, Wang SH, Mao CT, et al. Sitagliptin After Ischemic Stroke in Type 2 Diabetic Patients: A Nationwide Cohort  Study. *Medicine*. 2015;94(28):e1128. doi:10.1097/MD.0000000000001128

168. Chen DY, Li YR, Mao CT, et al. Cardiovascular outcomes of vildagliptin in patients with type 2 diabetes mellitus  after acute coronary syndrome or acute ischemic stroke. *J Diabetes Investig*. 2020;11(1):110-124. doi:10.1111/jdi.13078

169. Li YR, Tsai SS, Chen DY, et al. Linagliptin and cardiovascular outcomes in type 2 diabetes after acute coronary  syndrome or acute ischemic stroke. *Cardiovasc Diabetol*. 2018;17(1):2. doi:10.1186/s12933-017-0655-y
